# Explainable artificial intelligence reveals key surgical parameters in robot-assisted and open radical prostatectomy

**DOI:** 10.1038/s41598-026-68074-9

**Published:** 2026-08-26

**Authors:** Christian R. Klein, Pia Heuser, Elena Trunz, Glen Kristiansen, Peter Brossart, Manuel Ritter, Philipp Krausewitz

**Affiliations:** 1 Clinic of Internal Medicine III, Department of Oncology, Hematology, Immune-Oncology and Rheumatology, University Hospital Bonn, Bonn, Germany; 2 Department of Visual Computing, Institute for Computer Science, University of Bonn, Bonn, Germany; 3 Department of Urology, University Hospital Bonn, Bonn, Germany; 4 Institute of Pathology, University Hospital Bonn, Bonn, Germany; 5 Center for Integrated Oncology Aachen Bonn Cologne Duesseldorf (CIO ABCD), Bonn, Germany

**Keywords:** Robot‑assisted laparoscopic radical prostatectomy, Predictive models, Shapley value, Machine learning, Prostate cancer, Cancer, Computational biology and bioinformatics, Medical research, Oncology

## Abstract

Preoperative risk stratification for radical prostatectomy is crucial, yet predicting the wide range of postoperative outcomes remains a significant challenge. While machine learning (ML) shows promise, “black box” models limit clinical translatability. This study aimed to predict postoperative parameters using ML and employ explainable AI (XAI) to identify their key clinical drivers. In a retrospective study of 326 patients (224 robot-assisted [RARP], 102 open [ORP]), we developed predictive models for twelve outcomes, including length of stay and pathological ISUP grade. Four ML algorithms (Random Forest, Gradient Boosting, SVM, Neural Network) were evaluated via nested 5-fold cross-validation. A custom permutation-based Shapley sampling framework SHAP (SHapley Additive exPlanations) was applied to the best-performing models to quantify the predictive importance of preoperative features. ML models outperformed baseline heuristics for a subset of the prespecified outcomes, with strongest performance for postoperative hemoglobin (R^2^ up to 0.57) and the decision to perform frozen sections (AUC up to 0.89). Not all outcomes proved equally amenable to prediction, consistent with the heterogeneous nature of postoperative recovery. SHAP analysis revealed a clear dichotomy: procedural parameters, such as catheter dwell time and hospital stay, were almost exclusively predicted by the surgical approach (RARP vs. ORP). In contrast, pathological outcomes like ISUP grade were predominantly driven by preoperative tumor characteristics. Preoperative hemoglobin was identified as a strong predictive feature for postoperative anemia within this dataset, ranking above non-modifiable factors such as age. Explainable AI can deconstruct the complex interplay of factors influencing surgical success, providing a data-driven basis for hypothesis generation and clinical pathway optimization. As a single-centre proof-of-concept study without external validation, these findings require prospective confirmation in independent multi-centre cohorts before clinical translation can be considered.

**Supplementary Information:**

The online version contains supplementary material available at 10.1038/s41598-026-68074-9.

## Introduction

Prostate cancer is the most common malignancy in men, with the majority of cases diagnosed at a localized and operable stage^1,2^. Radical prostatectomy (RP) represents a cornerstone of curative treatment. The procedure can be performed as a conventional open radical prostatectomy (ORP), most commonly via a retropubic (radical retropubic prostatectomy, RRP) or perineal (radical perineal prostatectomy, RPP) approach, or as a minimally invasive, robot-assisted radical prostatectomy (RARP)^3^. While effective, any surgical intervention carries an inherent risk of complications and variable patient outcomes, making the preoperative prediction of these results a significant clinical challenge^4,5^. In recent years, machine learning (ML) has emerged as a powerful tool for predictive tasks in medicine^6–12^. Within urology, ML models have been successfully applied to predict histopathological findings from biopsy or imaging data^13–20^. However, the prediction of a broader spectrum of clinical and procedural outcomes following radical prostatectomy remains largely unexplored^21^. A further critical limitation of existing work is that most ML models are treated as “black boxes,” which prevents a deeper understanding of the underlying predictive factors and limits the clinical translatability of their findings^22–26^. To address these gaps, the aim of this study was twofold: First, we aimed to determine whether a wide range of intraoperative events, postoperative outcomes, and pathological findings could be accurately predicted from routinely collected preoperative data using a supervised machine learning approach. Second, we aimed to move beyond mere prediction by employing explainable AI (XAI) methods, specifically SHAP (SHapley Additive exPlanations), to quantify the contribution of each individual input feature^27,28^. By doing so, we sought to identify the key clinical drivers of postoperative outcomes, thereby translating complex model predictions into transparent and clinically actionable insights.

## Methods

Our study design to investigate machine learning of surgical parameters in robot-assisted and open radical prostatectomy consists of two parts:

### Clinical study

#### Design and data source

Clinical data were collected from men who underwent open (ORP) or robot-assisted radical prostatectomy (RARP) between 2015 and 2020 in the Department of Urology, University Hospital Bonn, Bonn, Germany. Only data obtained as part of routine clinical care were included in the analysis. Patients with prior radiation therapy or high-intensity focused ultrasound (HIFU) were excluded. Neoadjuvant androgen deprivation therapy (ADT), alone or in combination with androgen receptor–targeted agents (e.g., enzalutamide), was permitted. There were no exclusion criteria regarding age or non-urological previous diseases. Postoperative follow-up was conducted through routine clinical visits at the treatment center for 12 months.

#### Surgical procedures

Both RARP and ORP were performed by experienced surgeons, with pelvic lymph node dissection conducted when indicated by the patient’s risk profile. RARP was carried out using a transperitoneal approach with the four-armed da Vinci system under general anesthesia. The procedure involved bladder detachment, endopelvic fascial incision, control of the dorsal venous complex, dissection of the bladder neck, vas deferens, seminal vesicles, and prostate, followed by reconstruction and vesicourethral anastomosis. ORP was performed via a retropubic approach under general anesthesia. After midline incision and exposure of the retropubic space, the dorsal venous complex was ligated and the prostate dissected according to standard technique, followed by reconstruction and vesicourethral anastomosis.

#### Postoperative monitoring and follow-up

Patients were followed up for 12 months after surgery. The postoperative parameters collected are summarized in Supplementary Table S2. In addition to surgical complications up to 30 days after surgery (Clavien Dindo score^29^), PSA levels were recorded after 3 months. A urological follow-up was performed after 12 months, during which stress incontinence, erectile dysfunction, and sexual medicine parameters (sexual intercourse: libido, quality, with/without aids) were recorded.

#### Ethical statement

The study was conducted in accordance with the Declaration of Helsinki and was approved by the Ethics Committee of the Medical Faculty of the University of Bonn (identification number: 477/20). The need for written informed consent was waived by the ethics committee due to the retrospective nature of the analysis of anonymized routine clinical data. All data were anonymised in accordance with EU Regulation 2016/679 (General Data Protection Regulation).

#### Input features and outcome variables

The parameters collected during the clinical study were categorized into two sets. Preoperative parameters served as the input features for the machine learning models. A detailed list of all input features, including their value ranges and scales, is provided in Table 1. Notably, parameters related to the preservation of the neurovascular bundle (NVB) (yes/no; unilateral/bilateral) were included as preoperative input features. At our institution, the decision to perform nerve sparing is made preoperatively in the course of a structured patient consultation, integrating tumor characteristics (PSA, biopsy results, MRI findings), erectile function, and patient preference. Intraoperative deviation from this plan occurs only in cases of clinically significant unexpected findings, such as macroscopic extracapsular extension, and represents the exception rather than the standard clinical workflow. The nerve-sparing decision therefore reflects a preoperative plan and its inclusion as a preoperative input feature is clinically justified. We acknowledge, however, that this decision inherently encodes a complex preoperative risk stratification and that the proportion of cases in which intraoperative findings led to a deviation from the planned approach is not systematically documented in our dataset. To assess the robustness of our findings with respect to this variable, we conducted a full sensitivity analysis excluding all nerve-sparing-related input features, with direct comparison of model performance with and without these variables. The results are presented in the Supplementary Material (Supplementary Tables S4-S8). Intra- and postoperative parameters were defined as the target outcomes for prediction. These included a range of surgical complications (e.g., urinoma, lymphocele, Clavien-Dindo score), procedural metrics, procedural/pathological composite endpoints (e.g., the combined frozen-section utilization/result category), and follow-up data (e.g., PSA level at 3 months, continence and sexual function at 12 months). A comprehensive list and detailed description of all target outcome variables are provided in Supplementary Table S2. Of the full set of candidate target variables collected in this study, outcomes were included in the main analysis if they met the following pre-specified methodological criteria:

1. Sufficient variance in the target variable across the cohort, such that a constant predictor would not trivially minimize the prediction error.
2. Sufficient class representation for all categories in classification tasks, such that stable cross-validated performance estimation was feasible.

**Table 1:** Preoperative parameters.

Parameter group	Parameter	Description	Values/level of measurement
Basic parameters	Age [years]	Age at the time of surgery	0–100, metric
	BMI (kg/m^2^)	Body mass index	[0-∞) / metric
Medical history parameters	ICIQ	Total ICIQ1-3	0–35, ordinal
	ICIQ1 (continence)	Continence	0–21, ordinal
	ICIQ2 (quality of life)	Quality of life	0–7, ordinal
	ICIQ3 (overall condition)	Overall condition	0–7, ordinal
	IPSS		0–35, ordinal
	IIEF		0–30, Ordinal
	Previous therapies received	Previous therapies received before surgery?	yes/no, nominal
	Previous therapies	None/radiation/ADT/HIFU	0–3, nominal
	HSI	Hornheider Screening Instrument	0–14, ordinal
	PreOP_ADT	Preoperative ADT treatment	yes/no, nominal
Clinical parameters	suspicious DRE	Digital rectal examination suspicious	yes/no, nominal
	ASA-Score	Perioperative mortality	1–6, ordinal
Laboratory parameters	iPSA [ng/ml]	Initial PSA value	[0-∞) / metric
	Preop.Hct [%]	Haematocrit before surgery	[%] / metric
	Preop.Hb [g/dl]	Hemoglobin before surgery	[g/dl] / metric
Surgical parameters	RARP	Robot-assisted radical prostatectomy	yes/no, nominal
	Nerve preservation	Nerve preservation (yes/no)	yes/no, nominal
	Location of nerve preservation	Right/left/both sides	0–2, nominal
	Nerve preservation _simplified	None/unilateral/bilateral	0–2, nominal
	Highest Gleason	Highest pre-operative Gleason score	6–10, ordinal
	ISUP pre-op	Pre-operative ISUP grade	1–5, Ordinal
	PreOP_cT stage	Preoperative clinical T stage	nominal
	D’Amico-Score	D’Amico-Score	1–3, ordinal
	Preoperative prostate volume	Prostate volume before surgery on mpMRI	[0-∞) / metric

ICIQ, International Consultation on Incontinence Questionnaire^30^; IPSS, International Prostate Symptom Score^31^; IIEF, International Index of Erectile Function^32^; ADT, Androgen Deprivation Therapy; HIFU, High-Intensity Focused Ultrasound; ASA, American Society of Anesthesiologists Physical Status Classification System^33^; ISUP, International Society of Urological Pathology Grade^34^; D'Amico Score, D'Amico risk classification system for prostate cancer^35^.

Outcomes failing to meet these criteria, including several ordinal outcomes with sparsely populated higher-grade categories and continuous outcomes with near-zero variance, were excluded from the main analysis. All applied exclusion criteria are documented in Supplementary Table S2.

### Machine learning and statistical analysis using SHAP

To predict intra- and postoperative outcomes from preoperative data, we implemented a supervised machine learning framework on the parameter sets described (preoperative parameters, Table 1 / intra- and postoperative parameters, Supplementary Table S2). The analysis consisted of data preprocessing, model training and evaluation, and model explanation using SHapley Additive exPlanations (SHAP). Regression (metric scaling) or classification (nominal scaling) was learned depending on the scale level of the respective target parameter. A detailed technical description of all models and mathematical definitions of all metrics is provided in the Supplementary Methods.

### Data preprocessing and feature selection

The dataset consisted of preoperative features (Table 1) used as input to predict the target outcomes (Supplementary Table S2). Prior to model training, a systematic data preprocessing routine was performed. This included the imputation of missing values and the exclusion of features with a high proportion of missing data or multicollinearity to ensure data quality and model stability. The parameters describing nerve-sparing decisions were treated as preoperative input features, as this decision was typically made before surgery. A detailed description of the imputation strategy and the complete list of excluded features is provided in the Supplementary Methods. Several features were excluded from the final analysis to ensure data quality and model stability. First, features with a high proportion of missing values (> 100 instances) were removed, namely: 'ICIQ' (and its subscales), 'IPSS', 'IIEF', and the 'Hornheider Screening Instrument (HSI)'. Second, to avoid multicollinearity, redundant or highly correlated features were excluded. For example, of the two variables describing the extent of nerve preservation (Location of nerve preservation and Nerve preservation simplified), only the simplified version was retained for the analysis. Similarly, binary indicator variables such as 'Previous therapies received' were excluded as their information was contained within more detailed categorical features. Finally, the parameters describing nerve-sparing decisions were treated as preoperative input features, as this decision was typically made before surgery based on tumor characteristics, patient function, and patient preference.

### Machine learning models and evaluation strategy

**Fig. 1:**
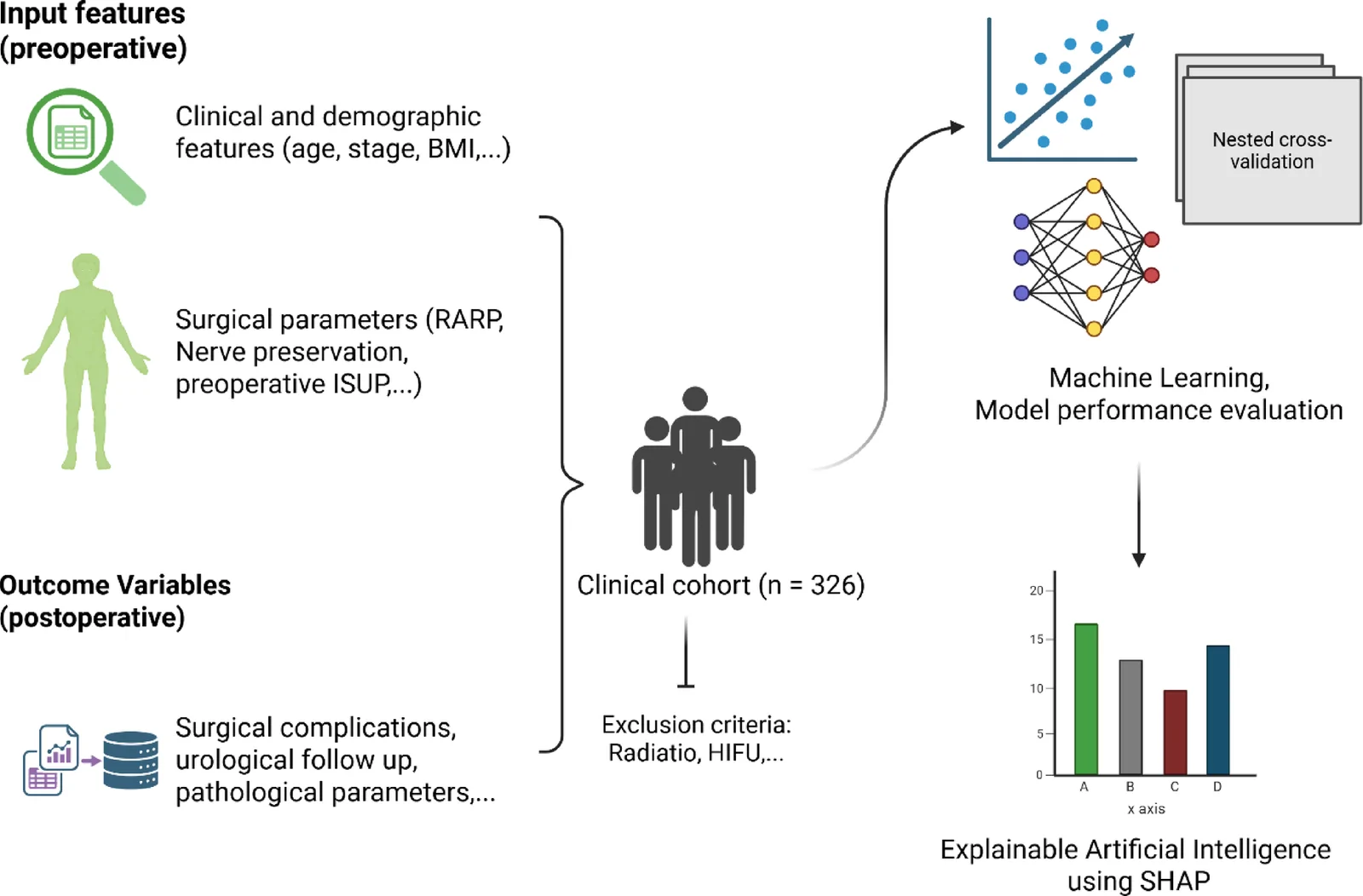
Methodological workflow for the prediction and explanation of postoperative outcomes. The study workflow consisted of three main stages. First (left panel), preoperative data, including clinical and demographic features as well as surgical planning parameters, were collected from a clinical cohort of 326 patients undergoing radical prostatectomy. Intra- and postoperative data were defined as the target outcome variables. Second (top right panel), this dataset was used to develop machine learning (ML) models. A robust nested cross-validation scheme was employed for model training, hyperparameter optimization, and performance evaluation. Third (bottom right panel), the trained ML models were interpreted using an explainable AI (XAI) approach. SHAP (SHapley Additive exPlanations) analysis was applied to the models to quantify the contribution of each input feature, resulting in a ranked list of their predictive importance for each outcome. Created in BioRender. Klein, C. (2025) https://BioRender.com/8t2gae2.

The general procedure is illustrated in Fig. 1. We evaluated four different machine learning algorithms for each predictive task: Random Forest (RF), Gradient Boosting (GB), Support Vector Machine (SVM), and a Neural Network (NN). Given the limited sample size (*n* = 326), we employed a nested cross-validation (CV) scheme to ensure robust hyperparameter tuning and unbiased performance evaluation. The outer loop consisted of a 5-fold CV to estimate the models' generalization performance. For each fold of the outer loop, the remaining data was used as a training set. Within this training set, an inner 5-fold CV was performed for hyperparameter optimization. The hyperparameter configuration that achieved the highest average performance across the inner folds was selected as optimal. This final model configuration was then re-trained on the entire training set and evaluated on the hold-out test set from the outer loop. The final reported performance metrics are the average of the results across the five folds of the outer loop.

### Baseline models

To contextualize the performance of the ML models, we compared them against simple heuristic baseline models. For regression tasks, we used Mean and Median predictors, which consistently predict the mean or median of the target values calculated solely on the training set. For classification tasks, the Mode predictor was used, which predicts the most frequent class identified within the training set. These models make no assumptions about the data and serve as a basis for comparison. In addition to heuristic baselines, we implemented two stronger statistical reference models. First, a clinically obvious single-predictor baseline was constructed using ordinary least squares (OLS) linear regression trained on preoperative haemoglobin alone (for postoperative Hb and Hb Drop) or preoperative haematocrit alone (for postoperative Hct), to assess the incremental gain of the multivariate ML models over the single most clinically plausible predictor. Second, a multivariable Ridge (L2-regularized) linear regression model containing all available preoperative predictors was implemented as a robust clinical-statistical benchmark, evaluated within the same nested 5-fold cross-validation framework. Ridge regression was selected over standard OLS regression to prevent multicollinearity-induced model instability arising from dummy-coded categorical variables.

### Measures of prediction accuracy

Model performance was quantified using standard metrics depending on the task type.

- *For regression tasks*, we used the coefficient of determination (R^2^), Mean Absolute Error (MAE), and Root Mean Squared Error (RMSE).
- *For classification tasks*, we used Accuracy. To provide a more comprehensive assessment of classifier performance, we also calculated the Area Under the Receiver Operating Characteristic Curve (AUC). No explicit class-balancing strategies (e.g., SMOTE or class-weight adjustment) were applied. For FS Decision, the class distribution was near-balanced across all outer folds (FS-positive: 47–57% per fold). For FS Result (the composite three-class utilization/result endpoint), the intermediate class was underrepresented (~ 6–8% per fold), which is acknowledged as a contributing factor to the modest predictive performance for this outcome.

### Explainable artificial intelligence (XAI) using SHAP

To interpret the predictions of the best-performing models and identify the most influential input features, we employed a custom permutation-based Shapley sampling framework, implemented in the spirit of SHapley Additive exPlanations (SHAP). SHAP is a game theory-based method that explains the output of any machine learning model by assigning each feature an importance value for a particular prediction^27,28^. In contrast to conventional SHAP implementations, which typically derive feature attribution directly from model outputs (e.g., predicted values or class probabilities), our framework evaluates feature contributions with respect to model behaviour and predictive performance under the selected evaluation metric. As detailed in the Supplementary Methods (Section 3.2), this custom approach measures the sensitivity of the model's predictive distribution to the progressive removal of feature information via random permutation sampling (R = 2^13^ coalitions). To obtain a global measure of feature importance for each target outcome, we calculated the mean absolute Shapley attribution value for each feature across all patients in the test sets. These values represent the average impact of a feature on the model's predictive performance. For visualization, these absolute attribution values were normalized to sum to 100% for each predictive target, allowing for a direct comparison of the relative importance of each feature. The resulting feature importance rankings should be interpreted as reflecting model-derived predictive associations within this dataset, not as independent clinical effects or causal determinants of outcome.

### Software

All analyses were conducted using Python (Version 3.13). The machine learning models were implemented primarily using the scikit-learn library, and data manipulation was performed with pandas. Visualizations were created using matplotlib. The exact versions of all libraries are documented in the requirements file of the code repository (https://github.com/ChristianRKlein/explainable-ai-radical-prostatectomy).

## Results

### Patient characteristics

A total of 326 patients (men) aged 45 to 83 years were included in the study, with 224 (68.7%) undergoing RARP and 102 (31.3%) undergoing ORP. The baseline clinical and pathological characteristics of the patient cohort are summarized in Table 2. The RARP and open surgery groups were well-matched regarding most preoperative parameters, including BMI, initial PSA, and D'Amico risk classification. However, patients in the RARP group were significantly older and had a different distribution of preoperative ISUP grades (*p* = 0.002 and p = 0.006, respectively). The absence of a significant difference in D'Amico risk classification (*p* = 0.311) despite a significant difference in preoperative ISUP grade distribution (*p* = 0.006) likely reflects the composite nature of the D'Amico score, which integrates ISUP grade with PSA level and clinical T-stage; the absence of significant between-group differences in PSA (*p* = 0.845) may thus partially compensate for the ISUP grade imbalance within the composite score. A comprehensive descriptive statistical analysis of all pre- and postoperative variables is provided in the Supplementary Material (see Supplementary Data 1).

**Table 2:** Baseline patient and tumor characteristics.

Characteristic	All Patients (*n* = 326)	RARP (*n* = 224)	Open (*n* = 102)	*p*-value
Age, median (IQR) [years]	66 (61–70)	67 (62–72)	65 (60–69)	0.002*
BMI, median (IQR) [kg/m ^2^]	26.4 (24–29)	26.5 (24–29)	26.3 (23–31)	0.785
Initial PSA, median (IQR) [ng/mL]	7.7 (5.5–11.8)	7.6 (5.6–11.0)	7.8 (5.4–12.6)	0.845
Preoperative ISUP Grade, *n* (%)				0.006*
1	54 (16.8%)	40 (18.0%)	14 (13.7%)	
2	114 (35.4%)	85 (38.3%)	29 (28.4%)	
3	61 (18.9%)	45 (20.3%)	16 (15.7%)	
4	50 (15.5%)	31 (14.0%)	19 (18.6%)	
5	45 (13.9%)	21 (9.5%)	24 (23.5%)	
D’Amico Risk Classification, *n* (%)				0.311
Low risk	30 (10.0%)	22 (9.8%)	8 (8.8%)	
Intermediate risk	133 (44.2%)	100 (44.6%)	33 (36.3%)	
High risk	138 (45.8%)	102 (45.5%)	50 (54.9%)	
ASA Score, *n* (%)				0.703
1	48 (15.1%)	31 (14.2%)	17 (17.7%)	
2	205 (64.5%)	145 (66.5%)	60 (62.5%)	
3	65 (20.4%)	42 (19.3%)	19 (19.8%)	
Nerve Sparing (any), *n* (%)	187 (57.7%)	127 (56.9%)	58 (57.4%)	0.132

The table summarizes the preoperative characteristics of the entire patient cohort (*n* = 326) and provides a comparison between the robot-assisted radical prostatectomy (RARP) and open radical prostatectomy (ORP) groups. Continuous variables are presented as median with interquartile range (IQR). Categorical variables are presented as counts with percentages, *n* (%). *P*-values were calculated to assess significant differences between the RARP and ORP groups, using the Mann–Whitney U test for continuous variables and the Chi-squared test (or Fisher's exact test where appropriate) for categorical variables. Statistically significant *p*-values (*p* < 0.05) are marked with an asterisk (*). Abbreviations: ASA, American Society of Anesthesiologists Physical Status Classification; BMI, body mass index; IQR, interquartile range; ISUP, International Society of Urological Pathology; ORP, open radical prostatectomy; PSA, prostate-specific antigen; RARP, robot-assisted radical prostatectomy.

### Predictive performance of machine learning models

**Fig. 2:**
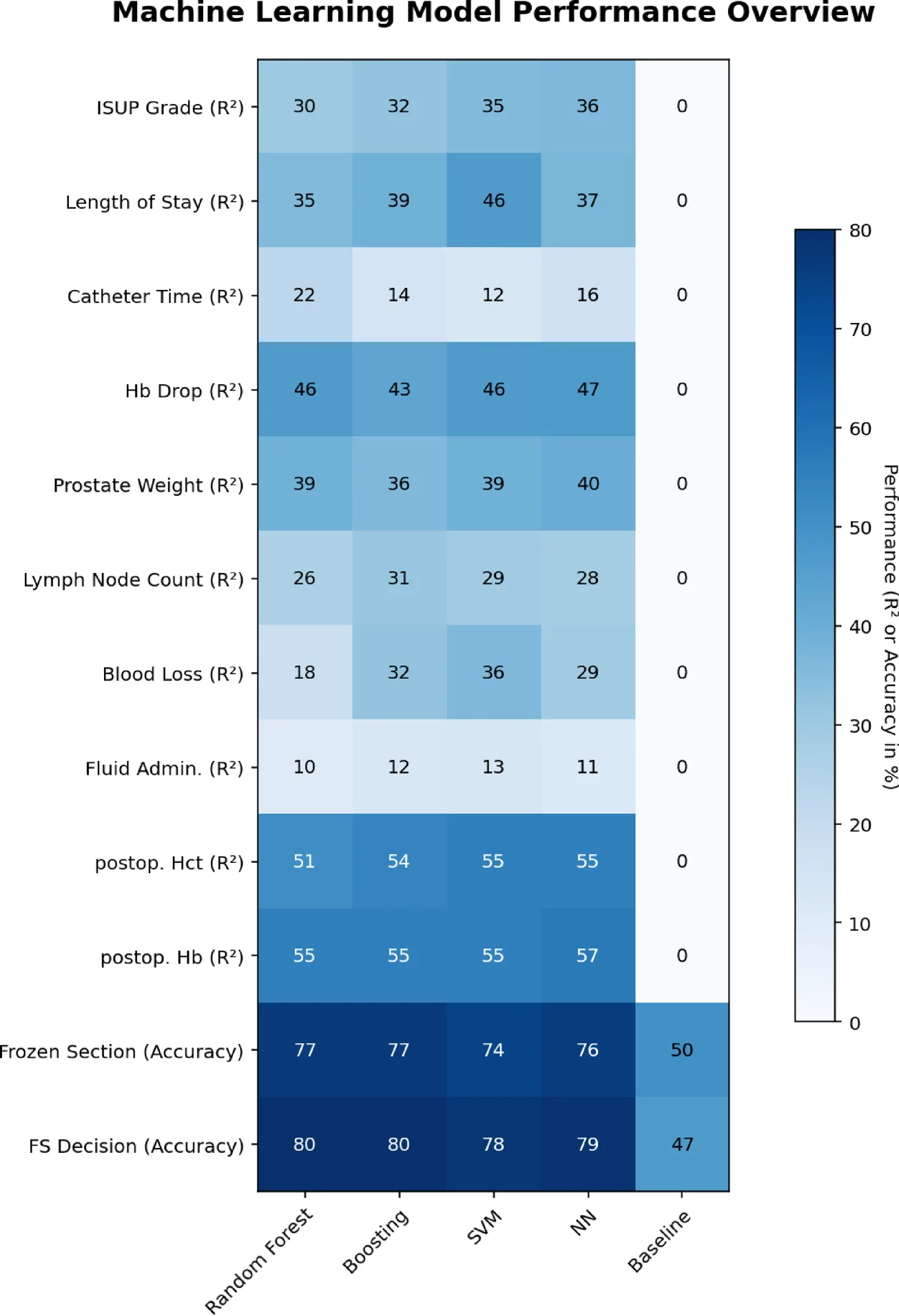
Overview of machine learning model performance across all predictive tasks. The heatmap summarizes the predictive performance of four machine learning models (Random Forest, Boosting, Support Vector Machine [SVM], and Neural Network [NN]) and a heuristic baseline model across twelve distinct postoperative outcomes. Each cell displays the primary performance metric, with darker shades of blue indicating better predictive performance. All metrics were derived from the final results of a 5-fold cross-validation scheme. For regression tasks (indicated by R^2^ in the y-axis label), the performance is measured by the coefficient of determination (R^2^), representing the proportion of variance in the target variable explained by the model. For classification tasks (indicated by Accuracy in the y-axis label), the performance is measured by Accuracy, representing the proportion of correctly classified instances. The baseline model for regression tasks is the mean predictor (R^2^ ≈ 0), and for classification tasks, it is the mode predictor. “Frozen Section” denotes the composite FS Result endpoint combining utilization and pathological result categories. Abbreviations: CV, Cross-Validation; FS, Frozen Section; Hb, Hemoglobin; Hct, Hematocrit; ISUP, International Society of Urological Pathology; NN, Neural Network; R^2^, Coefficient of Determination; SVM, Support Vector Machine.

**Fig. 3:**
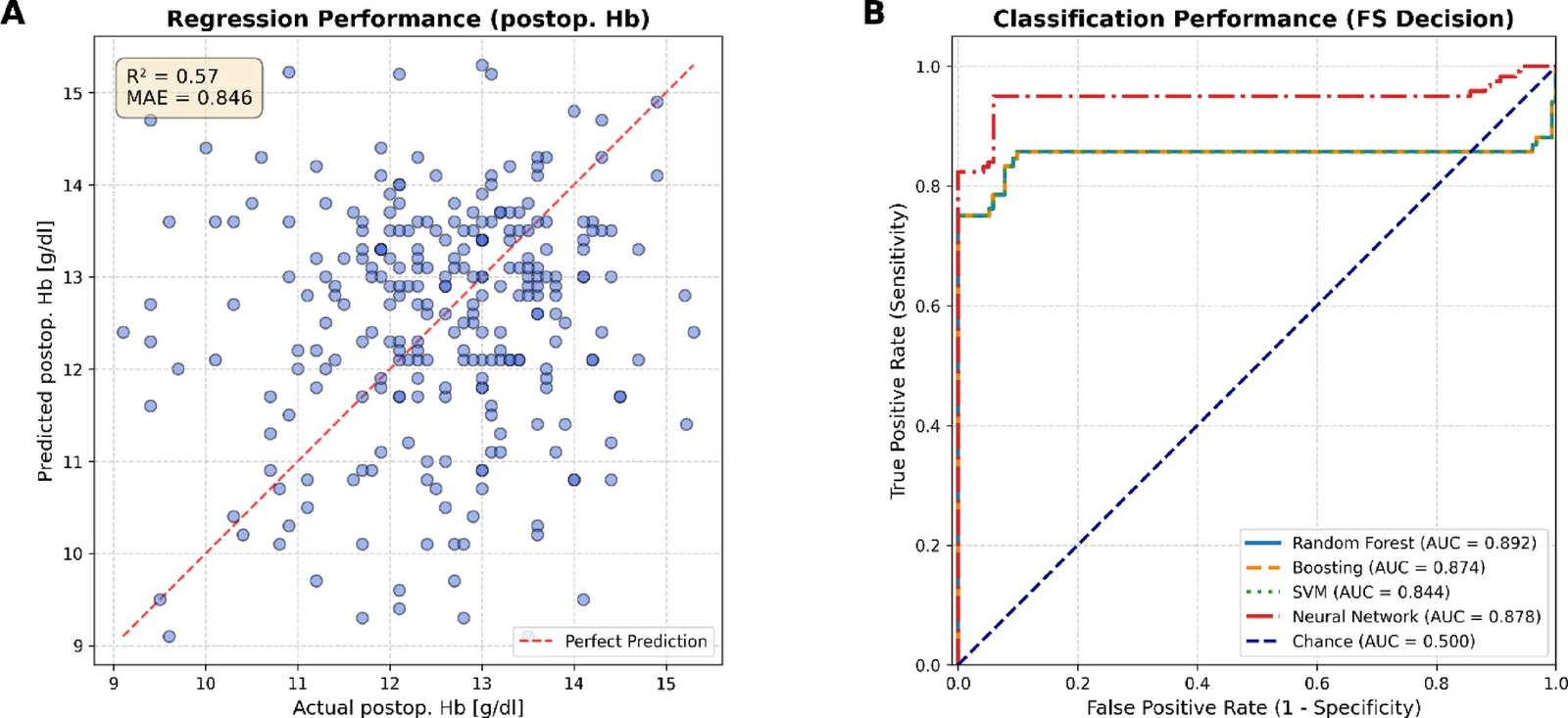
Exemplary visualization of model performance for regression and classification tasks. (**A**) Scatter plot of the predicted versus actual postoperative hemoglobin (postop. Hb) values for the best-performing regression model (Neural Network, NN). Each point represents a patient from the combined 5-fold cross-validation test sets. The red dashed line indicates perfect prediction (y = x). The inset box displays the overall coefficient of determination (R^2^) and the Mean Absolute Error (MAE) for the model. (**B**) Comparative Receiver Operating Characteristic (ROC) curves for the four machine learning models predicting the binary decision to perform an intraoperative frozen section (FS Decision). The dashed diagonal line represents the performance of a random classifier. The Random Forest achieved an AUC of 89.2 ± 2.9%, Gradient Boosting 87.4 ± 4.4%, Support Vector Machine 84.4 ± 5.9%, and the Neural Network 87.8 ± 2.8%, demonstrating robust and consistent discriminative performance across all models and folds. Abbreviations: AUC, Area Under the Curve; CV, Cross-Validation; FS, Frozen Section; Hb, Hemoglobin; MAE, Mean Absolute Error; NN, Neural Network; R^2^, Coefficient of Determination; ROC, Receiver Operating Characteristic.

To assess the feasibility of predicting postoperative outcomes from preoperative clinical data, we evaluated four distinct ML models against simple heuristic baselines. A comprehensive overview of the model performance across all twelve regression and classification tasks is presented in the heatmap in Fig. 2. The results indicate that the ML models consistently outperformed the baseline predictors. The predictive power, however, varied considerably depending on the target parameter. The highest predictive performance for regression tasks was achieved for postoperative hematological parameters, particularly postoperative hemoglobin (postop. Hb), with a coefficient of determination (R^2^) reaching up to 0.57 for the Neural Network (NN) model. Strong performance was also observed for predicting the length of hospital stay (R^2^ up to 0.46) and the postoperative ISUP grade (R^2^ up to 0.37). In contrast, the models showed limited success in predicting parameters such as intraoperative fluid administration and catheter dwell time, with R^2^ values generally below 0.20. For classification tasks, all ML models demonstrated robust performance in predicting the decision to perform a frozen section (FS Decision), achieving accuracies between 63 and 67%, well above the 47% accuracy of the baseline mode predictor. For the prediction of the combined FS utilization/result category (FS Result, a composite three-class variable encoding whether frozen section was performed and, where applicable, its pathological result), the models achieved accuracies of up to 62%. While modest in absolute terms, this represents a substantial improvement over the 50% accuracy of the baseline model, demonstrating that preoperative data contains a learnable signal regarding this composite endpoint. A detailed breakdown of all performance metrics, including Mean Absolute Error (MAE), Root Mean Squared Error (RMSE), R^2^, and Accuracy for all models, is provided in Supplementary Table S1. To illustrate these findings in more detail, we visualized the performance for the best-performing regression and classification tasks (Fig. 3). The scatter plot for the NN model's prediction of postoperative Hb (Fig. 3A) shows a clear positive correlation between the predicted and actual values (R^2^ = 0.57, MAE = 0.846), confirming the model's strong predictive capability. For the classification task of predicting the FS Decision (Fig. 3B), the Receiver Operating Characteristic (ROC) curves demonstrate excellent discriminative ability for all models. The Random Forest achieved an AUC of 89.2 ± 2.9%, Gradient Boosting 87.4 ± 4.4%, Support Vector Machine 84.4 ± 5.9%, and the Neural Network 87.8 ± 2.8%, demonstrating robust and consistent discriminative performance across all models and folds. Fold-level AUC values for all models are provided in Supplementary Table S1.

### Explainability and identification of key predictive factors using SHAP

**Fig. 4:**
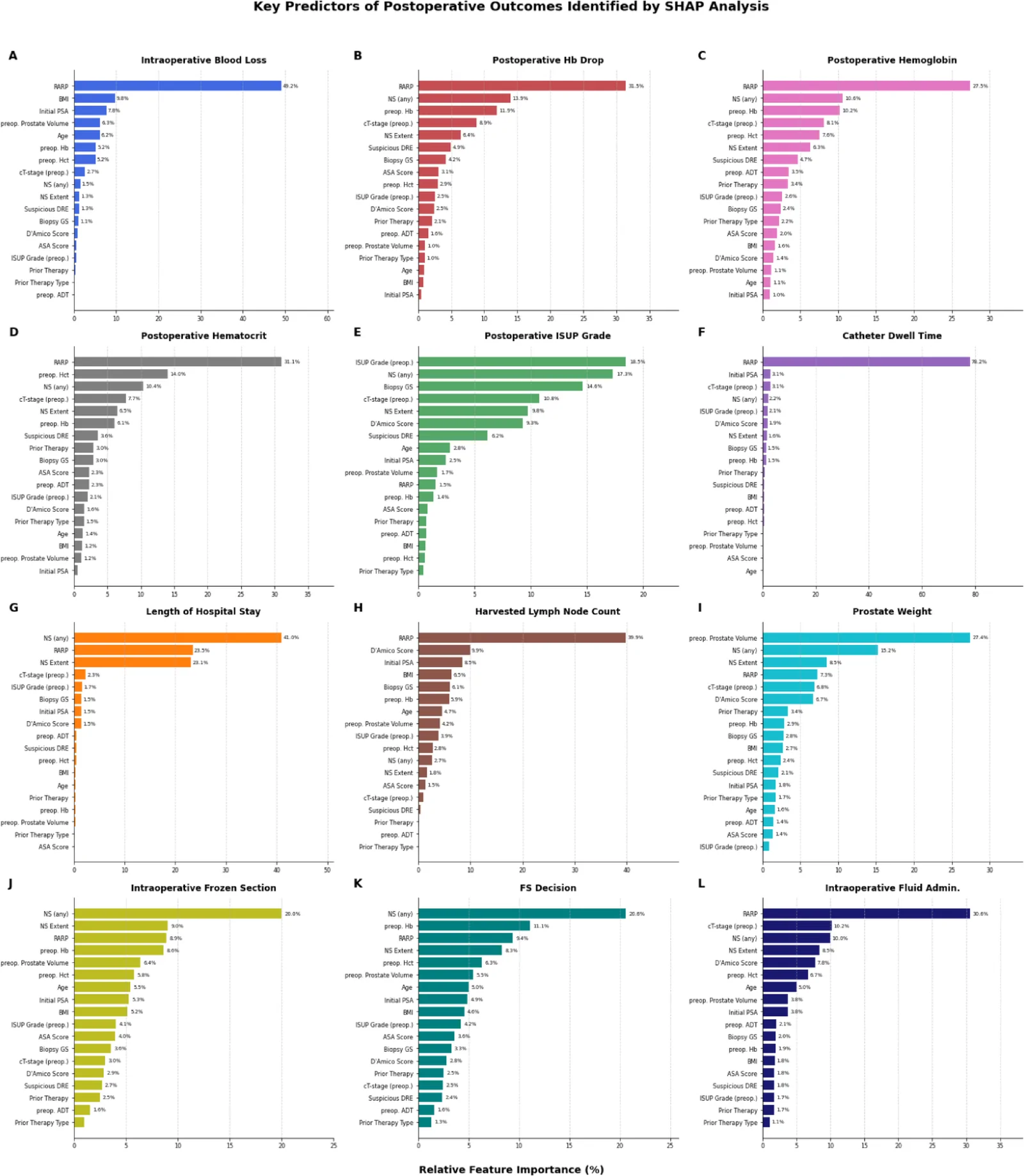
Relative importance of preoperative parameters for predicting twelve distinct postoperative outcomes, as determined by SHAP analysis. The figure displays the results of the custom Shapley-based feature importance analysis for twelve predictable target parameters. Feature importance values reflect model-derived predictive associations within this dataset and should not be interpreted as independent clinical effects. Each panel shows a horizontal bar chart where the length of the bar represents the relative importance of each preoperative feature in the prediction model. The analysis reveals distinct patterns of feature importance, with some outcomes being predominantly driven by surgical-procedural factors (e.g., RARP, NS), while others are more influenced by preoperative patient and tumor characteristics (e.g., preoperative ISUP grade, PSA). For each panel, feature importance was quantified by the mean absolute SHAP value, which was then normalized to a relative importance scale summing to 100% to facilitate comparison across outcomes. The individual panels display the feature importance for the prediction of: (**A**) Intraoperative Blood Loss (**B**) Postoperative Hemoglobin Drop (**C**) Postoperative Hemoglobin (**D**) Postoperative Hematocrit (**E**) Postoperative ISUP Grade (**F**) Catheter Dwell Time (**G**) Length of Hospital Stay (**H**) Harvested Lymph Node Count (**I**) Prostate Weight (**J**) Intraoperative Frozen Section (Combined FS utilization/result category) (**K**) The decision to perform a Frozen Section (FS Decision) (**L**) Intraoperative Fluid Administration Abbreviations: ADT, androgen deprivation therapy; ASA, American Society of Anesthesiologists Physical Status Classification; BMI, body mass index; DRE, digital rectal examination; FS, frozen section; GS, Gleason Score; Hb, hemoglobin; Hct, hematocrit; ISUP, International Society of Urological Pathology; NS, nerve sparing; preop., preoperative; PSA, prostate-specific antigen; RARP, robot-assisted radical prostatectomy; SHAP, SHapley Additive exPlanations.

To understand the basis of the models' predictions and identify the most influential preoperative parameters, we conducted a SHAP analysis. Figure 4 summarizes the relative importance of all input features for the prediction of the twelve target outcomes, revealing distinct patterns of clinical drivers for different endpoints. The analysis highlighted a clear distinction between outcomes driven by surgical-procedural factors and those determined by patient- and tumor-specific characteristics. The prediction of Catheter Dwell Time (Fig. 4F), for example, was overwhelmingly dominated by the surgical approach (RARP), which accounted for over 78% of the predictive importance. For the Length of Hospital Stay (Fig. 4G), the SHAP analysis again revealed a pattern of procedural dominance. The RARP approach was a major predictor for a shorter stay. Notably, nerve-sparing decisions were also identified as a highly influential factor. While not directly causal, this statistical association likely reflects that patients eligible for nerve sparing represent a lower-risk cohort predisposed to an uncomplicated postoperative course. In contrast, traditional patient risk factors such as age and BMI were of marginal relevance for this outcome. Procedural factors also played a primary role in predicting perioperative physiological changes. For Intraoperative Blood Loss (Fig. 4A), the surgical approach was the single most important predictor (49.2%). Specifically, the model learned that the use of RARP was strongly associated with a prediction of lower blood loss compared to open surgery. For the postoperative Hb Drop (Fig. 4B), RARP remained the leading factor associated with a prediction of a smaller drop (31.5%). Interestingly, this outcome was also strongly influenced by the decision to perform nerve sparing, which, in our cohort, was associated with a prediction of a smaller Hb drop as well, and the preoperative Hb value, indicating a combined influence of surgical technique and patient baseline status. The prediction of the final Postoperative Hemoglobin (Fig. 4C) and Hematocrit (Fig. 4D) was correspondingly driven by RARP and the respective preoperative baseline values. The model's reliance on the surgical method for these absolute values reflects its learning of the strong association between RARP and a smaller postoperative hemoglobin drop. Likewise, the model for Intraoperative Fluid Administration (Fig. 4L) learned a clinically plausible pattern. The RARP approach was the most important predictor (30.6% importance), being associated with a prediction of lower fluid volumes compared to open surgery. This was followed by risk indicators such as the preoperative cT-stage, where a more advanced stage was associated with a prediction of higher fluid administration. Interestingly, nerve-sparing decisions also held significant predictive value. Specifically, within the RARP group, procedures with nerve sparing were associated with lower median fluid volumes than those without. Although this difference was not observed in the open surgery subgroup where the median was identical, the model learned this overall trend as a predictive feature (see Supplementary Data for detailed statistical analysis). This composite pattern suggests that fluid administration is guided by both the standard protocols of the surgical method and the anticipated complexity of the individual case, which is informed by tumor stage and the feasibility of nerve preservation. The Postoperative ISUP Grade (Fig. 4E) was, as expected, best predicted by the preoperative ISUP grade and biopsy Gleason score, validating the model's ability to learn clinically established correlations. The prediction of the final Prostate Weight (Fig. 4I) was most strongly determined by the preoperative prostate volume measured by MRI (27.5%), which serves as a strong validation of the model's learning process. Finally, the SHAP analysis provided insights into the drivers of intraoperative procedures. The prediction of the combined FS utilization/result category and the separate FS Decision endpoint (Fig. 4J, 4K) were most directly influenced by parameters representing the preoperative surgical plan, namely nerve-sparing decisions and the RARP approach. While these planning parameters are themselves informed by oncological risk factors (e.g., preoperative ISUP grade, cT-stage), their high ranking in the SHAP analysis—above the individual risk factors themselves—suggests that the model identified the surgical plan as the most powerful proxy for the complex set of conditions that ultimately lead to a frozen section. This indicates that the decision is not a simple function of a single risk score, but a multifactorial assessment for which the overall surgical strategy serves as the best summary predictor. The number of Harvested Lymph Nodes **(**Fig. 4H**)** was unique in that the model learned it to be a function of both the surgical method (RARP, 39.9%) and the patient's oncological risk profile, with the D'Amico risk score and initial PSA being the next most important predictors.

## Discussion

In this proof-of-concept study, we demonstrated that machine learning models can predict a range of critical postoperative outcomes in radical prostatectomy using routinely collected preoperative data. This work shows that AI can leverage standard clinical parameters for enhanced preoperative planning. While many of the identified predictive patterns serve as a quantitative validation of established clinical knowledge, the use of SHAP for model explanation allowed us to deconstruct the “black box” of these models and to disentangle the predictive influence of surgical-procedural factors from patient- and tumor-specific characteristics. Given the mixed cohort structure, including known differences between the RARP and ORP groups (see Supplementary Data 1), some dominant predictors, particularly surgical approach, likely reflect established between-group differences captured by the models rather than entirely novel relationships. Accordingly, the present findings should be interpreted primarily as confirmatory and hypothesis-generating within this proof-of-concept framework. Furthermore, the observed baseline differences between the RARP and ORP groups, including patient age and preoperative ISUP grade distribution, mean that surgical approach also serves as a proxy for patient characteristics in the pooled analysis, further complicating the direct causal interpretation of SHAP-derived feature importances. The stratified subgroup analyses presented in the Supplementary Material partially address this limitation by allowing feature importance patterns to be assessed within more homogeneous subpopulations.

### Discussion of clinical findings and implications

A key strength of the SHAP analysis is its ability to reveal the underlying structure of the predictive tasks. Our results clearly stratified the target outcomes into two distinct categories: those driven by standardized clinical protocols and those determined by intrinsic patient or tumor biology. First, we identified outcomes that are primarily determined by procedural protocols rather than intrinsic patient risk. The prediction of Catheter Dwell Time was overwhelmingly dominated by the surgical approach (RARP vs. open), which accounted for over 78% of the predictive importance. We acknowledge that this finding is largely structural: surgical approach is itself an input feature, and these outcomes are governed by approach-specific protocols. This pattern therefore reflects known protocol-driven differences between RARP and ORP rather than novel patient-specific predictors. To separate structural from patient-specific signals, we conducted stratified SHAP analyses within the RARP and ORP subgroups separately; the results are presented in the Supplementary Material and reveal more nuanced within-arm predictive patterns, particularly within the larger RARP subgroup. Despite the combined predictive importance of over 87% for nerve-sparing decisions and the RARP approach in predicting the length of hospital stay, this strong association should be interpreted as a statistical correlation reflecting standardized clinical protocols within our dataset, rather than a direct causal link. This insight opens an avenue for data-driven optimization and standardization of these clinical pathways. The prediction of Intraoperative Blood Loss was also primarily driven by the RARP approach, a finding consistent with existing literature, which our analysis now quantifies in relation to other factors. Second, our results highlight a clear opportunity for preoperative patient optimization through the identification of modifiable risk factors. The models for predicting postoperative hematological changes (postop. Hb, Hct, and Hb Drop) consistently identified the preoperative Hb value as a highly influential factor. SHAP analysis identified preoperative hemoglobin as a stronger predictive feature for postoperative Hb Drop than non-modifiable factors such as age within this dataset. This association is consistent with the clinical plausibility of preoperative anemia as a risk factor, and future prospective studies should evaluate whether targeted preoperative optimization of hemoglobin levels translates into improved postoperative hematological outcomes. The observed association between preoperative hemoglobin and postoperative hematological outcomes suggests that ML-based risk stratification could potentially complement Patient Blood Management programs; however, prospective validation in independent cohorts will be required before clinical integration can be considered. Third, our approach both validated established clinical knowledge and uncovered novel associations. The prediction of postoperative ISUP Grade was dominated by preoperative ISUP grade and biopsy Gleason score. We explicitly acknowledge that this finding largely reflects the well-established and expected correlation between pre- and postoperative pathological grading, preoperative and postoperative ISUP are by definition highly correlated variables, rather than a novel clinical insight. This result should therefore be interpreted primarily as a methodological validation of the model's internal consistency, confirming that the framework correctly identifies known relationships, rather than as an independently informative predictive finding. While preoperative ISUP grade was confirmed as the single most important predictor (18.5% importance), our model's strong overall performance (R^2^ = 0.37) demonstrates that this prediction is substantially 'boosted' by the inclusion of other parameters. Notably, the planned surgical approach regarding NS was identified as a predictor of almost equal importance (17.3%) to the preoperative ISUP grade itself. Crucially, the high importance of the nerve-sparing decision must be interpreted strictly as a correlational finding. It does not imply that the surgical act of nerve sparing influences the tumor biology. Instead, the model leverages the surgeon's implicit risk stratification embedded in the surgical plan, using this decision as a powerful proxy variable for a favorable underlying disease status. The preoperative decision to perform nerve sparing is itself based on a complex assessment of oncological risk, integrating factors like tumor stage, biopsy results, and MRI findings. By learning this association, the model leverages the surgeon's implicit risk stratification embedded in the surgical plan. This highlights the power of a multiparametric AI model to integrate not only direct tumor characteristics but also the expert clinical decisions derived from them for an improved prediction. The prediction of Prostate Weight was most dependent on the preoperative prostate volume. Furthermore, our analysis consistently showed a low SHAP value for a suspicious digital rectal examination (DRE) across multiple outcomes, providing quantitative support for its de-emphasis in recent clinical guidelines^36,37^. The prediction of the Harvested Lymph Node Count perfectly illustrated how our models learned to mirror clinical reality, identifying it as a function of both the surgical approach (RARP) and the established oncological risk (D'Amico Score, PSA). Finally, the SHAP analysis provided distinct insights into the prediction of two related intraoperative events: the decision to perform frozen section (FS Decision) and the combined utilization/pathological result category (FS Result). For the FS decision, the model's prediction was driven by a multifactorial assessment, primarily influenced by the surgical plan (NS, RARP) and the patient's baseline status (preop. Hb), rather than a single oncological risk factor. This remarkable insight suggests that the complex clinical judgment leading to the request for a frozen section is largely captured by these preoperative parameters. For the composite FS Result endpoint, the predictive pattern was similar, with nerve-sparing decisions and the RARP approach being the most influential features. This highlights that the same factors that drive the decision to perform a frozen section are also the strongest predictors of this composite utilization/result category. This could have profound implications for clinical practice. The observed predictive associations for the composite FS Result endpoint suggest that preoperative features may carry informative signal regarding frozen-section utilization and, where performed, the pathological result. Future prospective studies should evaluate whether such preoperative risk stratification could inform surgical planning, pending external validation and formal decision-analytic evaluation. The potential harms of misclassification, including patients being denied nerve sparing or being directed toward alternative therapies on the basis of an incorrect prediction, must be carefully considered in any future clinical translation.

### Discussion of the method

This study demonstrates that even with a relatively small dataset for machine learning standards (*n* = 326), robust and clinically plausible predictive models can be developed. Our heatmap analysis (Fig. 2) showed that the implemented machine learning models provided a significant performance uplift compared to heuristic baseline predictions, indicating that the models captured informative signal within the dataset. However, these baseline comparisons represent only a minimal reference point and should not be interpreted as proof of superiority over conventional statistical approaches. In a cohort with pronounced group-level structure, part of this performance gain may reflect the capture of obvious between-group differences rather than the discovery of novel clinically meaningful predictors. To further contextualise the added value of the ML approach, we additionally compared model performance against a clinically obvious single-predictor baseline (preoperative haemoglobin predicting postoperative haemoglobin) and a multivariable Ridge regression model using all available preoperative features. The single-predictor baseline achieved R^2^ values of approximately 26% for postoperative haemoglobin, substantially below the ML models (R^2^ up to 57.5%), confirming that the multivariate ML framework provides meaningful incremental gain beyond the single most clinically plausible predictor. The multivariable Ridge regression baseline performed comparably to the ML models for several outcomes, highlighting that much of the predictive signal in this dataset is also accessible to simpler linear models. This finding is consistent with the proof-of-concept nature of the present study and does not diminish the value of the explainability framework, which provides interpretable feature importance rankings irrespective of the marginal performance gain over linear baselines. A noteworthy finding from our comparative analysis was the performance pattern on the classification task (Fig. 2B). All four models demonstrated robust and consistent performance for FS Decision prediction, with AUC values ranging from 84.4% to 89.2% and low variability across outer folds, indicating a stable and learnable signal in the preoperative feature set. The close agreement between model architectures suggests that the predictive signal for this outcome is strong and clearly structured, rather than dependent on complex non-linear relationships accessible only to more flexible models. Beyond predictive performance, the core methodological strength of this work lies in the application of SHAP. This technique answers a critical clinical question: what is the quantitative influence of a single input parameter on the prediction, while systematically accounting for all other parameters? By ranking features based on their SHAP values, we moved beyond mere prediction to an interpretable, explainable AI (XAI) framework. This approach unlocks several innovative avenues for future research. First, our observation that for many outcomes, the predictive feature space is sparse, suggests the potential for SHAP-based compression of the preoperative parameter space^38^. In the present study, SHAP was applied exclusively as a post hoc interpretability framework, with all models trained on the full preoperative feature set without prior SHAP-based filtering. Future work could explore whether parsimonious models trained on SHAP-selected feature subsets retain comparable predictive performance, which would further support the clinical utility of the explainability framework. Second, the additive nature of Shapley values could be leveraged to create a novel, SHAP-based score for intervention risk. By summing the individual SHAP values of a patient's risk factors, a highly personalized and transparent risk estimate could be generated, a concept that warrants prospective evaluation.

### Discussion of related work

Our study is situated at the intersection of urological surgery and computational medicine, contributing to the rapidly evolving field of artificial intelligence in prostate cancer management. The application of AI in this domain has seen exponential growth, as evidenced by recent bibliometric analyses and systematic reviews^17–19^. To date, research has predominantly focused on two major areas: the automated diagnosis and grading of prostate cancer from histopathological whole-slide images, exemplified by landmark studies like the PANDA challenge^13^, and the detection of tumors on medical imaging, particularly MRI^39,40^. These studies have powerfully demonstrated the potential of AI in diagnostic tasks. Beyond diagnostic tasks, AI and radiomics-based approaches have also been applied to predict oncological outcomes and treatment response in prostate cancer^41,42^, providing important context for the present study's focus on preoperative prediction of surgical outcomes. However, the prediction of a broad spectrum of postoperative clinical and procedural outcomes based on routine preoperative data remains less explored. A key contribution of our work is the application of an explainable AI (XAI) framework to move beyond simple prediction. While some studies have successfully developed predictive models for individual outcomes like length of stay^43^ or urethrovesical anastomotic leakage^44^, these models are often treated as “black boxes,” limiting their clinical translatability. To our knowledge, our study is the first to apply a systematic, explainable framework like SHAP^27^ across a comprehensive suite of twelve distinct postoperative endpoints in radical prostatectomy. By doing so, we not only predict an outcome but also provide a quantitative ranking of the factors driving that prediction, a critical step towards building trust and facilitating clinical adoption. Our findings regarding the impact of the surgical approach are well-aligned with, and add a new dimension to, the extensive body of literature comparing RARP and ORP. Our SHAP analysis identified RARP as a dominant predictor for reduced intraoperative blood loss, shorter catheter dwell times, and shorter hospital stays. This is consistent with large-scale meta-analyses and randomized controlled trials that have reported favorable perioperative outcomes for RARP^5,45,46^. Within this cohort, our analysis provides a quantification of the relative predictive importance of surgical approach compared to other preoperative features. For example, surgical method accounted for nearly half of the predictive importance for blood loss and over 78% for catheter dwell time within our dataset, outweighing all patient-specific factors combined in these models. These associations are consistent with the existing literature but should not be interpreted as causal effect estimates or as generalizable beyond the present cohort. Furthermore, our results on hematological parameters contribute to the important discussion on perioperative blood management^47^. The SHAP analysis identified preoperative hemoglobin as a key modifiable risk factor for postoperative Hb drop. This highlights the clinical importance of preoperative patient optimization, a field that has been shown to improve outcomes such as urinary continence^48^. While studies like Han et al. have investigated the oncological safety of perioperative blood transfusions, finding no influence on 5-year biochemical recurrence^49^, our work focuses on the preceding step: By using explainable AI to preoperatively identify patients at high risk for a significant hemoglobin drop—a risk influenced by both surgical approach and patient baseline status (see Supplementary Data 1 for descriptive analysis)—our approach provides a tool for targeted optimization strategies within a modern patient blood management framework, aiming to improve postoperative recovery and minimize hematological morbidity altogether.

### Limitations

Our study has several limitations. First, as a single-center study, our models are inherently trained on our institution's specific patient population and clinical protocols. Furthermore, no calibration analysis or decision curve analysis was performed for the classification outcomes. Calibration plots and Brier scores would be required before any classification model could be considered for clinical decision support; these analyses are deferred to future prospective validation studies with larger, independently collected cohorts. The generalizability of these models requires external validation in multi-center cohorts. Second, the retrospective design carries a potential risk of unmeasured confounding variables. Third, while our sample size was sufficient for this proof-of-concept study, larger datasets could yield even more robust and nuanced models. A key methodological consideration is our treatment of nerve-sparing decisions as a preoperative input feature. At our institution, this decision is made preoperatively based on tumor characteristics, erectile function, and patient preference; intraoperative deviation from the planned approach represents the clinical exception. Although the final decision to perform nerve sparing is made intraoperatively, it is largely based on a preoperative plan. However, this approach does not account for intraoperative changes to that plan. Consequently, the proportion of cases in which the preoperatively planned nerve-sparing approach was modified intraoperatively cannot be reported; any such cases would introduce a degree of label leakage from the intraoperative to the preoperative domain, which should be considered when interpreting the nerve-sparing-related SHAP findings. Future studies could address this by modeling the nerve-sparing decision itself as a primary outcome, which would allow for the identification of its key preoperative drivers. Finally, it must be emphasized that the explainability framework used in this study reflects associations learned by the predictive models and their contribution to model performance within the studied dataset, rather than direct decompositions of individual clinical predictions or independent biological determinants of outcome. These associations represent correlations and should not be interpreted as causal clinical effects. In addition, our SHAP analysis relied on an approximation framework in which excluded features within sampled coalitions were assigned a fixed placeholder value. While computationally practical, this approach may generate feature combinations outside the empirical training distribution; therefore, the resulting attribution values should be interpreted as approximate explanations of model behavior rather than physiologically realistic simulations or causal clinical effect estimates. We also frankly report that some outcomes, particularly surgical complications and long-term functional follow-up, were not well-predicted, likely due to the low frequency of events (class imbalance) in our cohort. Similarly, the prediction of ordinal outcomes, such as the full 5-point Clavien-Dindo score, proved challenging. Although both regression (with subsequent rounding) and classification approaches were explored, the models did not achieve performance significantly better than baseline predictors, likely due to the sparse population of instances in the higher-grade categories. Therefore, these outcomes were not included in the main analysis. Given the conceptual challenge of using a physician's decision as an input feature, a sensitivity analysis excluding all nerve-sparing-related input features was conducted; the results are presented in the Supplementary Material. A further limitation concerns the substantial baseline heterogeneity between the RARP and ORP groups, including differences in sample size (224 vs. 102), patient age, preoperative ISUP grade distribution, and surgical margin rates. These likely reflect systematic patient selection patterns, and the SHAP-derived feature importances in the pooled analysis may therefore partly capture selection differences rather than independent procedure-driven signals. Propensity score matching was considered but deemed not feasible given the limited total sample size. Formal statistical model comparison tests (e.g., DeLong test for AUC; paired Wilcoxon test across folds for R^2^) were not performed, and no formal multiplicity correction was applied across the 48 model-outcome combinations evaluated; model performance is therefore compared descriptively using mean ± SD across outer folds. Future studies with larger sample sizes should consider formal model comparison frameworks. The stratified subgroup analyses presented in the Supplementary Material represent a pragmatic alternative; future studies in larger, prospectively collected cohorts will be required to fully disentangle procedural effects from selection bias.

## Conclusion and outlook

In conclusion, this study demonstrates that the integration of machine learning with explainability methods like SHAP is a powerful tool for dissecting the complex interplay of factors that determine postoperative outcomes in radical prostatectomy. Our work provides a methodological proof-of-concept demonstrating how explainable machine learning can be applied to routine clinical data to generate transparent predictive insights and clinically testable hypotheses in surgical oncology. However, as a single-centre retrospective analysis without external validation, the findings should be interpreted as hypothesis-generating rather than as immediately practice-changing clinical evidence. In particular, predictive associations identified through SHAP-based feature importance, such as the role of preoperative haemoglobin levels in postoperative haematological outcomes, represent correlations observed within this dataset and cannot be interpreted as evidence-based clinical guidance without prospective validation. A further interesting direction for future work could be the direct computation of Shapley values for metric target parameters to quantify the statistical interdependencies between pre- and postoperative variables without an intermediate ML model. Future validation in larger and more homogeneous cohorts will be required before translation into clinical decision support applications can be considered. We emphasize that the findings of this single-centre retrospective proof-of-concept study should not be used to guide clinical decision-making without prospective validation in independent, larger, and more homogeneous cohorts.

## Supplementary Information

Below is the link to the electronic supplementary material.

Supplementary Material 1

Supplementary Material 2

## Data Availability

The aggregated data supporting the findings of this study are available within the article and its Supplementary Information files. Specifically, the comprehensive descriptive statistical analysis is provided in the Supplementary Information, the detailed model performance metrics are available in Supplementary Table S1, and the raw, unnormalized mean absolute SHAP values used to generate the feature importance plots are provided in Supplementary Table S3. The individual patient-level data are not publicly available due to privacy restrictions protecting patient health information. However, de-identified data may be made available by the corresponding author upon reasonable request and contingent upon the execution of a data use agreement. The complete analysis code is publicly available at: https://github.com/ChristianRKlein/explainable-ai-radical-prostatectomy.

## Abbreviations

ADT: Androgen deprivation therapy
ASA: American Society of Anesthesiologists Physical Status Classification System
BMI: Body mass index
CD: Clavien-Dindo classification
DRE: Digital rectal examination
EK: Erythrocyte concentrate
HIFU: High-intensity focused ultrasound
ICIQ: International consultation on incontinence questionnaire
IIEF: International index of erectile function
IPSS: International prostate symptom score
ISUP: International Society of Urological Pathology Score
MAPE: Mean absolute percentage error
ML: Machine learning
NVB: Neurovascular bundle
ORP: Open radical prostatectomy
PSA: Prostate-specific antigen
RARP: Robot-assisted radical prostatectomy
RF: Random forest
RMSE: Root mean squared error
SHAP: SHapley Additive exPlanations
SVM: Support vector machine
SV: Shapley value
