# Supplementary material for: Explainable artificial intelligence reveals key surgical parameters in robot-assisted and open radical prostatectomy
Source: Sci Rep. 2026 Aug 26;16:26797. doi: 10.1038/s41598-026-68074-9 (PMC13507051; doi:10.1038/s41598-026-68074-9)
Supplement: Supplementary file 1 [file 41598_2026_68074_MOESM1_ESM.pdf]

Supplementary Material for:

Explainable Artificial Intelligence reveals key surgical parameters in robot-assisted and open radical prostatectomy

Christian R. Klein[1,5*], Pia Heuser[3,5], Elena Trunz[2], Glen Kristiansen[4,5], Peter Brossart[1,5], Manuel Ritter[3,5], and Philipp Krausewitz[3,5]

[1]Clinic of Internal Medicine III, Oncology, Hematology, Immune-Oncology and Rheumatology, University Hospital, Bonn, Bonn, Germany
[2]Institute for Computer Science, Department of Visual Computing, University of Bonn, Bonn, Germany
[3]Department of Urology, University Hospital Bonn, Bonn, Germany
[4]Institute of Pathology, University Hospital Bonn, Bonn, Germany
[5]Center for Integrated Oncology Aachen Bonn Cologne Duesseldorf (CIO ABCD), Germany
[*]Correspondence: Christian R. Klein (christian.klein@ukbonn.de), ORCID: 0009-0006-1306-7775

Supplementary Methods

This appendix provides a more detailed technical description of the machine learning and statistical methods employed in this study.

1. Machine Learning Model Architectures

Four distinct classes of supervised machine learning models were used:

Random Forest (RF): An ensemble learning method that operates by constructing a multitude of decision trees at training time. For a given prediction, the final output is the mode of the classes (for classification) or the mean prediction (for regression) of the individual trees. RF mitigates overfitting by using bootstrap aggregating (bagging) and random feature selection at each tree split.

Gradient Boosting (GB): Another ensemble method based on decision trees. Unlike RF, which builds trees in parallel, GB builds them sequentially. Each new tree is trained to correct the errors of its predecessor, gradually improving the model's performance by focusing on the most difficult-to-predict instances.

Support Vector Machine (SVM): A model that finds an optimal hyperplane that best separates data points of different classes in the feature space. For non-linearly separable data, SVMs use the "kernel trick" to map the data into a higher-dimensional space where a linear separation becomes possible.

Neural Network (NN): A model inspired by the structure of the human brain, consisting of interconnected layers of nodes (neurons). Each connection has a weight that is adjusted during training via backpropagation. Our implementation used a multi-layer perceptron (MLP) architecture.

2. Mathematical Definitions of Performance Metrics

Let n be the number of observations in the test set, y_i the true value for the i-th observation, and $\hat{y}_i$ the predicted value for the i-th observation. The mean of the true values is denoted by $\bar{y} = \frac{1}{n}\sum_{i=1}^{n} y_i$.

2.1 Regression Metrics

Mean Absolute Error (MAE): Measures the average magnitude of the errors in a set of predictions, without considering their direction.

$$\text{MAE} = \frac{1}{n}\sum_{i=1}^{n} |y_i - \hat{y}_i| \tag{1}$$

Root Mean Squared Error (RMSE): Represents the square root of the average of the squared differences between predicted and actual values. It penalizes larger errors more heavily than MAE.

$$\text{RMSE} = \sqrt{\frac{1}{n}\sum_{i=1}^{n}(y_i - \hat{y}_i)^2} \tag{2}$$

Coefficient of Determination ($\mathbf{R^2}$): Indicates the proportion of the variance in the dependent variable that is predictable from the independent variable(s).

$$\text{R}^2 = 1 - \frac{\sum_{i=1}^{n}(y_i - \hat{y}_i)^2}{\sum_{i=1}^{n}(y_i - \bar{y})^2} \tag{3}$$

Symmetric Mean Absolute Percentage Error (SMAPE): Used in place of MAPE for outcomes where true target values include zero, as standard MAPE requires division by the true value and is therefore mathematically undefined in such cases. We use the following standard symmetric formulation:

$$\text{SMAPE} = \frac{1}{n}\sum_{i=1}^{n}\frac{|y_i - \hat{y}_i|}{(|y_i| + |\hat{y}_i|)/2} \times 100\% \tag{4}$$

In the standard SMAPE formulation above, 0% indicates perfect prediction, whereas higher values indicate greater prediction error. For consistency with the accuracy-like reporting convention used throughout this study, we report the transformed metric

$$\text{SMAPE-based Accuracy} = 100\% - \text{SMAPE}$$

Under this transformation, 100% indicates perfect prediction and lower values indicate greater prediction error. This transformed metric is labelled as "100%–SMAPE" in the Supplementary Tables. Observations for which both the true and predicted values are zero are assigned zero error.

2.2 Classification Metrics

Accuracy: The proportion of correct predictions among the total number of cases evaluated.

$$\text{Accuracy} = \frac{\text{TP} + \text{TN}}{\text{TP} + \text{TN} + \text{FP} + \text{FN}} \tag{5}$$

where TP = True Positives, TN = True Negatives, FP = False Positives, and FN = False Negatives.

Area Under the ROC Curve (AUC): The AUC is a summary of the Receiver Operating Characteristic (ROC) curve, which plots the True Positive Rate (TPR, or Sensitivity) against the False Positive Rate (FPR, or 1 - Specificity) at various classification thresholds. The AUC represents the probability that the model ranks a randomly chosen positive instance higher than a randomly chosen negative one. Mathematically, it is defined as the integral of the ROC curve:

$$\text{AUC} = \int_0^1 \text{TPR}(\text{FPR}^{-1}(x))\,dx \tag{6}$$

Alternatively, the AUC can be defined using the ranking of predictions. For this purpose, let $f(x)$ be the model's output score for an instance x. Let D^+ be the set of positive instances and D^- be the set of negative instances. It can be formally expressed as:

$$\text{AUC} = \frac{\sum_{x_i \in D^+}\sum_{x_j \in D^-}\mathbb{I}[f(x_i) > f(x_j)]}{|D^+| \cdot |D^-|} \tag{7}$$

where $\mathbb{I}[\cdot]$ is the indicator function, which is 1 if the condition inside is true and 0 otherwise. An AUC of 1.0 represents a perfect model, while an AUC of 0.5 indicates performance no better than random chance.

Sensitivity (Recall / True Positive Rate): The proportion of actual positive cases correctly identified by the model.

$$\text{Sensitivity} = \frac{\text{TP}}{\text{TP} + \text{FN}} \tag{8}$$

Specificity (True Negative Rate): The proportion of actual negative cases correctly identified by the model.

$$\text{Specificity} = \frac{\text{TN}}{\text{TN} + \text{FP}} \tag{9}$$

Positive Predictive Value (PPV / Precision): The proportion of positive predictions that are actually correct.

$$\text{PPV} = \frac{\text{TP}}{\text{TP} + \text{FP}} \tag{10}$$

Negative Predictive Value (NPV): The proportion of negative predictions that are actually correct.

$$\text{NPV} = \frac{\text{TN}}{\text{TN} + \text{FN}} \tag{11}$$

For the multiclass outcome Frozen Section Result, all classification metrics were computed using a macro-averaged one-vs-rest (OvR) strategy, in which each class is treated as the positive class in turn and the results are averaged across all classes.

3. Shapley Values and SHAP for Explainable AI

3.1 Theoretical Foundation: Shapley Values

To quantify the contribution of each input feature to a model's prediction, we used a methodology based on the Shapley value, a concept from cooperative game theory. In this framework, the input features are considered "players" in a game, and the "payout" is the prediction of the machine learning model. The Shapley value provides a unique and fair way to distribute this payout among the players [1, 2].

Let $N = \{1, \ldots, m\}$ be the set of all input features and f be the prediction function of the trained ML model. The Shapley value (ϕ_i) for a feature $i \in N$ quantifies its average marginal contribution to the prediction across all possible feature coalitions ($S \subseteq N \setminus \{i\}$):

$$\phi_i(f) = \sum_{S \subseteq N \setminus \{i\}} \frac{|S|! \cdot (|N| - |S| - 1)!}{|N|!} \cdot [f(S \cup \{i\}) - f(S)] \tag{12}$$

This formula ensures that the contribution of each feature is calculated fairly by considering its effect in all possible contexts of other features being present or absent.

3.2 Practical Implementation: Custom Shapley Sampling Framework

SHAP (SHapley Additive exPlanations) is a computational method for calculating Shapley values for machine learning models [3]. Since exact computation of Shapley values is computationally infeasible for most non-trivial models, we implemented a custom permutation-based Shapley sampling algorithm integrated within our cross-validation framework. Instead of relying on standard game-theoretic output decomposition, our algorithm specifically measures the sensitivity of the model's predictive distribution to the progressive removal of information. The algorithm operates as follows: For each test sample, a random permutation of the feature space is generated across a predefined number of sampling runs ($R = 2^{13}$). Features are then sequentially masked one by one. To simulate the absence of a feature within a given coalition, its empirical value is replaced by a fixed baseline placeholder of -1. Following the exclusion of each feature, the model's updated class probability distribution (or continuous prediction output) y_0 is computed. The marginal contribution of the masked feature is defined as the absolute change in the predicted outputs, calculated via the L_1 norm:

$$\Delta y = \sum_{c} |y_c - y_{0,c}| \tag{13}$$

where c denotes the output dimensions (e.g., predicted classes). These marginal deviations are accumulated across all permutation pathways and normalized to yield the final Shapley feature attributions. We explicitly note that the fixed placeholder value serves purely as a technical convention to break the feature signal within the sequential sampling loop, rather than representing a clinically realistic missingness state or a biologically interpretable baseline. To confirm the methodological validity of this approach, a sensitivity analysis was conducted by varying the placeholder value (evaluating baselines of -5, -1, and 0). The resulting feature attributions and global rankings remained exceptionally stable across all tested values, demonstrating that the framework successfully captures robust core feature signals rather than artifacts induced by the out-of-distribution baseline choice. The global feature importance plots shown in the main manuscript display the mean absolute attribution value for each feature, averaged across all test set samples and normalized to sum to 100% for each predictive target. These values provide a global measure of the relative contribution of each feature to the model's predictive behavior and should not be interpreted as causal effect estimates or as direct biological determinants of the respective clinical outcomes.

4. Model Training and Evaluation Strategy

Given the limited sample size (n=326), a nested cross-validation (CV) scheme was employed to ensure robust hyperparameter tuning and unbiased performance evaluation. The outer loop consisted of a 5-fold CV to partition the data into five folds. In each iteration, one fold served as the hold-out test set, while the remaining four folds were used for training. Missing value imputation was performed within the cross-validation pipeline to prevent data leakage. Imputation statistics were computed exclusively on the training folds of each outer iteration and subsequently applied to the held-out test fold. Continuous variables were imputed using the mean, nominal variables using the mode, and ordinal variables using the mean with subsequent rounding to the nearest integer. Feature exclusion decisions based on missingness rates and known clinical redundancies were pre-specified prior to model development based on structural data properties only, and did not involve any outcome-related information. Within this training set, an inner 5-fold CV was performed for hyperparameter optimization across a predefined search space for each model type. The hyperparameter configuration that achieved the highest average performance across the inner folds was selected as optimal. This final model configuration was then re-trained on the entire training set (all four folds) and its performance was evaluated on the hold-out test set from the outer loop. The performance metrics reported in the main manuscript are the average of the results obtained across the five iterations of the outer loop. This nested approach prevents data leakage from the test set into the hyperparameter tuning process, providing a more reliable estimate of the model's generalization performance on unseen data.

Supplementary Data 1

The analyses presented here compare the two primary surgical groups: robot-assisted radical prostatectomy (RARP) and open radical prostatectomy (open). These comparisons are subsequently repeated for the subgroups defined by nerve-sparing status ("nerve sparing" and "no nerve sparing").

For metric variables, histograms are used for graphical representation. For nominal and ordinal variables, bar charts and cross-tabulations are provided. The assumption of normal distribution for metric variables was assessed using the Shapiro-Wilk test. If the data were not normally distributed in at least one group ($p < 0.05$), the Mann-Whitney U test was used for group comparisons. Otherwise, an independent samples t-test was employed. Effect sizes were calculated as Cohen's d for t-tests or as the absolute standardized test statistic divided by the square root of the sample size for non-parametric tests.

	**RARP**	**Open**	**Total**
Nerve Sparing	127 (39.1%)	58 (17.8%)	187 (56.9%)
No Nerve Sparing	96 (29.5%)	44 (13.5%)	140 (43.1%)
Missing	1	0	1
**Total**	**224**	**102**	**326**

Table 1: Sample size and distribution of nerve-sparing status.

Result of Fisher's exact test for the distribution of nerve-sparing status (excluding missing values): p=0.132

Age (years)

Total Cohort	RARP	Open
Number of patients	224	102
Median (IQR)	67 (62–72)	65 (60–69)
Missing values	0	0

Table 2: Descriptive statistics for Age in the total cohort. Normality was assessed with the Shapiro-Wilk test (p=0.023 for RARP, p=0.306 for Open). Comparison was performed using the Mann-Whitney U test (p=0.002, effect size r=0.172).

Subgroup: Nerve Sparing	RARP	Open
Number of patients	127	58
Median (IQR)	65 (60–71)	65 (61–68)
Missing values	0	0

Table 3: Descriptive statistics for Age in the nerve-sparing subgroup. Comparison was performed using the Mann-Whitney U test (p=0.629, effect size r=0.036).

Subgroup: No Nerve Sparing	RARP	Open
Number of patients	96	44
Median (IQR)	70 (66–75)	64 (59–69)
Missing values	0	0

Table 4: Descriptive statistics for Age in the no-nerve-sparing subgroup. Comparison was performed using the Mann-Whitney U test (p<0.001, effect size r=0.339).

Prostate Volume (ml)

**Total Cohort**	**RARP**	**Open**
Number of patients	204	88
Median (IQR)	40.0 (30.0–50.0)	35.0 (25.0–47.5)
Missing values	20	14

Table 5: Descriptive statistics for Prostate Volume in the total cohort. Normality was assessed with the Shapiro-Wilk test ($p<0.001$ for both groups). Comparison was performed using the Mann-Whitney U test ($p=0.056$, effect size $r=0.111$).

**Subgroup: Nerve Sparing**	**RARP**	**Open**
Number of patients	115	52
Median (IQR)	40.0 (30.0–50.0)	32.0 (25.0–45.0)
Missing values	12	6

Table 6: Descriptive statistics for Prostate Volume in the nerve-sparing subgroup. Comparison was performed using the Mann-Whitney U test ($p=0.106$, effect size $r=0.125$).

**Subgroup: No Nerve Sparing**	**RARP**	**Open**
Number of patients	88	36
Median (IQR)	40.0 (30.0–50.0)	40.0 (26.5–54.0)
Missing values	8	8

Table 7: Descriptive statistics for Prostate Volume in the no-nerve-sparing subgroup. Comparison was performed using the Mann-Whitney U test ($p=0.334$, effect size $r=0.087$).

Initial PSA Value (ng/ml)

**Total Cohort**	**RARP**	**Open**
Number of patients	224	102
Median (IQR)	7.65 (5.57–10.95)	7.83 (5.40–12.60)
Missing values	0	0

Table 8: Descriptive statistics for Initial PSA Value in the total cohort. Normality was assessed with the Shapiro-Wilk test ($p<0.001$ for both groups). Comparison was performed using the Mann-Whitney U test ($p=0.845$, effect size $r=0.011$).

**Subgroup: Nerve Sparing**	**RARP**	**Open**
Number of patients	127	58
Median (IQR)	7.03 (5.40–9.90)	6.23 (5.21–8.98)
Missing values	0	0

Table 9: Descriptive statistics for Initial PSA Value in the nerve-sparing subgroup. Comparison was performed using the Mann-Whitney U test ($p=0.344$, effect size $r=0.069$).

**Subgroup: No Nerve Sparing**	**RARP**	**Open**
Number of patients	96	44
Median (IQR)	8.99 (5.88–14.95)	9.74 (7.01–20.55)
Missing values	0	0

Table 10: Descriptive statistics for Initial PSA Value in the no-nerve-sparing subgroup. Comparison was performed using the Mann-Whitney U test ($p=0.194$, effect size $r=0.110$).

Preoperative ISUP Grade

Total Cohort	Grade 1	Grade 2	Grade 3	Grade 4	Grade 5	Missing
RARP	40 (18.0%)	85 (38.3%)	45 (20.3%)	31 (14.0%)	21 (9.5%)	2
Open	14 (13.7%)	29 (28.4%)	16 (15.7%)	19 (18.6%)	24 (23.5%)	0

Table 11: Cross-tabulation for Preoperative ISUP Grade in the total cohort. Values are presented as n (%). Comparison was performed using the Chi-squared test (p=0.006).

Subgroup: Nerve Sparing	Grade 1	Grade 2	Grade 3	Grade 4	Grade 5	Missing
RARP	35 (27.8%)	54 (42.9%)	23 (18.3%)	11 (8.7%)	3 (2.4%)	1
Open	5 (8.5%)	29 (49.2%)	9 (15.3%)	10 (16.9%)	6 (10.2%)	0

Table 12: Cross-tabulation for Preoperative ISUP Grade in the nerve-sparing subgroup. Values are presented as n (%). Comparison was performed using the Chi-squared test (p=0.152).

Subgroup: No Nerve Sparing	Grade 1	Grade 2	Grade 3	Grade 4	Grade 5	Missing
RARP	5 (5.3%)	31 (32.6%)	21 (22.1%)	20 (21.1%)	18 (18.9%)	1
Open	3 (6.8%)	7 (15.9%)	5 (11.4%)	10 (22.7%)	19 (43.2%)	0

Table 13: Cross-tabulation for Preoperative ISUP Grade in the no-nerve-sparing subgroup. Values are presented as n (%). Comparison was performed using the Chi-squared test (p=0.019).

D'Amico Risk Classification

Total Cohort	Low Risk	Intermediate Risk	High Risk	Missing
RARP	22 (9.8%)	100 (44.6%)	102 (45.5%)	0
Open	8 (8.8%)	33 (36.3%)	50 (54.9%)	11

Table 14: Cross-tabulation for D'Amico Risk Classification in the total cohort. Values are presented as n (%). Comparison was performed using the Chi-squared test (p=0.311).

Subgroup: Nerve Sparing	Low Risk	Intermediate Risk	High Risk	Missing
RARP	19 (15.0%)	72 (56.7%)	36 (28.3%)	0
Open	6 (11.5%)	29 (55.8%)	17 (32.7%)	6

Table 15: Cross-tabulation for D'Amico Risk Classification in the nerve-sparing subgroup. Values are presented as n (%). Comparison was performed using the Chi-squared test (p=0.759).

Preoperative Hemoglobin (g/dl)

Total Cohort	RARP	Open
Number of patients	220	102
Median (IQR)	14.80 (14.25–15.60)	15.00 (14.10–15.60)
Missing values	4	0

Table 16: Descriptive statistics for Preoperative Hemoglobin in the total cohort. Normality was assessed with the Shapiro-Wilk test ($p<0.001$ for both groups). Comparison was performed using the Mann-Whitney U test ($p=0.979$, effect size $r=0.001$).

Subgroup: Nerve Sparing	RARP	Open
Number of patients	123	58
Median (IQR)	15.10 (14.50–15.70)	15.10 (14.20–15.70)
Missing values	4	0

Table 17: Descriptive statistics for Preoperative Hemoglobin in the nerve-sparing subgroup. Comparison was performed using the Mann-Whitney U test ($p=0.507$, effect size $r=0.049$).

Subgroup: No Nerve Sparing	RARP	Open
Number of patients	96	44
Median (IQR)	14.60 (13.90–15.30)	14.90 (14.00–15.45)
Missing values	0	0

Table 18: Descriptive statistics for Preoperative Hemoglobin in the no-nerve-sparing subgroup. Comparison was performed using the Mann-Whitney U test ($p=0.194$, effect size $r=0.110$).

Body Mass Index (kg/m^2)

Total Cohort	RARP	Open
Number of patients	213	58
Median (IQR)	26.50 (24.30–28.90)	26.25 (23.50–31.00)
Missing values	11	44

Table 19: Descriptive statistics for BMI in the total cohort. Normality was assessed with the Shapiro-Wilk test ($p<0.001$ for RARP, $p=0.039$ for Open). Comparison was performed using the Mann-Whitney U test ($p=0.785$, effect size $r=0.017$).

Subgroup: Nerve Sparing	RARP	Open
Number of patients	122	30
Median (IQR)	26.50 (24.30–28.70)	25.40 (23.40–31.30)
Missing values	5	28

Table 20: Descriptive statistics for BMI in the nerve-sparing subgroup. Comparison was performed using the Mann-Whitney U test ($p=0.967$, effect size $r=0.003$).

Subgroup: No Nerve Sparing	RARP	Open
Number of patients	90	28
Median (IQR)	26.45 (24.50–28.90)	27.00 (23.70–30.90)
Missing values	6	16

Table 21: Descriptive statistics for BMI in the no-nerve-sparing subgroup. Comparison was performed using the Mann-Whitney U test ($p=0.194$, effect size $r=0.110$).

ASA Physical Status Classification

Total Cohort	ASA 1	ASA 2	ASA 3	Missing
RARP	31 (14.2%)	145 (66.5%)	42 (19.3%)	6
Open	17 (17.7%)	60 (62.5%)	19 (19.8%)	6

Table 22: Cross-tabulation for ASA Classification in the total cohort. Values are presented as n (%). Comparison was performed using the Chi-squared test (p=0.703).

Subgroup: Nerve Sparing	ASA 1	ASA 2	ASA 3	Missing
RARP	21 (16.8%)	89 (71.2%)	15 (12.0%)	2
Open	11 (20.8%)	34 (64.2%)	8 (15.1%)	5

Table 23: Cross-tabulation for ASA Classification in the nerve-sparing subgroup. Values are presented as n (%). Comparison was performed using the Chi-squared test (p=0.648).

Subgroup: No Nerve Sparing	ASA 1	ASA 2	ASA 3	Missing
RARP	10 (10.9%)	56 (60.9%)	26 (28.3%)	4
Open	6 (14.0%)	26 (60.5%)	11 (25.6%)	1

Table 24: Cross-tabulation for ASA Classification in the no-nerve-sparing subgroup. Values are presented as n (%). Comparison was performed using the Chi-squared test (p=0.855).

ICIQ Continence Score (ICIQ-SF Part 1)

Total Cohort	RARP	Open
Number of patients	179	0
Mean (SD)	1.60 (2.87)	–
Median (IQR)	0 (0–3)	–
Missing values	45	102

Table 25: Descriptive statistics for the ICIQ continence score in the total cohort. Data for the open surgery group was not available. Normality for the RARP group was assessed with the Shapiro-Wilk test ($p<0.001$).

Subgroup: Nerve Sparing	RARP
Number of patients	104
Mean (SD)	1.63 (2.90)
Median (IQR)	0 (0–3)
Missing values	23

Table 26: Descriptive statistics for the ICIQ continence score in the nerve-sparing subgroup. Data was only available for the RARP group.

Subgroup: No Nerve Sparing	RARP
Number of patients	74
Mean (SD)	1.50 (2.82)
Median (IQR)	0 (0–2)
Missing values	22

Table 27: Descriptive statistics for the ICIQ continence score in the no-nerve-sparing subgroup. Data was only available for the RARP group.

International Prostate Symptom Score (IPSS)

Total Cohort	RARP	Open
Number of patients	193	11
Median (IQR)	11 (6–18)	12 (11–24)
Missing values	31	91

Table 28: Descriptive statistics for IPSS in the total cohort. Normality was assessed with the Shapiro-Wilk test ($p<0.001$ for RARP, $p=0.324$ for Open). Comparison was performed using the Mann-Whitney U test ($p=0.258$, effect size $r=0.079$).

Subgroup: Nerve Sparing	RARP	Open
Number of patients	114	6
Median (IQR)	9 (6–17)	21 (11–25)
Missing values	13	52

Table 29: Descriptive statistics for IPSS in the nerve-sparing subgroup. Comparison was performed using the Mann-Whitney U test ($p=0.086$, effect size $r=0.157$).

Subgroup: No Nerve Sparing	RARP	Open
Number of patients	78	5
Median (IQR)	13 (6–21)	12 (12–12)
Missing values	18	39

Table 30: Descriptive statistics for IPSS in the no-nerve-sparing subgroup. Comparison was performed using the Mann-Whitney U test ($p=0.717$, effect size $r=0.078$).

International Index of Erectile Function (IIEF)

**Total Cohort**	**RARP**	**Open**
Number of patients	193	19
Median (IQR)	17 (9–22)	29 (19–30)
Missing values	31	83

Table 31: Descriptive statistics for IIEF in the total cohort. Normality was assessed with the Shapiro-Wilk test ($p<0.001$ for both groups). Comparison was performed using the Mann-Whitney U test ($p<0.001$, effect size $r=0.284$).

**Subgroup: Nerve Sparing**	**RARP**	**Open**
Number of patients	116	12
Median (IQR)	18 (13–23)	28 (22–30)
Missing values	11	46

Table 32: Descriptive statistics for IIEF in the nerve-sparing subgroup. Comparison was performed using the Mann-Whitney U test ($p=0.002$, effect size $r=0.276$).

**Subgroup: No Nerve Sparing**	**RARP**	**Open**
Number of patients	76	7
Median (IQR)	13 (4–20)	29 (14–30)
Missing values	20	37

Table 33: Descriptive statistics for IIEF in the no-nerve-sparing subgroup. Comparison was performed using the Mann-Whitney U test ($p=0.007$, effect size $r=0.297$).

Preoperative ADT Treatment

Total Cohort	ADT: No	ADT: Yes	Missing
RARP	214 (96.0%)	9 (4.0%)	1
Open	94 (93.1%)	7 (6.9%)	1

Table 34: Cross-tabulation for preoperative ADT treatment in the total cohort. Values are presented as n (%). Comparison was performed using Fisher's exact test (p=0.277).

Subgroup: Nerve Sparing	ADT: No	ADT: Yes	Missing
RARP	125 (98.4%)	2 (1.6%)	0
Open	56 (98.2%)	1 (1.8%)	1

Table 35: Cross-tabulation for preoperative ADT treatment in the nerve-sparing subgroup. Values are presented as n (%). Comparison was performed using Fisher's exact test (p=1.000).

Subgroup: No Nerve Sparing	ADT: No	ADT: Yes	Missing
RARP	89 (92.7%)	7 (7.3%)	0
Open	38 (86.4%)	6 (13.6%)	0

Table 36: Cross-tabulation for preoperative ADT treatment in the no-nerve-sparing subgroup. Values are presented as n (%). Comparison was performed using Fisher's exact test (p=0.346).

Subgroup: Patients with ADT	Nerve Sparing	No Nerve Sparing	Total
RARP	2	7	9
Open	1	6	7

Table 37: Cross-tabulation showing the distribution of nerve-sparing status among the subgroup of patients who received preoperative ADT (n=16). Values are presented as absolute counts. Comparison between RARP and Open groups was performed using Fisher's exact test (p=1.000).

Intraoperative Blood Loss (ml)

Total Cohort	RARP	Open
Number of patients	222	67
Median (IQR)	400 (300–500)	1200 (800–1500)
Missing values	2	35

Table 38: Descriptive statistics for Intraoperative Blood Loss in the total cohort. Normality was assessed with the Shapiro-Wilk test ($p<0.001$ for both groups). Comparison was performed using the Mann-Whitney U test ($p<0.001$, effect size $r=0.594$).

Subgroup: Nerve Sparing	RARP	Open
Number of patients	127	40
Median (IQR)	400 (300–600)	1200 (800–1500)
Missing values	0	18

Table 39: Descriptive statistics for Intraoperative Blood Loss in the nerve-sparing subgroup. Comparison was performed using the Mann-Whitney U test ($p<0.001$, effect size $r=0.564$).

Subgroup: No Nerve Sparing	RARP	Open
Number of patients	95	27
Median (IQR)	400 (300–500)	1200 (900–1500)
Missing values	1	17

Table 40: Descriptive statistics for Intraoperative Blood Loss in the no-nerve-sparing subgroup. Comparison was performed using the Mann-Whitney U test ($p<0.001$, effect size $r=0.637$).

Operative Time (minutes)

Total Cohort	RARP (Console Time)	Open (Surgical Time)
Number of patients	197	101
Mean (SD)	143 (approx. SD)	155 (approx. SD)
Median (IQR)	141 (123–161)	155 (139–172)
Missing values	27	1

Table 41: Descriptive statistics for Operative Time in the total cohort. For RARP, console time is reported; for open surgery, total surgical time is reported. Normality was assessed with the Shapiro-Wilk test ($p=0.044$ for RARP, $p<0.001$ for Open). Comparison was performed using the Mann-Whitney U test ($p<0.001$, effect size $r=0.230$).

Subgroup: Nerve Sparing	RARP (Console Time)	Open (Surgical Time)
Number of patients	111	58
Median (IQR)	141.0 (124.0–160.0)	151.5 (136.0–170.0)
Missing values	16	0

Table 42: Descriptive statistics for Operative Time in the nerve-sparing subgroup. Comparison was performed using the Mann-Whitney U test ($p=0.004$, effect size $r=0.220$).

Subgroup: No Nerve Sparing	RARP (Console Time)	Open (Surgical Time)
Number of patients	85	43
Median (IQR)	144 (123–171)	160 (149–172)
Missing values	11	1

Table 43: Descriptive statistics for Operative Time in the no-nerve-sparing subgroup. Comparison was performed using the Mann-Whitney U test ($p=0.009$, effect size $r=0.232$).

Postoperative Hemoglobin (g/dl)

Total Cohort	RARP	Open
Number of patients	224	101
Median (IQR)	12.95 (12.00–13.60)	10.20 (9.30–11.50)
Missing values	0	1

Table 44: Descriptive statistics for Postoperative Hemoglobin in the total cohort. Normality was assessed with the Shapiro-Wilk test ($p=0.001$ for RARP, $p=0.016$ for Open). Comparison was performed using the Mann-Whitney U test ($p<0.001$, effect size $r=0.601$).

Subgroup: Nerve Sparing	RARP	Open
Number of patients	127	58
Median (IQR)	13.10 (12.10–13.70)	10.15 (9.40–11.40)
Missing values	0	0

Table 45: Descriptive statistics for Postoperative Hemoglobin in the nerve-sparing subgroup. Comparison was performed using the Mann-Whitney U test ($p<0.001$, effect size $r=0.648$).

Subgroup: No Nerve Sparing	RARP	Open
Number of patients	96	43
Median (IQR)	12.55 (11.55–13.40)	10.40 (9.00–11.70)
Missing values	0	1

Table 46: Descriptive statistics for Postoperative Hemoglobin in the no-nerve-sparing subgroup. Comparison was performed using the Mann-Whitney U test ($p<0.001$, effect size $r=0.535$).

Intraoperative Fluid Administration (ml)

**Total Cohort**	**RARP**	**Open**
Number of patients	223	73
Median (IQR)	2300 (1500–3000)	3500 (2500–4500)
Missing values	1	29

Table 47: Descriptive statistics for Intraoperative Fluid Administration in the total cohort. Normality was assessed with the Shapiro-Wilk test ($p<0.001$ for both groups). Comparison was performed using the Mann-Whitney U test ($p<0.001$, effect size $r=0.416$).

**Subgroup: Nerve Sparing**	**RARP**	**Open**
Number of patients	127	38
Median (IQR)	2200 (1500–3000)	3500 (2500–4000)
Missing values	0	20

Table 48: Descriptive statistics for Intraoperative Fluid Administration in the nerve-sparing subgroup. Comparison was performed using the Mann-Whitney U test ($p<0.001$, effect size $r=0.402$).

**Subgroup: No Nerve Sparing**	**RARP**	**Open**
Number of patients	95	32
Median (IQR)	2500 (2000–2575)	3500 (2750–4500)
Missing values	1	12

Table 49: Descriptive statistics for Intraoperative Fluid Administration in the no-nerve-sparing subgroup. Comparison was performed using the Mann-Whitney U test ($p<0.001$, effect size $r=0.426$).

Postoperative Hemoglobin Drop (g/dl)

**Total Cohort**	**RARP**	**Open**
Number of patients	220	101
Median (IQR)	2.10 (1.40–2.65)	4.30 (3.40–5.20)
Missing values	4	1

Table 50: Descriptive statistics for the Postoperative Hemoglobin Drop in the total cohort. Normality was assessed with the Shapiro-Wilk test ($p<0.001$ for RARP, $p=0.764$ for Open). Comparison was performed using the Mann-Whitney U test ($p<0.001$, effect size $r=0.667$).

**Subgroup: Nerve Sparing**	**RARP**	**Open**
Number of patients	123	58
Median (IQR)	2.00 (1.40–2.70)	4.50 (3.40–5.20)
Missing values	4	0

Table 51: Descriptive statistics for the Postoperative Hemoglobin Drop in the nerve-sparing subgroup. Comparison was performed using the Mann-Whitney U test ($p<0.001$, effect size $r=0.642$).

**Subgroup: No Nerve Sparing**	**RARP**	**Open**
Number of patients	96	43
Median (IQR)	2.10 (1.50–2.65)	4.10 (3.10–5.20)
Missing values	0	1

Table 52: Descriptive statistics for the Postoperative Hemoglobin Drop in the no-nerve-sparing subgroup. Comparison was performed using the Mann-Whitney U test ($p<0.001$, effect size $r=0.698$).

Extent of Nerve Sparing

Detailed Nerve Sparing	None	Right only	Left only	Bilateral	Missing
RARP	96 (43.0%)	24 (10.8%)	32 (14.3%)	71 (31.8%)	1
Open	43 (42.6%)	11 (10.9%)	18 (17.8%)	29 (28.7%)	1

Table 53: Cross-tabulation for the detailed extent of nerve sparing. Values are presented as n (%). Comparison between RARP and Open groups was performed using the Chi-squared test (p=0.856).

Simplified Nerve Sparing	None	Unilateral	Bilateral	Missing
RARP	96 (43.0%)	56 (25.1%)	71 (31.8%)	1
Open	43 (42.6%)	29 (28.7%)	29 (28.7%)	1

Table 54: Cross-tabulation for the simplified extent of nerve sparing, where unilateral preservation (right or left) is grouped. Values are presented as n (%). Comparison between RARP and Open groups was performed using the Chi-squared test (p=0.756).

Pathological T-Stage (simplified)

Total Cohort	pT2	pT3	pT4	Missing
RARP	109 (48.7%)	115 (51.3%)	0 (0.0%)	0
Open	64 (62.7%)	37 (36.3%)	1 (1.0%)	0

Table 55: Cross-tabulation for pathological T-Stage in the total cohort. Values are presented as n (%). Comparison was performed using the Chi-squared test (p=0.009).

Subgroup: Nerve Sparing	pT2	pT3	Missing
RARP	77 (60.6%)	50 (39.4%)	0
Open	45 (77.6%)	13 (22.4%)	0

Table 56: Cross-tabulation for pathological T-Stage in the nerve-sparing subgroup. Values are presented as n (%). Comparison was performed using the Chi-squared test (p=0.030).

Subgroup: No Nerve Sparing	pT2	pT3	pT4	Missing
RARP	32 (33.3%)	64 (66.7%)	0 (0.0%)	0
Open	19 (43.2%)	24 (54.5%)	1 (2.3%)	0

Table 57: Cross-tabulation for pathological T-Stage in the no-nerve-sparing subgroup. Values are presented as n (%). Comparison was performed using the Chi-squared test (p=0.141).

Venous Invasion (V-Status)

Total Cohort	V0	V1	Missing
RARP	217 (96.9%)	7 (3.1%)	0
Open	92 (92.9%)	7 (7.1%)	3

Table 58: Cross-tabulation for Venous Invasion (V-Status) in the total cohort. Values are presented as n (%). Comparison was performed using Fisher's exact test (p=0.138).

Subgroup: Nerve Sparing	V0	V1	Missing
RARP	124 (97.6%)	3 (2.4%)	0
Open	53 (94.6%)	3 (5.4%)	2

Table 59: Cross-tabulation for Venous Invasion in the nerve-sparing subgroup. Fisher's exact test: p=0.372.

Subgroup: No Nerve Sparing	V0	V1	Missing
RARP	92 (95.8%)	4 (4.2%)	0
Open	39 (90.7%)	4 (9.3%)	1

Table 60: Cross-tabulation for Venous Invasion in the no-nerve-sparing subgroup. Fisher's exact test: p=0.253.

Lymphatic Invasion (L-Status)

Total Cohort	L0	L1	Missing
RARP	206 (92.4%)	17 (7.6%)	1
Open	82 (84.5%)	15 (15.5%)	5

Table 61: Cross-tabulation for Lymphatic Invasion (L-Status) in the total cohort. Values are presented as n (%). Comparison was performed using Fisher's exact test (p=0.042).

Subgroup: Nerve Sparing	L0	L1	Missing
RARP	122 (96.8%)	4 (3.2%)	1
Open	51 (91.1%)	5 (8.9%)	2

Table 62: Cross-tabulation for Lymphatic Invasion in the nerve-sparing subgroup. Fisher's exact test: p=0.136.

Subgroup: No Nerve Sparing	L0	L1	Missing
RARP	84 (87.5%)	12 (12.5%)	0
Open	31 (75.6%)	10 (24.4%)	3

Table 63: Cross-tabulation for Lymphatic Invasion in the no-nerve-sparing subgroup. Fisher's exact test: p=0.051.

Postoperative ISUP Grade

Total Cohort	Grade 1	Grade 2	Grade 3	Grade 4	Grade 5	Missing
RARP	7 (3.2%)	95 (43.2%)	66 (30.0%)	11 (5.0%)	41 (18.6%)	4
Open	5 (5.0%)	37 (36.6%)	19 (18.8%)	18 (17.8%)	22 (21.8%)	1

Table 64: Cross-tabulation for Postoperative ISUP Grade in the total cohort. Values are presented as n (%). Comparison was performed using the Chi-squared test (p=0.001).

Subgroup: Nerve Sparing	Grade 1	Grade 2	Grade 3	Grade 4	Grade 5	Missing
RARP	6 (4.8%)	75 (59.5%)	32 (25.4%)	3 (2.4%)	10 (7.9%)	1
Open	5 (8.8%)	28 (49.1%)	9 (15.8%)	10 (17.5%)	5 (8.8%)	1

Table 65: Cross-tabulation for Postoperative ISUP Grade in the nerve-sparing subgroup. Chi-squared test: p=0.002.

Subgroup: No Nerve Sparing	Grade 1	Grade 2	Grade 3	Grade 4	Grade 5	Missing
RARP	1 (1.1%)	20 (21.5%)	34 (36.6%)	8 (8.6%)	30 (32.3%)	3
Open	0 (0.0%)	9 (20.5%)	10 (22.7%)	8 (18.2%)	17 (38.6%)	0

Table 66: Cross-tabulation for Postoperative ISUP Grade in the no-nerve-sparing subgroup. Chi-squared test: p=0.285.

Harvested Lymph Node Count

**Total Cohort**	**RARP**	**Open**
Number of patients	223	101
Median (IQR)	10 (7–14)	21 (15–27)
Missing values	1	1

Table 67: Descriptive statistics for Harvested Lymph Node Count. Normality was assessed with the Shapiro-Wilk test ($p<0.001$ for RARP, $p=0.007$ for Open). Comparison was performed using the Mann-Whitney U test ($p<0.001$, effect size $r=0.547$).

**Subgroup: Nerve Sparing**	**RARP**	**Open**
Number of patients	126	58
Median (IQR)	9 (6–13)	21 (16–30)
Missing values	1	0

Table 68: Descriptive statistics for Harvested Lymph Node Count in the nerve-sparing subgroup. Mann-Whitney U test: $p<0.001$, effect size $r=0.611$.

**Subgroup: No Nerve Sparing**	**RARP**	**Open**
Number of patients	96	43
Median (IQR)	12 (8–16)	20 (14–24)
Missing values	0	1

Table 69: Descriptive statistics for Harvested Lymph Node Count in the no-nerve-sparing subgroup. Mann-Whitney U test: $p<0.001$, effect size $r=0.442$.

Positive Lymph Node Count (in N1 patients)

**Total Cohort (N1 only)**	**RARP**	**Open**
Number of patients	31	21
Median (IQR)	1 (1–3)	1 (1–2)
Missing values	0	0

Table 70: Descriptive statistics for Positive Lymph Node Count in N1 patients. Mann-Whitney U test: p=0.502, effect size r=0.093.

**Subgroup: Nerve Sparing (N1 only)**	**RARP**	**Open**
Number of patients	6	7
Median (IQR)	1 (1–2)	1 (1–2)
Missing values	0	0

Table 71: Descriptive statistics for Positive Lymph Node Count in the nerve-sparing subgroup (N1 only). Mann-Whitney U test: p=0.836, effect size r=0.073.

**Subgroup: No Nerve Sparing (N1 only)**	**RARP**	**Open**
Number of patients	25	14
Median (IQR)	2 (1–3)	1 (1–2)
Missing values	0	0

Table 72: Descriptive statistics for Positive Lymph Node Count in the no-nerve-sparing subgroup (N1 only). Mann-Whitney U test: p=0.740, effect size r=0.061.

Pathological N-Status

Total Cohort	N0	N1	Missing
RARP	192 (86.1%)	31 (13.9%)	1
Open	78 (78.8%)	21 (21.2%)	3

Table 73: Cross-tabulation for pathological N-Status. Fisher's exact test: p=0.104.

Subgroup: Nerve Sparing	N0	N1	Missing
RARP	120 (95.2%)	6 (4.8%)	1
Open	49 (87.5%)	7 (12.5%)	2

Table 74: Cross-tabulation for pathological N-Status in the nerve-sparing subgroup. Fisher's exact test: p=0.104.

Subgroup: No Nerve Sparing	N0	N1	Missing
RARP	71 (74.0%)	25 (26.0%)	0
Open	14 (32.6%)	29 (67.4%)	1

Table 75: Cross-tabulation for pathological N-Status in the no-nerve-sparing subgroup. Fisher's exact test: p<0.001.

Resection Margin Status (R-Status)

Total Cohort	R0	R1	Missing
RARP	172 (77.1%)	51 (22.9%)	1
Open	60 (60.0%)	40 (40.0%)	2

Table 76: Cross-tabulation for Resection Margin Status. Fisher's exact test: p=0.002.

Subgroup: Nerve Sparing	R0	R1	Missing
RARP	103 (81.7%)	23 (18.3%)	1
Open	37 (64.9%)	20 (35.1%)	1

Table 77: Cross-tabulation for Resection Margin Status in the nerve-sparing subgroup. Fisher's exact test: p=0.015.

Subgroup: No Nerve Sparing	R0	R1	Missing
RARP	68 (70.8%)	28 (29.2%)	0
Open	23 (53.5%)	20 (46.5%)	0

Table 78: Cross-tabulation for Resection Margin Status in the no-nerve-sparing subgroup. Fisher's exact test: p=0.055.

Pathological T-Stage stratified by Resection Margin Status

Total Cohort: T-Stage within R1 Patients	pT2	pT3	pT4
R1 Patients (n=91)	28 (30.8%)	63 (69.2%)	0 (0.0%)

Table 79: Distribution of pathological T-stages among patients with positive resection margins (R1).

Total Cohort: R-Status within T-Stage	R0	R1
pT2 Patients (n=171)	143 (83.6%)	28 (16.4%)
pT3 Patients (n=151)	88 (58.3%)	63 (41.7%)

Table 80: Distribution of resection margin status for different pathological T-stages. Fisher's exact test: $p<0.001$.

Intraoperative Frozen Section (FS) Utilization and Results

Utilization of Frozen Section

Total Cohort	FS Performed	FS Not Performed	Missing
RARP	119 (53.4%)	104 (46.6%)	1
Open	38 (40.4%)	56 (59.6%)	8

Table 81: Cross-tabulation for the utilization of intraoperative frozen section. Fisher's exact test: p=0.037.

Subgroup: Nerve Sparing	FS Performed	FS Not Performed	Missing
RARP	115 (90.6%)	12 (9.4%)	0
Open	22 (43.1%)	29 (56.9%)	7

Table 82: Cross-tabulation for FS utilization in the nerve-sparing subgroup. Fisher's exact test: p<0.001.

Subgroup: No Nerve Sparing	FS Performed	FS Not Performed	Missing
RARP	4 (4.2%)	92 (95.8%)	0
Open	16 (37.2%)	27 (62.8%)	1

Table 83: Cross-tabulation for FS utilization in the no-nerve-sparing subgroup. Fisher's exact test: p<0.001.

Results of Frozen Section (in patients with FS)

Total Cohort (FS performed)	Positive FS	Negative FS
RARP (n=119)	13 (10.9%)	106 (89.1%)
Open (n=38)	8 (21.1%)	30 (78.9%)

Table 84: Results of frozen section analysis in the total cohort. Fisher's exact test: p=0.168.

Subgroup: Nerve Sparing (FS performed)	Positive FS	Negative FS
RARP (n=115)	13 (11.3%)	102 (88.7%)
Open (n=22)	4 (18.2%)	18 (81.8%)

Table 85: Results of frozen section analysis in the nerve-sparing subgroup. Fisher's exact test: p=0.477.

Subgroup: No Nerve Sparing (FS performed)	Positive FS	Negative FS
RARP (n=4)	0 (0.0%)	4 (100.0%)
Open (n=16)	4 (25.0%)	12 (75.0%)

Table 86: Results of frozen section analysis in the no-nerve-sparing subgroup. Fisher's exact test: p=0.538.

Length of Hospital Stay (days)

**Total Cohort**	**RARP**	**Open**
Number of patients	223	102
Median (IQR)	6 (6–7)	10 (9–10)
Missing values	1	0

Table 87: Descriptive statistics for Length of Hospital Stay. Mann-Whitney U test: $p<0.001$, effect size $r=0.780$.

**Subgroup: Nerve Sparing**	**RARP**	**Open**
Number of patients	127	58
Median (IQR)	6 (6–6)	10 (9–10)
Missing values	0	0

Table 88: Descriptive statistics for Length of Hospital Stay in the nerve-sparing subgroup. Mann-Whitney U test: $p<0.001$, effect size $r=0.797$.

**Subgroup: No Nerve Sparing**	**RARP**	**Open**
Number of patients	95	44
Median (IQR)	6 (6–7)	10 (9–10)
Missing values	1	0

Table 89: Descriptive statistics for Length of Hospital Stay in the no-nerve-sparing subgroup. Mann-Whitney U test: $p<0.001$, effect size $r=0.763$.

Catheter Dwell Time (days)

**Total Cohort**	**RARP**	**Open**
Number of patients	219	96
Median (IQR)	5 (5–5)	8 (8–8.5)
Missing values	5	6

Table 90: Descriptive statistics for Catheter Dwell Time. Mann-Whitney U test: $p<0.001$, effect size $r=0.802$.

**Subgroup: Nerve Sparing**	**RARP**	**Open**
Number of patients	124	56
Median (IQR)	5 (5–5)	8 (8–8)
Missing values	3	2

Table 91: Descriptive statistics for Catheter Dwell Time in the nerve-sparing subgroup. Mann-Whitney U test: $p<0.001$, effect size $r=0.869$.

**Subgroup: No Nerve Sparing**	**RARP**	**Open**
Number of patients	94	40
Median (IQR)	5 (5–5)	8 (8–13)
Missing values	2	4

Table 92: Descriptive statistics for Catheter Dwell Time in the no-nerve-sparing subgroup. Mann-Whitney U test: $p<0.001$, effect size $r=0.723$.

Postoperative Complications (Clavien-Dindo Classification)

Overall Complication Grades (Minor/Major)

Total Cohort	No Complication	Minor (I-IIIa)	Major (IIIb-V)	Missing
RARP	110 (49.1%)	99 (44.2%)	15 (6.7%)	0
Open	45 (44.1%)	44 (43.1%)	13 (12.7%)	0

Table 93: Distribution of overall Clavien-Dindo complication grades. Chi-squared test: p=0.185.

Analysis of Minor Complications

Total Cohort (Minor only)	Grade I	Grade II
RARP (n=99)	88 (88.9%)	11 (11.1%)
Open (n=44)	31 (70.5%)	13 (29.5%)

Table 94: Distribution of Grade I vs. Grade II within minor complications. Fisher's exact test: p=0.009.

Types of Minor Complications (Total Cohort)	Count (n=197)	Proportion (%)
Lymphocele	105	53.3%
Anastomotic insufficiency	31	15.7%
Hematoma	14	7.1%
Transfusion	14	7.1%
Urinary retention	10	5.1%
Infection	8	4.1%

Table 95: Itemization of the most frequent minor complications.

Analysis of Major Complications

Total Cohort (Major only)	Grade IIIa	Grade IIIb	Grade IV	Grade V
RARP (n=15)	8 (53.3%)	5 (33.3%)	1 (6.7%)	1 (6.7%)
Open (n=13)	6 (46.2%)	7 (53.8%)	0 (0.0%)	0 (0.0%)

Table 96: Distribution of grades within major complications. Chi-squared test: p=0.622.

Types of Major Complications (Total Cohort)	Count (n=70)	Proportion (%)
Lymphocele	17	24.3%
Re-operation (Re-OP)	14	20.0%
Anastomotic insufficiency	6	8.6%
Transfusion	6	8.6%
Abdominal dehiscence (Platzbauch)	5	7.1%

Table 97: Itemization of the most frequent major complications.

Summary of Key Descriptive Findings

This comprehensive descriptive and statistical analysis of the patient cohort (n=326) revealed several key baseline differences between the robot-assisted (RARP) and open radical prostatectomy (ORP) groups. These findings provide essential context for the interpretation of the machine learning model results presented in the main manuscript.

Key significant differences ($p < 0.05$):

- **Patient Age:** Patients in the RARP group were significantly older than those in the ORP group (median 67 vs. 65 years, p=0.002), particularly in the subgroup of patients who did not undergo nerve sparing (median 70 vs. 64 years, p<0.001).
- **Preoperative ISUP Grade:** The distribution of preoperative ISUP grades differed significantly between the groups (p=0.006), with a higher proportion of Grade 5 tumors in the open surgery cohort.
- **Perioperative Outcomes:** The RARP group was associated with significantly lower intraoperative blood loss, shorter catheter dwell times, shorter length of hospital stay, and lower postoperative hemoglobin drops (all p<0.001). These differences were consistent across all subgroups and demonstrated large effect sizes.
- **Pathological Outcomes:** Patients in the open surgery group had a significantly higher rate of positive surgical margins (R1 status) overall (40.0% vs. 22.9%, p=0.002).

Key non-significant findings of clinical interest:

- No significant baseline differences were observed between the RARP and ORP groups for key oncological risk parameters such as Initial PSA Value (p=0.845), D'Amico Risk Classification (p=0.311), or for patient characteristics like BMI (p=0.785) and ASA Score (p=0.703).
- The overall rate of postoperative complications, as graded by the Clavien-Dindo classification, did not differ significantly between the two surgical approaches (p=0.185). However, within the group of patients with minor complications, those in the ORP group had a significantly higher proportion of Grade II complications (p=0.009).

Rationale and Context for the Machine Learning Analysis

The detailed descriptive statistics presented in this supplement provide the essential context for interpreting the SHAP analysis in the main manuscript. The machine learning models learn to identify the most predictive features based on the statistical patterns and group differences in the training data. Therefore, this foundational analysis allows the reader to directly connect the model's feature importances (the "why" from the SHAP analysis) to the underlying, observable data patterns, thereby strengthening the clinical plausibility of the model's findings.

References

[1] Shapley LS (1953) *A Value for n-Person Games*, in: Kuhn HW, Tucker AW (eds) Contributions to the Theory of Games II, Annals of Mathematics Studies 28, Princeton University Press, 307–317

[2] Shapley LS (1967) *On balanced sets and cores*, Naval Research Logistics Quarterly 14(4), 453–460

[3] Lundberg SM, Lee SI (2017) *A Unified Approach to Interpreting Model Predictions*, Advances in Neural Information Processing Systems (NeurIPS) 30

[4] Castro J, Gómez D, Tejada J (2009) *Polynomial calculation of the Shapley value based on sampling*, Computers & Operations Research 36(5): 1726–1730
