# Supplementary material for: Explainable artificial intelligence reveals key surgical parameters in robot-assisted and open radical prostatectomy
Source: Sci Rep. 2026 Aug 26;16:26797. doi: 10.1038/s41598-026-68074-9 (PMC13507051; doi:10.1038/s41598-026-68074-9)
Supplement: Supplementary file 2 [file 41598_2026_68074_MOESM2_ESM.docx]

**Supplementary Material for:**

**Explainable Artificial Intelligence reveals key surgical parameters in robot-assisted and open radical prostatectomy**

Christian R. Klein^1, 5^*, Pia Heuser^3,5^, Elena Trunz^2^, Glen Kristiansen^4,5^, Peter Brossart^1,5^, Manuel Ritter^3,5^, Philipp Krausewitz^3,5^

**Institutional address:**

1: Clinic of Internal Medicine III, Oncology, Hematology, Immune-Oncology and Rheumatology, University Hospital, Bonn, Bonn, Germany

2: Institute for Computer Science, Department of Visual Computing, University of Bonn, Bonn, Germany

3: Department of Urology, University Hospital Bonn, Bonn, Germany

4: Institute of Pathology, University Hospital Bonn, Bonn, Germany

5: Center for Integrated Oncology Aachen Bonn Cologne Duesseldorf (CIO ABCD), Germany

***Correspondence:** Christian R. Klein (christian.klein@ukbonn.de), Department of Oncology, Hematology, Immune-Oncology and Rheumatology, University Hospital Bonn, Bonn, Germany, Tel. 015781684830, ORCID: 0009-0006-1306-7775

**Supplementary Tables**

| **ISUP** | Random forest | Boosting | SVM | **NN** | Mean | Median | **Ridge**  **regression** |
| --- | --- | --- | --- | --- | --- | --- | --- |
| MAE | 0.69 ± 0.05 | 0.68 ± 0.04 | 0.63 ± 0.03 | 0.64 ± 0.06 | 0.97 ± 0.08 | 0.97 ± 0.08 | 0.63 ± 0.06 |
| RMSE | 0.99 ± 0.05 | 0.98 ± 0.04 | 0.95 ± 0.04 | 0.94 ± 0.0 | 1.20 ± 0.05 | 1.20 ± 0.05 | 0.93 ± 0.05 |
| 100-MAPE*100 | 75.1% ± 1.1% | 75.6% ± 2.0% | 77.4% ± 2.1% | 76.5% ± 1.4% | 61.87% ± 4.87% | 61.87% ± 4.87% | 76.7% ± 1.5% |
| R2 | 30.2% ± 9.4% | 32.0% ± 10.9% | 36.0% ± 9.0% | 37.0% ± 7.4% | neg | neg | 38.6% ± 7.1% |
| **Length of Hospital Stay** | Random forest | Boosting | **SVM** | NN | Mean | Median | Ridge  regression |
| MAE | 1.10 ± 0.22 | 1.02 ± 0.21 | 0.94 ± 0.25 | 1.19 ± 0.21 | 2.01 ± 0.20 | 1.78 ± 0.35 | 1.19 ± 0.21 |
| RMSE | 2.12 ± 0.68 | 2.07 ± 0.67 | 1.95 ± 0.77 | 2.08 ± 0.71 | 2.73 ± 0.68 | 2.80 ± 0.85 | 2.08 ± 0.71 |
| 100-MAPE*100 | 86.3% ± 2.4% | 87.8% ± 1.5% | 89.0% ± 1.9% | 84.6% ± 1.6% | 72.6% ± 2.8% | 78.9% ± 1.7% | 84.6% ± 1.6% |
| R2 | 35.2% ± 12.9% | 38.8% ± 10.1% | 45.7% ± 17.9% | 37.7% ± 15.1% | neg | neg | 37.4% ± 15.3% |
| **Catheter Dwell Time** | **Random forest** | Boosting | SVM | NN | Mean | Median | Ridge  regression |
| MAE | 2.22 ± 0.45 | 1.82 ± 0.69 | 1.49 ± 0.49 | 2.68 ± 0.53 | 2.82 ± 0.48 | 2.30 ± 0.43 | 2.68 ± 0.53 |
| RMSE | 4.69 ± 1.39 | 4.81 ± 1.39 | 4.89 ± 1.47 | 4.83 ± 1.42 | 5.20 ± 1.44 | 5.65 ± 1.48 | 4.83 ± 1.41 |
| 100%-SMAPE | 89.1% ± 1.3% | 91.6% ± 3.1% | 93.9% ± 1.1% | 84.6% ± 2.1% | 83.3% ± 1.2% | 88.0% ± 1.0% | 84.6% ± 2.2% |
| R2 | 21.5% ± 20.0% | 14.1% ± 5.1% | 11.8% ± 6.4% | 15.8% ± 15.2% | neg | neg | 15.8% ± 15.0% |
| **Hb Drop** | Random forest | Boosting | SVM | **NN** | Mean | Median | **Ridge**  **regression** |
| MAE | 0.85 ± 0.04 | 0.87 ± 0.06 | 0.83 ± 0.06 | 0.83 ± 0.05 | 1.20 ± 0.11 | 1.17 ± 0.14 | 0.84 ± 0.05 |
| RMSE | 1.08 ± 0.07 | 1.11 ± 0.08 | 1.08 ± 0.08 | 1.06 ± 0.07 | 1.51 ± 0.14 | 1.52 ± 0.18 | 1.06 ± 0.07 |
| 100%-SMAPE | 82.9% ± 1.3% | 82.6% ± 1.4% | 83.4% ± 2.0% | 83.3% ± 1.9% | 77.5% ± 1.5% | 77.9% ± 1.8% | 83.2% ± 1.9% |
| R2 | 46.1% ± 3.6% | 43.1% ± 6.7% | 46.1% ± 3.9% | 47.6% ± 6.7% | neg | neg | 47.5% ± 6.6% |
| **Prostate Weight** | Random forest | Boosting | **SVM** | **NN** | Mean | Median | **Ridge**  **regression** |
| MAE | 12.66 ± 0.80 | 12.78 ± 0.90 | 12.42 ± 0.52 | 12.98 ± 0.76 | 17.11 ± 1.95 | 16.37 ± 2.40 | 12.96 ± 0.75 |
| RMSE | 17.83 ± 1.68 | 18.33 ± 1.86 | 18.15 ± 2.12 | 18.03 ± 1.44 | 24.09 ± 5.30 | 24.67 ± 5.65 | 18.02 ± 1.44 |
| 100-MAPE*100 | 70.3% ± 10.1% | 70.9% ± 10.2% | 72.7% ± 7.9% | 70.2% ± 7.5% | 57.6% ± 15.1% | 63.5% ± 14.5% | 70.2% ± 7.5% |
| R2 | 38.8% ± 20.8% | 35.8% ± 19.5% | 39.0% ± 20.5% | 40.3% ± 22.8% | neg | neg | 40.4% ± 22.7% |
| **Lymph Node Count** | Random forest | **Boosting** | SVM | NN | Mean | Median | Ridge  regression |
| MAE | 5.60 ± 0.72 | 5.31 ± 0.66 | 5.40 ± 0.73 | 5.43 ± 0.77 | 6.86 ± 0.56 | 6.69 ± 0.74 | 5.43 ± 0.77 |
| RMSE | 7.56 ± 1.19 | 7.32 ± 1.17 | 7.47 ± 1.41 | 7.45 ± 1.17 | 8.93 ± 1.20 | 9.12 ± 1.43 | 7.44 ± 1.17 |
| 100%-SMAPE | 79.4% ± 1.9% | 80.3% ± 1.7% | 80.0% ± 1.9% | 80.2% ± 2.2% | 75.1% ± 1.0% | 75.4% ± 1.5% | 80.1% ± 2.1% |
| R2 | 26.1% ± 11.1% | 31.4% ± 6.4% | 29.2% ± 8.6% | 28.4% ± 9.7% | neg | neg | 28.8% ± 9.4% |
| **Blood Loss** | Random forest | Boosting | **SVM** | NN | Mean | Median | Ridge  regression |
| MAE | 324 ± 43 | 276 ± 60 | 277 ± 51 | 303 ± 45 | 395 ± 41 | 355 ± 60 | 303 ± 45 |
| RMSE | 499 ± 86 | 455 ± 128 | 440 ± 110 | 459 ± 101 | 555 ± 119 | 575 ± 139 | 459 ± 101 |
| 100-MAPE*100 | 30.2% ± 9.5% | 47.7% ± 7.1% | 44.2% ± 5.6% | 35.0% ± 8.3% | 3.3% ± 16.4% | 34.1% ± 9.2% | 35.1% ± 8.3% |
| R2 | 17.8% ± 13.9% | 31.9% ± 13.5% | 35.8% ± 10.3% | 29.1% ± 17.6% | neg | neg | 29.2% ± 17.7% |
| **Intraop. Fluid Admin.** | Random forest | Boosting | **SVM** | NN | Mean | Median | Ridge  regression |
| MAE | 898 ± 125 | 861 ± 70 | 844 ± 67 | 910 ± 97 | 956 ± 86 | 894 ± 60 | 906 ± 94 |
| RMSE | 1243 ± 161 | 1196 ± 124 | 1186 ± 130 | 1247 ± 189 | 1281 ± 115 | 1298 ± 108 | 1243 ± 185 |
| 100-MAPE*100 | 62.4% ± 6.0% | 65.0% ± 4.5% | 65.8% ± 5.1% | 61.3% ± 3.4% | 58.7% ± 7.1% | 64.4% ± 4.9% | 61.5% ± 3.3% |
| R2 | 9.6% ± 9.4% | 11.8% ± 3.6% | 13.2% ± 7.5% | 11.5% ± 10.3% | neg | neg | 11.7% ± 10.2% |
|  |  |  |  |  |  |  |  |
| **Postop.Hct.** | Random  forest | Boosting | **SVM** | **NN** | Mean | Median | **Ridge**  **regression** |
| MAE | 2.57 ± 0.13 | 2.50 ± 0.17 | 2.39 ± 0.19 | 2.40 ± 0.20 | 3.93 ± 0.53 | 3.87 ± 0.55 | 2.40 ± 0.20 |
| RMSE | 3.26 ± 0.21 | 3.20 ± 0.23 | 3.08 ± 0.23 | 3.07 ± 0.26 | 4.75 ± 0.57 | 4.75 ± 0.60 | 3.07 ± 0.26 |
| 100-MAPE*100 | 92.2% ± 0.6% | 92.4% ± 0.6% | 92.7% ± 0.4% | 92.7% ± 0.5% | 87.9% ± 1.9% | 88.0% ± 2.0% | 92.7% ± 0.5% |
| R2 | 51.5% ± 7.1% | 53.3% ± 6.6% | 55.1% ± 12.7% | 55.5% ± 13.3% | neg | neg | 55.5% ± 13.3% |
| **Postop.Hb.** | Random  forest | Boosting | SVM | **NN** | Mean | Median | **Ridge**  **regression** |
| MAE | 0.88 ± 0.02 | 0.87 ± 0.03 | 0.85 ± 0.06 | 0.85 ± 0.05 | 1.40 ± 0.16 | 1.38 ± 0.15 | 0.85 ± 0.05 |
| RMSE | 1.13 ± 0.04 | 1.13 ± 0.06 | 1.11 ± 0.09 | 1.08 ± 0.07 | 1.70 ± 0.15 | 1.72 ± 0.15 | 1.08 ± 0.07 |
| 100-MAPE*100 | 92.3% ± 0.3% | 92.3% ± 0.5% | 92.4% ± 0.5% | 92.5% ± 0.3% | 87.5% ± 1.6% | 87.4% ± 1.5% | 92.5% ± 0.3% |
| R2 | 54.5% ± 8.8% | 54.5% ± 8.0% | 55.4% ± 12.2% | 57.5% ± 11.4% | neg | neg | 57.5% ± 11.5% |

**(A) Regression Tasks**

| **Frozen Section Result** | Random forest | Boosting | SVM | **NN** | Mode | Logistic  regression |
| --- | --- | --- | --- | --- | --- | --- |
| Accuracy | 77.0% ± 4.0% | 76.6% ± 3.5% | 73.8% ± 3.7% | 76.0% ± 5.4% | 50.5% ± 4.4% | 73.8% ± 6.4% |
| AUC | 78.1% ± 3.0% | 78.6% ± 4.8% | 78.0% ± 3.6% | 80.3% ± 0.9% | N/A | 77.2% ± 4.4% |
| Sensitivity | 60.3% ± 9.1% | 61.0% ± 8.0% | 58.1% ± 8.3% | 54.2% ± 3.2% | 36.7% ± 6.7% | 52.9% ± 4.8% |
| Specificity | 84.3% ± 4.0% | 83.9% ± 4.2% | 82.4% ± 4.5% | 85.4% ± 2.9% | 63.3% ± 6.7% | 84.4% ± 3.5% |
| PPV | 56.9% ± 10.6% | 63.3% ± 13.8% | 55.0% ± 9.6% | 51.5% ± 3.7% | 18.7% ± 5.0% | 50.3% ± 3.8% |
| NPV | 85.7% ± 4.8% | 85.5% ± 4.5% | 83.8% ± 5.5% | 86.6% ± 3.2% | 45.4% ± 8.5% | 85.2% ± 4.0% |
| **FS Decision (yes/no)** | **Random forest** | Boosting | SVM | NN | Mode | Logistic  regression |
| Accuracy | 80.1% ± 4.3% | 80.4% ± 6.2% | 78.2% ± 5.0% | 79.5% ± 2.9% | 46.7% ± 3.0% | 78.5% ± 7.3% |
| AUC | 89.2% ± 2.9% | 87.4% ± 4.4% | 84.4% ± 5.9% | 87.8% ± 2.8% | N/A | 83.8% ± 5.7% |
| Sensitivity | 80.1% ± 10.0% | 83.1% ± 8.5% | 78.5% ± 12.8% | 80.6% ± 9.2% | 60.0% ± 49.0% | 74.5% ± 11.2% |
| Specificity | 81.2% ± 9.2% | 78.2% ± 8.7% | 79.4% ± 8.6% | 79.2% ± 4.5% | 40.0% ± 49.0% | 83.1% ± 5.4% |
| PPV | 81.9% ± 7.6% | 80.0% ± 6.6% | 79.9% ± 6.3% | 79.8% ± 4.0% | 28.6% ± 23.4% | 82.0% ± 4.0% |
| NPV | 80.2% ± 9.8% | 81.5% ± 10.4% | 79.0% ± 12.2% | 80.1% ± 9.2% | 18.1% ± 22.2% | 75.9% ± 11.2% |

**(B) Classification Tasks**

**Supplementary Table S1: Performance metrics for machine learning models in predicting postoperative outcomes.** The table summarizes the predictive performance of four machine learning models (Random Forest, Gradient Boosting, Support Vector Machine [SVM], and Neural Network [NN]) compared to heuristic baseline models (Mean/Median for regression, Mode for classification). Performance was evaluated using a 5-fold cross-validation scheme. **(A) Regression Tasks:** Model performance for predicting continuous target parameters was assessed using the Mean Absolute Error (MAE), Root Mean Squared Error (RMSE), a relative accuracy metric derived from the Mean Absolute Percentage Error (100% - MAPE), and the coefficient of determination (R²). For outcomes where true target values include zero, MAPE is mathematically undefined (division by zero); for these outcomes, the MAPE rows are omitted and replaced by SMAPE-based Accuracy (100% − SMAPE). Standard SMAPE is an error metric for which 0% indicates perfect prediction and higher values indicate greater error; after transformation, 100% indicates perfect prediction and lower values indicate greater prediction error. Lower MAE and RMSE values indicate better performance, while higher relative accuracy and R² values indicate a better model fit. An R² value of 'neg.' indicates that the model performed worse than simply predicting the mean of the target variable. This can occur because the R² is calculated on the hold-out test set, whereas the baseline mean is derived solely from the training set. **(B) Classification Tasks:** Model performance for predicting categorical outcomes was assessed using Accuracy, Area Under the ROC Curve (AUC), Sensitivity, Specificity, Positive Predictive Value (PPV), and Negative Predictive Value (NPV). For the Frozen Section Result outcome, macro-averaged one-vs-rest metrics are reported to account for the multiclass structure. Higher values indicate better performance for all metrics. **Abbreviations:** AUC, Area Under the ROC Curve; FS, Frozen Section; MAE, Mean Absolute Error; NN, Neural Network; NPV, Negative Predictive Value; PPV, Positive Predictive Value; R², Coefficient of Determination; RMSE, Root Mean Squared Error; SMAPE, Symmetric Mean Absolute Percentage Error; SVM, Support Vector Machine.

| **Category** | **Parameter** | **Description** | **Values / Scale** |
| --- | --- | --- | --- |
| **Surgical Complications** | Paravasation | Anastomotic leakage | yes/no, nominal |
|  | Urinoma | Anastomotic leakage | yes/no, nominal |
|  | Lymphocele |  | yes/no, nominal |
| **Operative Complications** | Clavien-Dindo | Up to 30 days post-op | 1-5, ordinal |
|  | CD_what | Coding of the specific complication | Nominal |
|  | CD_simplified | Up to 30 days post-op, minor/major | 0-2, ordinal |
|  | CD_admission | Type of follow-up treatment | 0-3, nominal |
|  | CD_procedure | Type of re-intervention | Nominal |
| **Pathological Parameters** | FS_decision | Frozen section performed? | yes/no, nominal |
|  | FS_result | Composite FS utilization/result category (no FS/positive/negative) | 0-2, nominal |
|  | Prostate_weight | Macroscopic weight of the specimen | [g], metric |
|  | Tumor_size | Maximum tumor extension | [mm], metric |
|  | Postop_pT | Postoperative pathological T-stage |  |
|  | pT_stage_bin | pT1-2 vs. pT3-4 | nominal |
|  | Pn_status | Perineural invasion |  |
|  | V_status | Venous invasion |  |
|  | L_status | Lymphatic invasion |  |
|  | R_status | Resection margin status (R0/R1) |  |
|  | R1_Gleason | Gleason score at positive margin |  |
|  | R1_extent | Extent of positive margin |  |
|  | N_positive | Number of positive lymph nodes |  |
|  | N_status_positive | Any positive lymph nodes |  |
|  | N_status_harvested | Number of harvested lymph nodes |  |
|  | ISUP_postop | Postoperative ISUP Grade |  |
| **Procedural Metrics** | Skin_to_skin_time | Total surgical time | [min], metric |
|  | Console_time | Console time (RARP only) | [min], metric |
|  | LoHS | Length of Hospital Stay | [days], metric |
|  | Catheter_dwell_time | Duration of indwelling urinary catheter | [days], metric |
| **Physiological Parameters** | Blood_loss | Intraoperative blood loss | [ml], metric |
|  | Fluid_admin_intraop | Intraoperative fluid administration | [ml], metric |
|  | Blood_transfusion | Postoperative RBC transfusion required | yes/no, nominal |
|  | postop_Hct | Hematocrit post-op | [%], metric |
|  | postop_Hb | Hemoglobin post-op | [g/dl], metric |
|  | Hb_drop | Pre- to postoperative Hb drop | [g/dl], metric |
| **Urological Follow-up** | Continence_12m | Continence status after 12 months | yes/no, nominal |
|  | Continence_quant | Pad usage per day after 12 months | ordinal |
|  | Erectile_dysfunction | Potency at 12 months | yes/no/not used, nominal |
|  | PSA_3m | PSA level after 3 months | [ng/ml], metric |
|  | Tumor_board_rec | Recommended procedure | 1-5, nominal |
| **Sexual Function Parameters** | Desire_for_intercourse | Desire for sexual intercourse? | yes/no/not recorded, nominal |
|  | Intercourse_function | Sexual intercourse functional at 12m | yes/no, nominal |
|  | Intercourse_aids | Use of aids (PDE5i, VED, etc.) | nominal |
|  | Success_of_aids | Were the aids helpful? | yes/no, nominal |

**Supplementary Table S2: Target Outcome Variables.** The table provides a comprehensive list and description of all intra- and postoperative parameters that were defined as target outcomes for the machine learning models. **Abbreviations and Definitions:** **CD (Clavien-Dindo):** Refers to the Clavien-Dindo classification of surgical complications. **CD_admission:** Classification of the required follow-up treatment for complications, categorized as: {no treatment, outpatient treatment, inpatient admission (>48h after discharge), or treatment during initial surgical stay / re-admission within 48h}. **CD_procedure:** Classification of the necessary intervention performed for complication management. Interventions in this study included: {Application of anti-inflammatory medication (corticosteroids and antihistamines), Antibiotic therapy, Catheter placement, Cystography, Delirium management, Drainage of lymphocele, Fascia repair (Mayo Technique), Interventional radiological hemostasis, No intervention, Operative exploration, Placement of Mono-J catheter, Revision for wound dehiscence, Robotic-assisted hematoma evacuation, Robotic-assisted revision, Transfusion of red blood cells, Urinary catheter placement}. **CD_simplified:** A binary categorization of postoperative complications recorded up to 30 days after surgery as ‘minor’ (Clavien-Dindo classification I–IIIa) or ‘major’ (Clavien-Dindo classification IIIb–IVa). **CD_what:** The specific type of surgical complication. Complications observed in this study were: {Acute kidney injury, Allergic skin reaction, Anastomotic insufficiency, Death, Diarrhea, Hematoma, Hernia, Lymphocele, Musculoskeletal pain (position-related), NSTEMI (Non-ST Elevation Myocardial Infarction), Pleural effusion, Postoperative infection, Reoperation, Scrotal swelling, Surgical wound dehiscence, Thrombosis, Transfusion, Urinary retention, Urinoma, Upper respiratory tract infection, Urinary obstruction}. **FS:** Frozen Section. **ISUP:** International Society of Urological Pathology Grade. **LoHS:** Length of Hospital Stay. **PDE5i:** Phosphodiesterase type 5 inhibitors. **RBC:** Red Blood Cell. **RPCS:** Radical Prostatectomy Complication Score.**VED:** Vacuum Erection Device. **Note on FS Result:** The variable FS_result is a composite three-class outcome encoding both the utilization decision and the pathological result: Class 0 = frozen section not performed; Class 1 = frozen section performed with a positive result; Class 2 = frozen section performed with a negative result. This variable therefore does not represent the pathological result restricted to patients in whom frozen section was performed, but rather integrates both utilization and result in a single composite variable.

| **Parameter** | **Blood Loss** | **Hb Drop** | **postop. Hb** | **postop. Hct** | **ISUP** | **Catheter Dwell Time** | **Length of Hospital Stay** | **Lymph Node Count** | **Prostate Weight** | **Frozen Section** | **FS Decision** | **Intraop. Fluid Admin.** |
| --- | --- | --- | --- | --- | --- | --- | --- | --- | --- | --- | --- | --- |
| RARP | 2711060 | 19263,96 | 18844,47 | 52765,71 | 468,1617 | 25397,2 | 33546,99 | 63760,36 | 48466,54 | 50,42975 | 220,4052 | 6077935 |
| NS (any) | 84852,23 | 8539,145 | 7242,981 | 17626,27 | 5284,992 | 720,076 | 58615,37 | 4247 | 101463,8 | 113,2682 | 483,3855 | 1981930 |
| preop. Hb | 288228 | 7306,648 | 7023,792 | 10389,65 | 415,0153 | 476,6135 | 508,1619 | 9487,033 | 19508,18 | 48,74961 | 260,2342 | 384705,1 |
| cT-stage (preop.) | 148025,9 | 5461,76 | 5583,581 | 13153,59 | 3296,849 | 1009,986 | 3315,928 | 1597,958 | 45729,63 | 17,10343 | 58,23823 | 2022561 |
| NS Extent | 72657,7 | 3937,557 | 4323,984 | 10990,75 | 2984,605 | 529,3466 | 33024,74 | 2800,727 | 56794,72 | 51,13649 | 194,9641 | 1680405 |
| Suspicious DRE | 70047,06 | 3021,211 | 3200,363 | 6167,056 | 1889,559 | 226,2134 | 698,2829 | 698,6019 | 14305,42 | 15,28086 | 55,73608 | 350994,4 |
| Biopsy GS | 62501,22 | 2591,565 | 1639,269 | 5009,264 | 4465,727 | 496,8456 | 2166,762 | 9787,051 | 18348,86 | 20,29768 | 76,85381 | 394789,6 |
| ASA Score | 35900,8 | 1920,074 | 1342,62 | 3937,302 | 256,5718 | 83,37886 | 204,0189 | 2346,736 | 9315,262 | 22,56685 | 84,14829 | 352170,2 |
| preop. Hct | 286298,1 | 1792,863 | 5187,846 | 23842,5 | 178,966 | 181,2208 | 644,2144 | 4419,058 | 15941,07 | 32,84495 | 148,6771 | 1321816 |
| ISUP Grade (preop.) | 29941,04 | 1555,773 | 1801,831 | 3544,402 | 5636,688 | 671,782 | 2401,85 | 6297,668 | 5966,275 | 23,09921 | 99,56689 | 346533,4 |
| D'Amico Score | 52919,13 | 1504,417 | 987,5568 | 2652,012 | 2842,648 | 611,6226 | 2101,863 | 15892,86 | 44644,67 | 16,33533 | 65,6501 | 1543067 |
| Prior Therapy | 17934,69 | 1268,486 | 2326,781 | 5088,416 | 225,2753 | 310,3443 | 513,8971 | 227,8632 | 22524,86 | 14,11167 | 59,20068 | 343879,5 |
| preop. ADT | 3024,807 | 956,4536 | 2366,204 | 3857,295 | 223,5938 | 206,2307 | 771,0091 | 166,0109 | 9558,731 | 8,804619 | 37,15474 | 417079,2 |
| preop. Prostate Volume | 345941,7 | 627,8722 | 788,5131 | 1975,117 | 517,3078 | 115,7585 | 490,1655 | 6752,358 | 183138,2 | 36,28056 | 128,5828 | 748034,4 |
| Prior Therapy Type | 6448,333 | 617,7105 | 1500,115 | 2564,994 | 141,0861 | 157,022 | 314,9469 | 39,01567 | 11314,92 | 5,491689 | 30,36519 | 214845,4 |
| Age | 340385,3 | 558,7107 | 735,6604 | 2340,501 | 863,6116 | 74,11118 | 532,5917 | 7458,282 | 10679,5 | 30,92894 | 117,4492 | 993101,7 |
| BMI | 537700,7 | 495,9443 | 1123,11 | 2116,525 | 207,7815 | 209,6923 | 570,0504 | 10327,27 | 18191,91 | 29,14057 | 107,3942 | 355714,8 |
| Initial PSA | 427898,7 | 298,0352 | 700,7462 | 1000,81 | 746,9333 | 1012,366 | 2133,817 | 13653,07 | 11764,96 | 30,14217 | 115,345 | 747144,6 |

**Supplementary Table S3: Unnormalized Mean Absolute SHAP Values for All Predictive Models.** The table displays the raw, unnormalized mean absolute SHAP (SHapley Additive exPlanations) values for each preoperative input feature across all twelve predictive target outcomes. These values represent the total aggregated impact of each feature on the model's predictions for the respective outcome. It is important to note that these SHAP values are unitless in this aggregated form and do not represent physical units (e.g., ml of blood loss or days of hospital stay). Instead, they quantify the magnitude of a feature's contribution to the model's prediction function across the entire dataset. For direct interpretability and comparison between different outcomes, the values from this table were normalized to sum to 100% to generate the relative feature importance plots in Figure 4 of the main manuscript.

| **ISUP** | Random forest | Boosting | SVM | **NN** | Mean | Median | **Ridge**  **regression** |
| --- | --- | --- | --- | --- | --- | --- | --- |
| MAE | 0.72 ± 0.07 | 0.68 ± 0.06 | 0.65 ± 0.06 | 0.65 ± 0.05 | 0.97 ± 0.08 | 0.97 ± 0.08 | 0.65 ± 0.07 |
| RMSE | 1.01 ± 0.06 | 0.99 ± 0.06 | 0.96 ± 0.05 | 0.95 ± 0.04 | 1.20 ± 0.05 | 1.20 ± 0.05 | 0.95 ± 0.05 |
| 100-MAPE*100 | 73.9% ± 0.9% | 76.1% ± 2.8% | 76.8% ± 0.9% | 75.5% ± 0.8% | 61.87% ± 4.87% | 61.87% ± 4.87% | 75.7% ± 1.3% |
| R2 | 28.0% ± 9.1% | 30.5% ± 13.9% | 34.2% ± 10.4% | 36.2% ± 8.7% | neg | neg | 36.4% ± 9.6% |
| **Length of Hospital Stay** | Random forest | Boosting | **SVM** | NN | Mean | Median | Ridge  regression |
| MAE | 1.14 ± 0.20 | 1.04 ± 0.27 | 0.94 ± 0.26 | 1.17 ± 0.24 | 2.01 ± 0.20 | 1.78 ± 0.35 | 1.17 ± 0.24 |
| RMSE | 2.10 ± 0.66 | 2.08 ± 0.73 | 1.96 ± 0.77 | 2.08 ± 0.72 | 2.73 ± 0.68 | 2.80 ± 0.85 | 2.08 ± 0.72 |
| 100-MAPE*100 | 85.7% ± 2.2% | 87.5% ± 1.8% | 88.9% ± 1.9% | 85.0% ± 1.8% | 72.6% ± 2.8% | 78.9% ± 1.7% | 85.0% ± 1.8% |
| R2 | 36.9% ± 10.8% | 38.8% ± 11.8% | 45.0% ± 18.3% | 37.8% ± 16.1% | neg | neg | 37.8% ± 16.1% |
| **Catheter Dwell Time** | **Random forest** | Boosting | SVM | NN | Mean | Median | Ridge  regression |
| MAE | 2.21 ± 0.49 | 1.80 ± 0.68 | 1.49 ± 0.49 | 2.62 ± 0.58 | 2.82 ± 0.48 | 2.30 ± 0.43 | 2.62 ± 0.58 |
| RMSE | 4.72 ± 1.42 | 4.72 ± 1.37 | 4.89 ± 1.47 | 4.77 ± 1.45 | 5.20 ± 1.44 | 5.65 ± 1.48 | 4.77 ± 1.45 |
| 100%-SMAPE | 89.2% ± 1.6% | 91.5% ± 3.1% | 93.9% ± 1.1% | 85.4% ± 2.6% | 83.3% ± 1.2% | 88.0% ± 1.0% | 85.4% ± 2.6% |
| R2 | 21.1% ± 21.5% | 16.7% ± 7.9% | 11.5% ± 5.9% | 17.2% ± 16.7% | neg | neg | 17.2% ± 16.7% |
| **Hb Drop** | Random forest | **Boosting** | SVM | **NN** | Mean | Median | **Ridge**  **regression** |
| MAE | 0.86 ± 0.04 | 0.85 ± 0.05 | 0.86 ± 0.08 | 0.85 ± 0.05 | 1.20 ± 0.11 | 1.17 ± 0.14 | 0.85 ± 0.05 |
| RMSE | 1.09 ± 0.07 | 1.07 ± 0.06 | 1.11 ± 0.09 | 1.07 ± 0.07 | 1.51 ± 0.14 | 1.52 ± 0.18 | 1.07 ± 0.07 |
| 100%-SMAPE | 82.7% ± 1.6% | 83.0% ± 1.4% | 82.9% ± 2.3% | 83.0% ± 1.9% | 77.5% ± 1.5% | 77.9% ± 1.8% | 82.9% ± 1.9% |
| R2 | 45.0% ± 3.9% | 46.7% ± 6.0% | 43.0% ± 6.7% | 46.9% ± 7.5% | neg | neg | 46.8% ± 7.5% |
| **Prostate Weight** | Random forest | Boosting | **SVM** | **NN** | Mean | Median | **Ridge**  **regression** |
| MAE | 13.08 ± 0.87 | 13.20 ± 0.85 | 12.65 ± 0.65 | 13.14 ± 1.27 | 17.11 ± 1.95 | 16.37 ± 2.40 | 13.12 ± 1.25 |
| RMSE | 18.26 ± 1.72 | 18.37 ± 1.78 | 18.13 ± 1.34 | 18.37 ± 1.67 | 24.09 ± 5.30 | 24.67 ± 5.65 | 18.35 ± 1.66 |
| 100-MAPE*100 | 68.8% ± 11.1% | 68.5% ± 12.0% | 71.2% ± 8.2% | 69.3% ± 7.6% | 57.6% ± 15.1% | 63.5% ± 14.5% | 69.3% ± 7.6% |
| R2 | 36.4% ± 20.3% | 35.8% ± 17.8% | 38.0% ± 21.5% | 38.8% ± 23.4% | neg | neg | 38.8% ± 23.4% |
| **Lymph Node Count** | Random forest | Boosting | **SVM** | NN | Mean | Median | Ridge  regression |
| MAE | 5.57 ± 0.73 | 5.39 ± 0.72 | 5.36 ± 0.81 | 5.40 ± 0.76 | 6.86 ± 0.56 | 6.69 ± 0.74 | 5.40 ± 0.76 |
| RMSE | 7.58 ± 1.21 | 7.42 ± 1.20 | 7.39 ± 1.42 | 7.39 ± 1.21 | 8.93 ± 1.20 | 9.12 ± 1.43 | 7.39 ± 1.22 |
| 100%-SMAPE | 79.6% ± 2.0% | 80.0% ± 1.9% | 80.2% ± 2.1% | 80.1% ± 2.0% | 75.1% ± 1.0% | 75.4% ± 1.5% | 80.1% ± 2.0% |
| R2 | 26.1% ± 9.8% | 29.6% ± 6.8% | 30.7% ± 8.6% | 29.8% ± 9.5% | neg | neg | 29.8% ± 9.5% |
| **Blood Loss** | Random forest | Boosting | **SVM** | NN | Mean | Median | Ridge  regression |
| MAE | 324 ± 38 | 273 ± 59 | 278 ± 51 | 299 ± 41 | 395 ± 41 | 355 ± 60 | 298 ± 42 |
| RMSE | 499 ± 81 | 447 ± 123 | 441 ± 111 | 455 ± 98 | 555 ± 119 | 575 ± 139 | 454 ± 98 |
| 100-MAPE*100 | 30.8% ± 9.8% | 47.7% ± 7.1% | 44.5% ± 5.2% | 36.1% ± 7.6% | 3.3% ± 16.4% | 34.1% ± 9.2% | 36.2% ± 7.5% |
| R2 | 17.5% ± 11.9% | 31.9% ± 13.5% | 35.6% ± 10.7% | 30.0% ± 17.4% | neg | neg | 30.2% ± 17.5% |
| **Intraop. Fluid Admin.** | Random forest | Boosting | **SVM** | NN | Mean | Median | Ridge  regression |
| MAE | 923 ± 127 | 857 ± 86 | 852 ± 58 | 897 ± 84 | 956 ± 86 | 894 ± 60 | 895 ± 83 |
| RMSE | 1260 ± 159 | 1203 ± 147 | 1190 ± 126 | 1236 ± 175 | 1281 ± 115 | 1298 ± 108 | 1233 ± 17 |
| 100-MAPE*100 | 61.5% ± 6.3% | 65.1% ± 5.1% | 65.5% ± 5.0% | 61.8% ± 3.6% | 58.7% ± 7.1% | 64.4% ± 4.9% | 61.9% ± 3.6% |
| R2 | 7.3% ± 9.1% | 11.0% ± 6.9% | 12.6% ± 6.5% | 11.5% ± 9.8% | neg | neg | 11.7% ± 9.7% |
|  |  |  |  |  |  |  |  |
| **Postop.Hct.** | Random  forest | Boosting | **SVM** | **NN** | Mean | Median | **Ridge**  **regression** |
| MAE | 2.57 ± 0.16 | 2.52 ± 0.15 | 2.43 ± 0.22 | 2.43 ± 0.20 | 3.93 ± 0.53 | 3.87 ± 0.55 | 2.42 ± 0.20 |
| RMSE | 3.26 ± 0.25 | 3.19 ± 0.20 | 3.11 ± 0.18 | 3.07 ± 0.21 | 4.75 ± 0.57 | 4.75 ± 0.60 | 3.07 ± 0.21 |
| 100-MAPE*100 | 92.2% ± 0.7% | 92.3% ± 0.6% | 92.5% ± 0.5% | 92.6% ± 0.5% | 87.9% ± 1.9% | 88.0% ± 2.0% | 92.6% ± 0.5% |
| R2 | 51.4% ± 7.6% | 53.4% ± 7.0% | 55.0% ± 10.3% | 56.0% ± 11.8% | neg | neg | 56.0% ± 11.8% |
| **Postop.Hb.** | Random  forest | Boosting | SVM | **NN** | Mean | Median | **Ridge**  **regression** |
| MAE | 0.88 ± 0.01 | 0.88 ± 0.03 | 0.88 ± 0.05 | 0.86 ± 0.05 | 1.40 ± 0.16 | 1.38 ± 0.15 | 0.86 ± 0.05 |
| RMSE | 1.13 ± 0.04 | 1.14 ± 0.06 | 1.13 ± 0.04 | 1.08 ± 0.05 | 1.70 ± 0.15 | 1.72 ± 0.15 | 1.08 ± 0.05 |
| 100-MAPE*100 | 92.3% ± 0.2% | 92.2% ± 0.4% | 92.2% ± 0.3% | 92.4% ± 0.3% | 87.5% ± 1.6% | 87.4% ± 1.5% | 92.4% ± 0.3% |
| R2 | 54.7% ± 9.0% | 53.9% ± 8.5% | 54.1% ± 9.9% | 57.7% ± 10.0% | neg | neg | 57.6% ± 10.1% |

**(A) Regression Tasks**

| **Frozen Section Result** | Random forest | Boosting | SVM | **NN** | Mode | **Logistic**  **regression** |
| --- | --- | --- | --- | --- | --- | --- |
| Accuracy | 59.6% ± 7.2% | 58.7% ± 3.8% | 58.4% ± 8.3% | 59.6% ± 4.1% | 50.5% ± 4.4% | 59.6% ± 5.6% |
| AUC | 62.7% ± 8.8% | 62.3% ± 5.2% | 61.9% ± 6.1% | 63.0% ± 6.8% | N/A | 65.6% ± 6.5% |
| Sensitivity | 46.4% ± 7.3% | 41.6% ± 3.9% | 46.2% ± 12.0% | 46.3% ± 6.9% | 36.7% ± 6.7% | 47.7% ± 8.3% |
| Specificity | 71.9% ± 8.9% | 74.3% ± 3.2% | 72.2% ± 4.6% | 71.9% ± 7.1% | 63.3% ± 6.7% | 72.3% ± 6.9% |
| PPV | 44.0% ± 8.2% | 40.7% ± 4.0% | 43.9% ± 12.8% | 43.9% ± 8.3% | 18.7% ± 5.0% | 51.0% ± 15.9% |
| NPV | 73.0% ± 9.8% | 75.5% ± 3.9% | 73.0% ± 5.2% | 72.6% ± 7.3% | 45.4% ± 8.5% | 72.9% ± 7.1% |
| **FS Decision (yes/no)** | **Random forest** | Boosting | SVM | NN | Mode | Logistic  regression |
| Accuracy | 64.1% ± 8.5% | 63.4% ± 7.3% | 62.8% ± 6.3% | 63.7% ± 4.6% | 46.7% ± 3.0% | 63.4% ± 4.6% |
| AUC | 69.4% ± 8.9% | 67.8% ± 10.3% | 65.4% ± 8.1% | 68.5% ± 6.4% | N/A | 68.3% ± 7.4% |
| Sensitivity | 65.4% ± 13.8% | 68.9% ± 11.8% | 66.8% ± 10.1% | 65.6% ± 8.4% | 60.0% ± 49.0% | 61.7% ± 12.4% |
| Specificity | 63.8% ± 9.0% | 58.8% ± 10.8% | 59.7% ± 12.7% | 62.5% ± 7.4% | 40.0% ± 49.0% | 65.5% ± 12.0% |
| PPV | 64.7% ± 7.7% | 63.3% ± 7.5% | 63.4% ± 8.2% | 64.2% ± 5.7% | 28.6% ± 23.4% | 65.5% ± 6.5% |
| NPV | 64.9% ± 13.8% | 65.5% ± 13.1% | 63.7% ± 9.8% | 64.1% ± 9.5% | 18.1% ± 22.2% | 63.1% ± 8.5% |
| **Nerve sparing** | **Random forest** | **Boosting** | **SVM** | **NN** | Mode | **Logistic**  **regression** |
| Accuracy | 70.2% ± 5.2% | 69.5% ± 5.2% | 69.5% ± 5.2% | 70.8% ± 5.9% | 56.9% ± 7.5% | 71.4% ± 6.0% |
| AUC | 79.9% ± 6.0% | 80.3% ± 3.6% | 79.1% ± 6.4% | 78.6% ± 2.6% | N/A | 79.5% ± 3.8% |
| Sensitivity | 60.5% ± 8.1% | 60.9% ± 11.3% | 61.5% ± 9.8% | 61.4% ± 13.9% | 0.0% ± 0.0% | 59.5% ± 7.5% |
| Specificity | 78.9% ± 9.2% | 78.0% ± 8.3% | 77.7% ± 9.6% | 80.0% ± 6.5% | 100.0% ± 0.0% | 81.8% ± 9.8% |
| PPV | 69.1% ± 12.0% | 67.7% ± 10.8% | 67.9% ± 12.4% | 69.6% ± 8.9% | 0.0% ± 0.0% | 71.7% ± 13.5% |
| NPV | 72.1% ± 9.0% | 72.3% ± 10.2% | 72.4% ± 9.2% | 73.0% ± 11.6% | 56.9% ± 7.5% | 72.4% ± 8.4% |

**(B) Classification Tasks**

**Supplementary Table S4: Performance metrics — full cohort, without nerve-sparing parameters as input features.** The table summarizes the predictive performance of four machine learning models (Random Forest, Gradient Boosting, Support Vector Machine [SVM], and Neural Network [NN]) compared to heuristic baseline models (Mean/Median for regression, Mode for classification). Performance was evaluated using a 5-fold cross-validation scheme. **(A) Regression Tasks:** Model performance for predicting continuous target parameters was assessed using the Mean Absolute Error (MAE), Root Mean Squared Error (RMSE), relative accuracy metrics derived from the Mean Absolute Percentage Error (100% - MAPE), or SMAPE-based Accuracy (100% − SMAPE) for outcomes containing zero values, and the coefficient of determination (R²). Lower MAE and RMSE values indicate better performance, while higher relative accuracy and R² values indicate a better model fit. An R² value of 'neg.' indicates that the model performed worse than simply predicting the mean of the target variable. This can occur because the R² is calculated on the hold-out test set, whereas the baseline mean is derived solely from the training set. **(B) Classification Tasks:** Model performance was assessed using Accuracy, AUC, Sensitivity, Specificity, PPV, and NPV. For the Frozen Section Result outcome, macro-averaged one-vs-rest metrics are reported. **Abbreviations:** AUC, Area Under the ROC Curve; FS, Frozen Section; MAE, Mean Absolute Error; NN, Neural Network; NPV, Negative Predictive Value; PPV, Positive Predictive Value; R², Coefficient of Determination; RMSE, Root Mean Squared Error; SMAPE, Symmetric Mean Absolute Percentage Error; SVM, Support Vector Machine.

| **ISUP** | Random forest | Boosting | SVM | **NN** | Mean | Median | **Ridge**  **regression** |
| --- | --- | --- | --- | --- | --- | --- | --- |
| MAE | 0.68 ± 0.10 | 0.67 ± 0.08 | 0.67 ± 0.12 | 0.70 ± 0.06 | 0.92 ± 0.16 | 0.92 ± 0.16 | 0.70 ± 0.05 |
| RMSE | 1.02 ± 0.11 | 0.99 ± 0.07 | 1.02 ± 0.12 | 0.96 ± 0.08 | 1.16 ± 0.13 | 1.16 ± 0.13 | 0.97 ± 0.07 |
| 100-MAPE*100 | 75.3% ± 3.6% | 76.1% ± 3.4% | 77.0% ± 5.5% | 74.4% ± 2.5% | 63.3% ± 5.5% | 63.3% ± 5.5% | 74.6% ± 2.8% |
| R2 | 18.9% ± 14.6% | 23.6% ± 11.1% | 19.7% ± 10.1% | 26.9% ± 17.5% | neg | neg | 26.0% ± 16.2% |
| **Length of Hospital Stay** | Random forest | Boosting | SVM | NN | Mean | Median | Ridge  regression |
| MAE | 0.78 ± 0.15 | 0.68 ± 0.19 | 0.64 ± 0.22 | 0.83 ± 0.18 | 0.62 ± 0.27 | 0.62 ± 0.27 | 0.82 ± 0.17 |
| RMSE | 1.40 ± 0.39 | 1.33 ± 0.50 | 1.32 ± 0.49 | 1.40 ± 0.40 | 1.27 ± 0.58 | 1.27 ± 0.58 | 1.40 ± 0.41 |
| 100-MAPE*100 | 87.3% ± 2.6% | 89.3% ± 2.7% | 90.2% ± 3.4% | 86.6% ± 2.8% | 90.6% ± 4.1% | 90.6% ± 4.1% | 86.8% ± 2.7% |
| R2 | 0.7% ± 1.4% | 0.5% ± 1.0% | neg | neg | neg | neg | neg |
| **Catheter Dwell Time** | **Random forest** | Boosting | SVM | NN | Mean | Median | Ridge  regression |
| MAE | 1.30 ± 0.36 | 0.82 ± 0.47 | 0.79 ± 0.49 | 1.60 ± 0.27 | 1.53 ± 0.40 | 0.79 ± 0.50 | 1.61 ± 0.26 |
| RMSE | 2.92 ± 0.99 | 2.69 ± 1.42 | 2.67 ± 1.45 | 2.96 ± 0.91 | 2.74 ± 1.16 | 2.66 ± 1.45 | 2.96 ± 0.90 |
| 100%-SMAPE | 91.4% ± 1.9% | 95.2% ± 2.4% | 95.5% ± 2.6% | 88.3% ± 1.4% | 88.7% ± 1.8% | 95.5% ± 2.6% | 88.2% ± 1.3% |
| R2 | 3.9% ± 4.8% | neg | neg | 1.1% ± 2.2% | neg | neg | 1.1% ± 2.2% |
| **Hb Drop** | **Random forest** | Boosting | SVM | NN | Mean | Median | Ridge  regression |
| MAE | 0.77 ± 0.09 | 0.75 ± 0.08 | 0.79 ± 0.05 | 0.77 ± 0.05 | 0.76 ± 0.06 | 0.76 ± 0.07 | 0.78 ± 0.05 |
| RMSE | 0.96 ± 0.11 | 0.96 ± 0.10 | 1.03 ± 0.09 | 0.99 ± 0.07 | 0.98 ± 0.08 | 0.98 ± 0.09 | 0.99 ± 0.07 |
| 100%-SMAPE | 80.4% ± 1.1% | 80.9% ± 0.7% | 80.0% ± 1.3% | 80.3% ± 1.2% | 80.9% ± 1.0% | 80.8% ± 1.0% | 80.2% ± 1.3% |
| R2 | 4.3% ± 4.5% | 3.2% ± 3.0% | 1.6% ± 3.1% | 0.7% ± 1.3% | neg | neg | 0.8% ± 1.5% |
| **Prostate Weight** | **Random forest** | Boosting | SVM | NN | Mean | Median | Ridge  regression |
| MAE | 11.76 ± 1.75 | 11.76 ± 1.56 | 12.00 ± 0.72 | 12.10 ± 0.94 | 16.11 ± 1.62 | 15.25 ± 1.22 | 12.01 ± 1.05 |
| RMSE | 16.01 ± 1.76 | 16.43 ± 1.74 | 17.09 ± 1.88 | 16.66 ± 1.11 | 21.45 ± 3.02 | 22.31 ± 2.74 | 16.55 ± 1.25 |
| 100-MAPE*100 | 69.6% ± 13.5% | 70.4% ± 12.9% | 71.0% ± 10.7% | 70.1% ± 11.7% | 56.0% ± 22.4% | 62.7% ± 18.8% | 70.3% ± 11.8% |
| R2 | 43.1% ± 9.2% | 40.0% ± 9.3% | 35.7% ± 20.1% | 36.1% ± 19.8% | neg | neg | 36.8% ± 20.2% |
| **Lymph Node Count** | Random forest | **Boosting** | SVM | NN | Mean | Median | Ridge  regression |
| MAE | 4.27 ± 0.72 | 4.06 ± 0.83 | 4.26 ± 0.77 | 4.37 ± 0.76 | 4.43 ± 0.67 | 4.30 ± 0.71 | 4.33 ± 0.67 |
| RMSE | 5.49 ± 0.92 | 5.22 ± 1.02 | 5.74 ± 1.20 | 5.61 ± 0.95 | 5.60 ± 0.96 | 5.57 ± 0.99 | 5.57 ± 0.89 |
| 100-MAPE*100 | 44.1% ± 3.0% | 47.2% ± 6.6% | 50.1% ± 4.4% | 44.9% ± 3.7% | 40.1% ± 5.8% | 45.6% ± 6.7% | 45.3% ± 3.0% |
| 100%-SMAPE | 79.6% ± 2.1% | 80.2% ± 2.7% | 80.0% ± 2.1% | 79.4% ± 2.2% | 78.8% ± 1.8% | 79.3% ± 1.9% | 79.5% ± 1.8% |
| R2 | 4.7% ± 4.3% | 10.5% ± 6.6% | 4.6% ± 6.1% | 2.0% ± 3.4% | neg | neg | 2.0% ± 3.1% |
| **Blood Loss** | Random forest | Boosting | SVM | **NN** | Mean | Median | **Ridge**  **regression** |
| MAE | 216 ± 31 | 187 ± 41 | 198 ± 40 | 214 ± 37 | 204 ± 38 | 188 ± 46 | 214 ± 36 |
| RMSE | 320 ± 52 | 302 ± 75 | 313 ± 67 | 311 ± 51 | 302 ± 68 | 305 ± 77 | 311 ± 51 |
| 100-MAPE*100 | 36.4% ± 12.6% | 49.2% ± 13.6% | 45.9% ± 13.6% | 37.7% ± 10.9% | 36.2% ± 17.9% | 48.6% ± 15.2% | 37.6% ± 10.9% |
| R2 | 1.2% ± 2.5% | 0.4% ± 0.7% | 0.0% ± 0.0% | 3.2% ± 4.6% | neg | neg | 3.2% ± 4.8% |
| **Intraop. Fluid Admin.** | Random forest | Boosting | SVM | **NN** | Mean | Median | **Ridge**  **regression** |
| MAE | 768 ± 65 | 776 ± 53 | 727 ± 47 | 752 ± 62 | 728 ± 58 | 748 ± 66 | 749 ± 65 |
| RMSE | 1028 ± 74 | 1057 ± 111 | 995 ± 112 | 994 ± 62 | 986 ± 117 | 1022 ± 145 | 989 ± 69 |
| 100-MAPE*100 | 63.0% ± 3.6% | 64.2% ± 3.7% | 66.8% ± 2.5% | 64.2% ± 4.1% | 65.0% ± 3.9% | 65.6% ± 5.0% | 64.4% ± 3.9% |
| R2 | 2.2% ± 4.4% | 0.0% ± 0.0% | 1.8% ± 3.6% | 3.2% ± 5.6% | neg | neg | 3.2% ± 5.0% |
|  |  |  |  |  |  |  |  |
| **Postop.Hct.** | Random  forest | Boosting | **SVM** | NN | Mean | Median | Ridge  regression |
| MAE | 2.24 ± 0.08 | 2.22 ± 0.11 | 2.16 ± 0.10 | 2.15 ± 0.08 | 2.83 ± 0.18 | 2.78 ± 0.21 | 2.15 ± 0.08 |
| RMSE | 2.80 ± 0.17 | 2.84 ± 0.15 | 2.74 ± 0.08 | 2.75 ± 0.13 | 3.54 ± 0.21 | 3.54 ± 0.23 | 2.74 ± 1.13 |
| 100-MAPE*100 | 93.7% ± 0.2% | 93.8% ± 0.4% | 93.9% ± 0.3% | 93.9% ± 0.3% | 92.0% ± 0.7% | 92.1% ± 0.7% | 94.0% ± 0.3% |
| R2 | 35.1% ± 12.8% | 34.3% ± 8.3% | 38.3% ± 10.2% | 37.9% ± 10.9% | neg | neg | 38.0% ± 10.9% |
| **Postop.Hb.** | Random  forest | Boosting | **SVM** | **NN** | Mean | Median | **Ridge**  **regression** |
| MAE | 0.79 ± 0.03 | 0.79 ± 0.05 | 0.74 ± 0.03 | 0.76 ± 0.03 | 1.01 ± 0.10 | 1.01 ± 0.10 | 0.76 ± 0.03 |
| RMSE | 1.00 ± 0.07 | 0.98 ± 0.08 | 0.96 ± 0.07 | 0.98 ± 0.07 | 1.26 ± 0.10 | 1.28 ± 0.12 | 0.98 ± 0.07 |
| 100-MAPE*100 | 93.6% ± 0.4% | 93.6% ± 0.6% | 93.9% ± 0.3% | 93.8% ± 0.3% | 91.7% ± 0.9% | 91.6% ± 1.0% | 93.8% ± 0.3% |
| R2 | 35.0% ± 11.3% | 36.6% ± 13.1% | 39.2% ± 12.3% | 37.2% ± 12.3% | neg | neg | 36.7% ± 12.2% |

**(A) Regression Tasks**

| **Frozen Section Result** | Random forest | Boosting | **SVM** | NN | Mode | **Logistic**  **regression** |
| --- | --- | --- | --- | --- | --- | --- |
| Accuracy | 86.5% ± 2.8% | 83.0% ± 5.0% | 86.1% ± 2.9% | 86.6% ± 3.1% | 41.7% ± 3.6% | 84.7% ± 3.6% |
| AUC | 82.6% ± 3.1% | 80.1% ± 5.6% | 74.9% ± 6.2% | 85.1% ± 3.3% | N/A | 85.4% ± 5.0% |
| Sensitivity | 61.1% ± 1.2% | 58.9% ± 1.9% | 60.9% ± 0.7% | 61.2% ± 0.9% | 33.3% ± 0.0% | 60.0% ± 1.6% |
| Specificity | 91.4% ± 1.5% | 89.7% ± 2.8% | 91.1% ± 1.9% | 91.4% ± 1.8% | 66.7% ± 0.0% | 90.8% ± 1.8% |
| PPV | 58.9% ± 0.9% | 56.9% ± 3.5% | 58.2% ± 1.8% | 58.7% ± 1.4% | 13.9% ± 1.2% | 58.3% ± 1.1% |
| NPV | 93.3% ± 1.1% | 90.9% ± 2.8% | 92.9% ± 1.5% | 93.1% ± 1.4% | 47.2% ± 1.2% | 91.8% ± 1.9% |
| **FS Decision (yes/no)** | **Random forest** | Boosting | SVM | NN | Mode | **Logistic**  **regression** |
| Accuracy | 92.8% ± 1.8% | 90.6% ± 3.3% | 91.9% ± 1.2% | 93.3% ± 1.5% | 53.4% ± 7.4% | 92.4% ± 1.9% |
| AUC | 92.2% ± 4.0% | 93.4% ± 3.2% | 93.5% ± 3.6% | 93.3% ± 3.3% | N/A | 93.4% ± 3.0% |
| Sensitivity | 88.3% ± 5.6% | 88.3% ± 5.6% | 88.3% ± 5.6% | 89.2% ± 4.8% | 0.0% ± 0.0% | 89.2% ± 4.8% |
| Specificity | 96.4% ± 3.9% | 93.0% ± 7.8% | 95.0% ± 4.7% | 96.4% ± 3.9% | 100.0% ± 0.0% | 94.9% ± 4.3% |
| PPV | 96.1% ± 3.2% | 91.6% ± 10.5% | 94.0% ± 6.1% | 96.1% ± 3.2% | 0.0% ± 0.0% | 93.9% ± 5.2% |
| NPV | 90.7% ± 4.3% | 90.5% ± 4.1% | 90.6% ± 4.2% | 91.4% ± 3.5% | 53.4% ± 7.4% | 91.2% ± 3.5% |

**(B) Classification Tasks**

**Supplementary Table S5: Performance metrics — RARP subgroup, with nerve-sparing parameters as input features.** The table summarizes the predictive performance of four machine learning models (Random Forest, Gradient Boosting, Support Vector Machine [SVM], and Neural Network [NN]) compared to baseline models (Mean/Median for regression, Mode for classification, and multivariable Ridge regression). Performance was evaluated using a 5-fold cross-validation scheme. **(A) Regression Tasks:** Model performance was assessed using MAE, RMSE, SMAPE-based Accuracy (100% − SMAPE) for outcomes with zero values where MAPE is undefined, and R². Lower MAE and RMSE values indicate better performance; higher SMAPE-based Accuracy and R² values indicate better model fit. An R² value of 'neg.' indicates performance worse than the mean predictor. **(B) Classification Tasks:** Model performance was assessed using Accuracy, AUC, Sensitivity, Specificity, PPV, and NPV. For the Frozen Section Result outcome, macro-averaged one-vs-rest metrics are reported. **Abbreviations:** AUC, Area Under the ROC Curve; FS, Frozen Section; MAE, Mean Absolute Error; NN, Neural Network; NPV, Negative Predictive Value; PPV, Positive Predictive Value; R², Coefficient of Determination; RMSE, Root Mean Squared Error; SMAPE, Symmetric Mean Absolute Percentage Error; SVM, Support Vector Machine.

| **ISUP** | Random forest | Boosting | SVM | **NN** | Mean | Median | **Ridge**  **regression** |
| --- | --- | --- | --- | --- | --- | --- | --- |
| MAE | 0.73 ± 0.10 | 0.68 ± 0.11 | 0.71 ± 0.12 | 0.70 ± 0.12 | 0.92 ± 0.16 | 0.92 ± 0.16 | 0.70 ± 0.13 |
| RMSE | 1.03 ± 0.11 | 1.01 ± 0.10 | 1.01 ± 0.11 | 0.98 ± 0.13 | 1.16 ± 0.13 | 1.16 ± 0.13 | 0.99 ± 0.13 |
| 100-MAPE*100 | 73.6% ± 3.5% | 76.1% ± 4.0% | 74.7% ± 4.6% | 73.9% ± 5.0% | 63.3% ± 5.5% | 63.3% ± 5.5% | 73.7% ± 4.9% |
| R2 | 16.9% ± 11.5% | 21.4% ± 11.5% | 19.9% ± 13.4% | 24.9% ± 11.5% | neg | neg | 24.3% ± 11.1% |
| **Length of Hospital Stay** | Random forest | Boosting | SVM | NN | Mean | Median | Ridge  regression |
| MAE | 0.82 ± 0.19 | 0.65 ± 0.20 | 0.64 ± 0.22 | 0.83 ± 0.22 | 0.62 ± 0.27 | 0.62 ± 0.27 | 0.83 ± 0.22 |
| RMSE | 1.42 ± 0.42 | 1.28 ± 0.52 | 1.32 ± 0.49 | 1.39 ± 0.45 | 1.27 ± 0.58 | 1.27 ± 0.58 | 1.38 ± 0.44 |
| 100-MAPE*100 | 86.7% ± 3.3% | 89.7% ± 3.1% | 90.2% ± 3.4% | 86.6% ± 3.4% | 90.6% ± 4.1% | 90.6% ± 4.1% | 86.8% ± 3.4% |
| R2 | neg | neg | neg | neg | neg | neg | neg |
| **Catheter Dwell Time** | **Random forest** | Boosting | SVM | **NN** | Mean | Median | **Ridge**  **regression** |
| MAE | 1.38 ± 0.35 | 0.84 ± 0.46 | 0.88 ± 0.48 | 1.54 ± 0.32 | 1.53 ± 0.40 | 0.79 ± 0.50 | 1.54 ± 0.32 |
| RMSE | 2.99 ± 1.01 | 2.71 ± 1.39 | 2.74 ± 1.42 | 2.95 ± 0.91 | 2.74 ± 1.16 | 2.66 ± 1.45 | 2.95 ± 0.91 |
| 100%-SMAPE | 90.9% ± 1.8% | 95.0% ± 2.3% | 94.9% ± 2.7% | 88.9% ± 1.9% | 88.7% ± 1.8% | 95.5% ± 2.6% | 88.9% ± 1.9% |
| R2 | 1.3% ± 2.6% | neg | neg | 1.3% ± 1.9% | neg | neg | 1.3% ± 1.9% |
| **Hb Drop** | Random forest | **Boosting** | SVM | NN | Mean | Median | Ridge  regression |
| MAE | 0.79 ± 0.10 | 0.76 ± 0.08 | 0.80 ± 0.09 | 0.78 ± 0.07 | 0.76 ± 0.06 | 0.76 ± 0.07 | 0.79 ± 0.07 |
| RMSE | 0.97 ± 0.12 | 0.96 ± 0.10 | 1.06 ± 0.14 | 0.99 ± 0.10 | 0.98 ± 0.08 | 0.98 ± 0.09 | 1.00 ± 0.10 |
| 100%-SMAPE | 80.0% ± 1.0% | 80.6% ± 1.1% | 79.8% ± 2.1% | 80.1% ± 1.1% | 80.9% ± 1.0% | 80.8% ± 1.0% | 79.9% ± 1.2% |
| R2 | 2.8% ± 3.8% | 4.0% ± 3.4% | 2.4% ± 3.4% | 0.9% ± 1.7% | neg | neg | 0.4% ± 0.8% |
| **Prostate Weight** | **Random forest** | Boosting | SVM | NN | Mean | Median | Ridge  regression |
| MAE | 12.12 ± 1.82 | 12.58 ± 1.52 | 12.14 ± 0.50 | 12.40 ± 0.84 | 16.11 ± 1.62 | 15.25 ± 1.22 | 12.31 ± 0.92 |
| RMSE | 16.43 ± 1.67 | 16.66 ± 1.33 | 17.31 ± 1.61 | 17.18 ± 1.17 | 21.45 ± 3.02 | 22.31 ± 2.74 | 17.06 ± 1.27 |
| 100-MAPE*100 | 67.5% ± 15.5% | 66.5% ± 15.5% | 70.0% ± 11.9% | 69.1% ± 10.8% | 56.0% ± 22.4% | 62.7% ± 18.8% | 69.3% ± 11.0% |
| R2 | 40.0% ± 9.0% | 36.8% ± 17.3% | 33.8% ± 19.8% | 34.2% ± 18.4% | neg | neg | 35.0% ± 18.7% |
| **Lymph Node Count** | Random forest | **Boosting** | SVM | NN | Mean | Median | Ridge  regression |
| MAE | 4.21 ± 0.75 | 4.03 ± 0.81 | 4.00 ± 0.78 | 4.32 ± 0.86 | 4.43 ± 0.67 | 4.30 ± 0.71 | 4.28 ± 0.80 |
| RMSE | 5.45 ± 0.95 | 5.19 ± 1.00 | 5.33 ± 1.03 | 5.50 ± 1.15 | 5.60 ± 0.96 | 5.57 ± 0.99 | 5.49 ± 1.11 |
| 100-MAPE*100 | 45.1% ± 3.3% | 47.5% ± 6.1% | 52.0% ± 3.1% | 45.5% ± 4.2% | 40.1% ± 5.8% | 45.6% ± 6.7% | 45.6% ± 3.6% |
| 100%-SMAPE | 79.9% ± 2.2% | 80.3% ± 2.5% | 80.8% ± 2.3% | 79.6% ± 2.5% | 78.8% ± 1.8% | 79.3% ± 1.9% | 79.7% ± 2.3% |
| R2 | 6.7% ± 7.6% | 11.2% ± 7.6% | 7.8% ± 9.9% | 6.0% ± 9.1% | neg | neg | 5.7% ± 8.5% |
| **Blood Loss** | Random forest | Boosting | SVM | **NN** | Mean | Median | **Ridge**  **regression** |
| MAE | 218 ± 32 | 188 ± 41 | 197 ± 43 | 210 ± 38 | 204 ± 38 | 188 ± 46 | 210 ± 38 |
| RMSE | 322 ± 53 | 303 ± 74 | 308 ± 71 | 308 ± 51 | 302 ± 68 | 305 ± 77 | 308 ± 51 |
| 100-MAPE*100 | 36.7% ± 12.2% | 48.7% ± 13.7% | 48.3% ± 13.0% | 38.6% ± 12.2% | 36.2% ± 17.9% | 48.6% ± 15.2% | 38.6% ± 12.1% |
| R2 | 1.0% ± 2.1% | 0.3% ± 0.5% | 0.0% ± 0.0% | 3.6% ± 5.7% | neg | neg | 3.6% ± 5.8% |
| **Intraop. Fluid Admin.** | Random forest | Boosting | SVM | **NN** | Mean | Median | **Ridge**  **regression** |
| MAE | 784 ± 55 | 745 ± 58 | 716 ± 61 | 732 ± 66 | 728 ± 58 | 748 ± 66 | 730 ± 66 |
| RMSE | 1045 ± 84 | 1026 ± 114 | 994 ± 130 | 982 ± 88 | 986 ± 117 | 1022 ± 145 | 979 ± 89 |
| 100-MAPE*100 | 62.1% ± 3.5% | 65.6% ± 2.6% | 67.5% ± 3.3% | 65.1% ± 3.6% | 65.0% ± 3.9% | 65.6% ± 5.0% | 65.1% ± 3.6% |
| R2 | 0.3% ± 0.6% | 1.0% ± 2.0% | 1.3% ± 2.6% | 2.4% ± 3.2% | neg | neg | 2.5% ± 3.2% |
|  |  |  |  |  |  |  |  |
| **Postop.Hct.** | Random  forest | Boosting | **SVM** | NN | Mean | Median | Ridge  regression |
| MAE | 2.30 ± 0.06 | 2.38 ± 0.12 | 2.16 ± 0.14 | 2.21 ± 0.07 | 2.83 ± 0.18 | 2.78 ± 0.21 | 2.21 ± 0.07 |
| RMSE | 2.88 ± 0.11 | 2.97 ± 0.09 | 2.76 ± 0.07 | 2.77 ± 0.11 | 3.54 ± 0.21 | 3.54 ± 0.23 | 2.77 ± 0.11 |
| 100-MAPE*100 | 93.5% ± 0.2% | 93.3% ± 0.4% | 93.9% ± 0.4% | 93.8% ± 0.2% | 92.0% ± 0.7% | 92.1% ± 0.7% | 93.8% ± 0.2% |
| R2 | 31.9% ± 9.8% | 28.0% ± 6.6% | 37.7% ± 9.9% | 36.9% ± 11.6% | neg | neg | 36.9% ± 11.6% |
| **Postop.Hb.** | Random  forest | Boosting | **SVM** | **NN** | Mean | Median | **Ridge**  **regression** |
| MAE | 0.81 ± 0.03 | 0.81 ± 0.04 | 0.77 ± 0.05 | 0.77 ± 0.03 | 1.01 ± 0.10 | 1.01 ± 0.10 | 0.78 ± 0.03 |
| RMSE | 1.02 ± 0.07 | 1.02 ± 0.08 | 0.99 ± 0.08 | 0.99 ± 0.08 | 1.26 ± 0.10 | 1.28 ± 1.12 | 1.00 ± 0.08 |
| 100-MAPE*100 | 93.4% ± 0.4% | 93.4% ± 0.5% | 93.7% ± 0.5% | 93.7% ± 0.3% | 91.7% ± 0.9% | 91.6% ± 1.0% | 93.7% ± 0.3% |
| R2 | 32.6% ± 12.1% | 31.6% ± 12.6% | 35.4% ± 13.7% | 35.2% ± 14.4% | neg | neg | 34.8% ± 14.6% |

**(A) Regression Tasks**

| **Frozen Section Result** | Random forest | Boosting | **SVM** | NN | Mode | **Logistic**  **regression** |
| --- | --- | --- | --- | --- | --- | --- |
| Accuracy | 64.6% ± 4.4% | 58.7% ± 5.6% | 64.5% ± 5.5% | 63.6% ± 6.7% | 41.7% ± 3.6% | 64.1% ± 6.3% |
| AUC | 63.9% ± 4.8% | 60.8% ± 4.1% | 67.5% ± 7.6% | 65.6% ± 2.9% | N/A | 70.5% ± 6.4% |
| Sensitivity | 46.2% ± 3.9% | 42.1% ± 4.3% | 46.2% ± 5.0% | 45.1% ± 5.8% | 33.3% ± 0.0% | 45.5% ± 5.2% |
| Specificity | 78.0% ± 3.2% | 75.0% ± 4.2% | 78.0% ± 3.7% | 76.9% ± 4.5% | 66.7% ± 0.0% | 77.9% ± 4.2% |
| PPV | 43.5% ± 3.3% | 40.8% ± 4.7% | 43.7% ± 3.4% | 42.5% ± 4.3% | 13.9% ± 1.2% | 43.9% ± 4.3% |
| NPV | 78.7% ± 3.5% | 75.1% ± 4.2% | 78.7% ± 4.1% | 77.5% ± 5.2% | 47.2% ± 1.2% | 78.3% ± 4.6% |
| **FS Decision (yes/no)** | **Random forest** | Boosting | SVM | NN | Mode | **Logistic**  **regression** |
| Accuracy | 67.7% ± 6.6% | 64.1% ± 7.4% | 66.4% ± 5.0% | 68.2% ± 3.9% | 53.4% ± 7.4% | 69.1% ± 4.6% |
| AUC | 74.9% ± 1.7% | 70.6% ± 8.4% | 73.7% ± 4.2% | 73.1% ± 4.3% | N/A | 74.4% ± 5.4% |
| Sensitivity | 60.0% ± 13.9% | 57.6% ± 14.5% | 54.5% ± 8.7% | 60.1% ± 9.2% | 0.0% ± 0.0% | 60.8% ± 5.9% |
| Specificity | 77.0% ± 11.2% | 72.2% ± 7.6% | 77.4% ± 8.4% | 76.2% ± 5.4% | 100.0% ± 0.0% | 77.1% ± 6.3% |
| PPV | 69.7% ± 12.3% | 63.9% ± 8.9% | 67.7% ± 13.5% | 68.3% ± 9.3% | 0.0% ± 0.0% | 69.5% ± 11.1% |
| NPV | 69.1% ± 10.3% | 66.4% ± 12.2% | 66.2% ± 6.1% | 68.7% ± 8.5% | 53.4% ± 7.4% | 69.1% ± 7.3% |

**(B) Classification Tasks**

**Supplementary Table S6:** **Performance metrics — RARP subgroup, without nerve-sparing parameters as input features** The table summarizes the predictive performance of four machine learning models (Random Forest, Gradient Boosting, Support Vector Machine [SVM], and Neural Network [NN]) compared to baseline models (Mean/Median for regression, Mode for classification, and multivariable Ridge regression). Performance was evaluated using a 5-fold cross-validation scheme. **(A) Regression Tasks:** Model performance was assessed using MAE, RMSE, SMAPE-based Accuracy (100% − SMAPE) for outcomes with zero values where MAPE is undefined, and R². Lower MAE and RMSE values indicate better performance; higher SMAPE-based Accuracy and R² values indicate better model fit. An R² value of 'neg.' indicates performance worse than the mean predictor. **(B) Classification Tasks:** Model performance was assessed using Accuracy, AUC, Sensitivity, Specificity, PPV, and NPV. For the Frozen Section Result outcome, macro-averaged one-vs-rest metrics are reported. **Abbreviations:** AUC, Area Under the ROC Curve; FS, Frozen Section; MAE, Mean Absolute Error; NN, Neural Network; NPV, Negative Predictive Value; PPV, Positive Predictive Value; R², Coefficient of Determination; RMSE, Root Mean Squared Error; SMAPE, Symmetric Mean Absolute Percentage Error; SVM, Support Vector Machine.

| **ISUP** | **Random forest** | Boosting | SVM | NN | Mean | Median | Ridge  regression |
| --- | --- | --- | --- | --- | --- | --- | --- |
| MAE | 0.70 ± 0.15 | 0.75 ± 0.18 | 0.78 ± 0.12 | 0.80 ± 0.13 | 1.08 ± 0.12 | 1.08 ± 0.12 | 0.79 ± 0.11 |
| RMSE | 0.97 ± 0.13 | 1.02 ± 0.16 | 1.05 ± 0.11 | 1.06 ± 0.12 | 1.26 ± 0.11 | 1.26 ± 0.11 | 1.05 ± 0.10 |
| 100-MAPE*100 | 74.8% ± 5.2% | 73.4% ± 6.1% | 70.7% ± 5.0% | 69.3% ± 3.2% | 58.7% ± 4.6% | 58.7% ± 4.6% | 70.0% ± 2.5% |
| R2 | 36.9% ± 19.1% | 30.6% ± 20.1% | 25.8% ± 19.4% | 25.1% ± 18.7% | neg | neg | 27.2% ± 15.8% |
| **Length of Hospital Stay** | Random forest | Boosting | SVM | NN | Mean | Median | Ridge  regression |
| MAE | 1.93 ± 0.51 | 1.75 ± 0.66 | 2.09 ± 0.88 | 2.51 ± 0.37 | 1.79 ± 0.52 | 1.52 ± 0.72 | 2.51 ± 0.37 |
| RMSE | 3.07 ± 1.23 | 2.94 ± 1.47 | 4.54 ± 3.26 | 3.86 ± 0.98 | 2.85 ± 1.42 | 2.60 ± 1.63 | 3.86 ± 0.98 |
| 100-MAPE*100 | 82.5% ± 4.2% | 85.3% ± 4.0% | 81.7% ± 8.0% | 77.3% ± 3.6% | 84.1% ± 2.8% | 87.2% ± 3.7% | 77.3% ± 3.6% |
| R2 | neg | neg | neg | neg | neg | neg | neg |
| **Catheter Dwell Time** | Random forest | Boosting | SVM | NN | Mean | Median | Ridge  regression |
| MAE | 4.46 ± 1.14 | 3.87 ± 1.60 | 7.32 ± 6.78 | 5.47 ± 0.66 | 4.68 ± 1.16 | 3.06 ± 1.68 | 5.42 ± 0.72 |
| RMSE | 7.60 ± 2.71 | 7.58 ± 3.74 | 21.35 ± 28.51 | 8.41 ± 2.20 | 6.92 ± 3.20 | 7.05 ± 3.79 | 8.34 ± 2.30 |
| 100-MAPE*100 | 61.6% ± 9.0% | 72.9% ± 10.8% | 26.9% ± 86.6% | 48.6% ± 9.6% | 61.1% ± 3.7% | 85.8% ± 3.8% | 49.2% ± 9.2% |
| 100%-SMAPE | 83.8% ± 2.2% | 87.2% ± 3.4% | 85.0% ± 1.9% | 76.4% ± 3.6% | 81.5% ± 0.6% | 90.4% ± 3.2% | 76.4% ± 3.8% |
| R2 | neg | neg | 1.4% ± 2.8% | 0.5% ± 0.9% | neg | neg | 0.4% ± 0.8% |
| **Hb Drop** | Random forest | **Boosting** | SVM | NN | Mean | Median | Ridge  regression |
| MAE | 1.04 ± 0.11 | 1.01 ± 0.13 | 1.10 ± 0.10 | 1.13 ± 0.12 | 1.08 ± 0.10 | 1.08 ± 0.10 | 1.14 ± 0.13 |
| RMSE | 1.30 ± 0.12 | 1.24 ± 0.18 | 1.38 ± 0.15 | 1.37 ± 0.15 | 1.32 ± 0.13 | 1.32 ± 0.13 | 1.38 ± 0.16 |
| 100-MAPE*100 | 70.8% ± 6.0% | 71.8% ± 4.7% | 69.0% ± 7.1% | 69.7% ± 2.5% | 69.2% ± 6.7% | 68.9% ± 6.9% | 69.6% ± 2.5% |
| 100%-SMAPE | 87.5% ± 1.5% | 87.8% ± 1.4% | 86.9% ± 1.4% | 86.5% ± 1.4% | 87.0% ± 1.4% | 87.0% ± 1.4% | 86.4% ± 1.5% |
| R2 | 4.9% ± 5.4% | 13.4% ± 10.6% | 1.1% ± 2.1% | 5.8% ± 11.6% | neg | neg | 5.8% ± 11.7% |
| **Prostate Weight** | Random forest | Boosting | **SVM** | NN | Mean | Median | Ridge  regression |
| MAE | 19.10 ± 5.19 | 21.85 ± 5.62 | 18.24 ± 3.92 | 19.20 ± 1.79 | 18.79 ± 7.05 | 18.02 ± 6.85 | 18.92 ± 1.88 |
| RMSE | 26.90 ± 11.23 | 29.91 ± 12.07 | 25.20 ± 9.55 | 25.54 ± 4.69 | 26.77 ± 13.70 | 27.04 ± 13.84 | 25.27 ± 4.92 |
| 100-MAPE*100 | 65.0% ± 9.1% | 57.9% ± 10.1% | 66.7% ± 5.5% | 63.9% ± 4.2% | 62.3% ± 12.5% | 67.0% ± 11.4% | 64.4% ± 4.3% |
| R2 | 9.7% ± 8.4% | 1.7% ± 2.3% | 12.4% ± 13.2% | 11.8% ± 23.7% | neg | neg | 11.7% ± 23.4% |
| **Lymph Node Count** | Random forest | Boosting | SVM | NN | Mean | Median | Ridge  regression |
| MAE | 8.06 ± 0.98 | 7.80 ± 1.04 | 7.98 ± 1.06 | 9.01 ± 1.17 | 7.84 ± 1.03 | 7.71 ± 1.15 | 8.89 ± 1.03 |
| RMSE | 10.89 ± 1.40 | 10.36 ± 1.55 | 10.50 ± 1.53 | 11.69 ± 1.19 | 10.36 ± 1.47 | 10.34 ± 1.53 | 11.59 ± 1.14 |
| 100%-SMAPE | 80.8% ± 3.7% | 80.9% ± 2.5% | 80.6% ± 2.2% | 78.4% ± 3.1% | 80.9% ± 2.7% | 81.1% ± 2.5% | 78.8% ± 2.9% |
| R2 | neg | neg | neg | neg | neg | neg | neg |
| **Blood Loss** | Random forest | Boosting | SVM | NN | Mean | Median | Ridge  regression |
| MAE | 610 ± 124 | 526 ± 164 | 500 ± 150 | 778 ± 187 | 510 ± 129 | 488 ± 141 | 750 ± 179 |
| RMSE | 819 ± 199 | 732 ± 271 | 700 ± 270 | 979 ± 265 | 703 ± 235 | 688 ± 259 | 954 ± 252 |
| 100-MAPE*100 | 26.7% ± 23.6% | 43.9% ± 17.0% | 45.8% ± 15.3% | 20.9% ± 25.8% | 40.2% ± 17.1% | 46.5% ± 15.8% | 23.2% ± 23.8% |
| R2 | neg | neg | neg | neg | neg | neg | neg |
| **Intraop. Fluid Admin.** | Random forest | Boosting | SVM | NN | Mean | Median | Ridge  regression |
| MAE | 1279 ± 416 | 1163 ± 503 | 1152 ± 479 | 1614 ± 317 | 1157 ± 492 | 1094 ± 523 | 1567 ± 334 |
| RMSE | 1601 ± 605 | 1471 ± 677 | 1473 ± 648 | 2022 ± 572 | 1461 ± 661 | 1413 ± 689 | 1963 ± 590 |
| 100-MAPE*100 | 60.2% ± 6.4% | 63.5% ± 9.6% | 64.1% ± 7.9% | 51.8% ± 7.9% | 62.5% ± 10.6% | 66.0% ± 9.9% | 53.1% ± 7.5% |
| R2 | neg | 2.1% ± 4.2% | neg | neg | neg | neg | neg |
|  |  |  |  |  |  |  |  |
| **Postop.Hct.** | Random  forest | Boosting | SVM | **NN** | Mean | Median | **Ridge**  **regression** |
| MAE | 3.13 ± 0.18 | 3.21 ± 0.47 | 3.15 ± 0.31 | 3.10 ± 0.39 | 3.47 ± 0.49 | 3.51 ± 0.55 | 3.10 ± 0.39 |
| RMSE | 3.86 ± 0.33 | 3.95 ± 0.55 | 3.95 ± 0.46 | 3.85 ± 0.54 | 4.10 ± 0.49 | 4.16 ± 0.57 | 3.85 ± 0.54 |
| 100-MAPE*100 | 89.5% ± 0.7% | 89.2% ± 1.3% | 89.4% ± 1.2% | 89.5% ± 1.8% | 88.3% ± 1.3% | 88.4% ± 1.5% | 89.6% ± 1.8% |
| R2 | 12.0% ± 14.6% | 7.7% ± 9.2% | 16.4% ± 14.4% | 18.4% ± 17.1% | neg | neg | 18.4% ± 17.1% |
| **Postop.Hb.** | **Random**  **forest** | Boosting | SVM | NN | Mean | Median | Ridge  regression |
| MAE | 1.06 ± 0.13 | 1.13 ± 0.12 | 1.08 ± 0.19 | 1.14 ± 0.13 | 1.27 ± 0.11 | 1.28 ± 0.13 | 1.15 ± 0.13 |
| RMSE | 1.30 ± 0.20 | 1.36 ± 0.18 | 1.34 ± 0.27 | 1.38 ± 0.16 | 1.47 ± 0.17 | 1.50 ± 0.20 | 1.39 ± 0.17 |
| 100-MAPE*100 | 89.7% ± 1.3% | 89.1% ± 0.9% | 89.5% ± 1.8% | 88.9% ± 1.2% | 87.7% ± 0.8% | 87.8% ± 0.9% | 88.8% ± 1.2% |
| R2 | 13.8% ± 14.1% | 9.0% ± 7.5% | 11.5% ± 14.0% | 10.4% ± 14.3% | neg | neg | 10.1% ± 13.9% |

**(A) Regression Tasks**

| **Frozen Section Result** | Random forest | Boosting | SVM | **NN** | Mode | **Logistic**  **regression** |
| --- | --- | --- | --- | --- | --- | --- |
| Accuracy | 54.4% ± 8.7% | 49.0% ± 10.0% | 56.3% ± 10.5% | 57.5% ± 6.4% | 59.6% ± 10.2% | 54.2% ± 12.4% |
| AUC | 52.0% ± 10.2% | 47.2% ± 5.5% | 43.9% ± 13.4% | 58.9% ± 7.1% | N/A | 57.9% ± 11.1% |
| Sensitivity | 36.1% ± 6.0% | 31.7% ± 7.4% | 35.3% ± 5.3% | 36.2% ± 4.5% | 36.7% ± 6.7% | 35.2% ± 9.6% |
| Specificity | 63.0% ± 12.5% | 67.0% ± 5.9% | 61.9% ± 8.1% | 69.9% ± 3.9% | 63.3% ± 6.7% | 69.0% ± 7.8% |
| PPV | 30.0% ± 9.7% | 28.8% ± 7.5% | 23.2% ± 5.9% | 34.6% ± 2.5% | 22.0% ± 5.9% | 34.5% ± 9.1% |
| NPV | 66.7% ± 16.7% | 67.3% ± 6.7% | 50.0% ± 10.9% | 70.9% ± 5.9% | 48.6% ± 9.2% | 70.6% ± 11.1% |
| **FS Decision (yes/no)** | **Random forest** | Boosting | SVM | **NN** | Mode | **Logistic**  **regression** |
| Accuracy | 56.4% ± 15.3% | 55.3% ± 12.7% | 59.6% ± 10.2% | 60.6% ± 15.1% | 59.6% ± 10.2% | 55.3% ± 12.2% |
| AUC | 53.2% ± 11.0% | 51.8% ± 13.1% | 49.7% ± 9.2% | 56.0% ± 7.2% | N/A | 55.2% ± 10.0% |
| Sensitivity | 70.6% ± 15.4% | 67.7% ± 15.8% | 74.4% ± 18.9% | 76.7% ± 16.5% | 100.0% ± 0.0% | 68.8% ± 16.6% |
| Specificity | 35.8% ± 18.3% | 38.1% ± 14.4% | 31.6% ± 21.5% | 36.2% ± 15.1% | 0.0% ± 0.0% | 33.8% ± 12.0% |
| PPV | 62.0% ± 15.2% | 61.6% ± 9.8% | 61.8% ± 13.3% | 63.5% ± 13.2% | 59.6% ± 10.2% | 59.9% ± 14.2% |
| NPV | 44.3% ± 19.6% | 47.2% ± 25.0% | 40.3% ± 20.6% | 53.7% ± 26.2% | 0.0% ± 0.0% | 44.3% ± 19.6% |

**(B) Classification Tasks**

**Supplementary Table S7:** **Performance metrics — ORP subgroup, with nerve-sparing parameters as input features** The table summarizes the predictive performance of four machine learning models (Random Forest, Gradient Boosting, Support Vector Machine [SVM], and Neural Network [NN]) compared to baseline models (Mean/Median for regression, Mode for classification, and multivariable Ridge regression). Performance was evaluated using a 5-fold cross-validation scheme. **(A) Regression Tasks:** Model performance was assessed using MAE, RMSE, SMAPE-based Accuracy (100% − SMAPE) for outcomes with zero values where MAPE is undefined, and R². Lower MAE and RMSE values indicate better performance; higher SMAPE-based Accuracy and R² values indicate better model fit. An R² value of 'neg.' indicates performance worse than the mean predictor. **(B) Classification Tasks:** Model performance was assessed using Accuracy, AUC, Sensitivity, Specificity, PPV, and NPV. For the Frozen Section Result outcome, macro-averaged one-vs-rest metrics are reported. **Abbreviations:** AUC, Area Under the ROC Curve; FS, Frozen Section; MAE, Mean Absolute Error; NN, Neural Network; NPV, Negative Predictive Value; PPV, Positive Predictive Value; R², Coefficient of Determination; RMSE, Root Mean Squared Error; SMAPE, Symmetric Mean Absolute Percentage Error; SVM, Support Vector Machine.

| **ISUP** | **Random forest** | Boosting | **SVM** | NN | Mean | Median | Ridge  regression |
| --- | --- | --- | --- | --- | --- | --- | --- |
| MAE | 0.71 ± 0.15 | 0.77 ± 0.21 | 0.74 ± 0.15 | 0.81 ± 0.13 | 1.08 ± 0.12 | 1.08 ± 0.12 | 0.79 ± 0.11 |
| RMSE | 0.98 ± 0.14 | 1.05 ± 0.19 | 0.98 ± 0.12 | 1.06 ± 0.11 | 1.26 ± 0.11 | 1.26 ± 0.11 | 1.04 ± 0.11 |
| 100-MAPE*100 | 74.7% ± 5.0% | 72.9% ± 7.4% | 73.4% ± 6.4% | 69.7% ± 4.2% | 58.7% ± 4.6% | 58.7% ± 4.6% | 70.7% ± 3.5% |
| R2 | 34.9% ± 19.8% | 31.2% ± 19.7% | 35.1% ± 20.0% | 26.1% ± 16.2% | neg | neg | 28.7% ± 14.9% |
| **Length of Hospital Stay** | Random forest | Boosting | SVM | NN | Mean | Median | Ridge  regression |
| MAE | 1.86 ± 0.55 | 1.64 ± 0.72 | 2.19 ± 1.02 | 2.31 ± 0.31 | 1.79 ± 0.52 | 1.52 ± 0.72 | 2.30 ± 0.32 |
| RMSE | 2.95 ± 1.36 | 2.78 ± 1.61 | 4.99 ± 4.08 | 3.56 ± 0.95 | 2.85 ± 1.42 | 2.60 ± 1.63 | 3.54 ± 0.97 |
| 100-MAPE*100 | 83.2% ± 4.4% | 86.2% ± 3.5% | 80.7% ± 9.8% | 79.0% ± 1.5% | 84.1% ± 2.8% | 87.2% ± 3.7% | 79.1% ± 1.5% |
| R2 | neg | neg | neg | 0.4% ± 0.8% | neg | neg | neg |
| **Catheter Dwell Time** | **Random forest** | Boosting | SVM | NN | Mean | Median | Ridge  regression |
| MAE | 4.37 ± 0.86 | 4.41 ± 1.65 | 5.19 ± 1.60 | 5.17 ± 1.05 | 4.68 ± 1.16 | 3.06 ± 1.68 | 5.10 ± 1.03 |
| RMSE | 7.48 ± 2.43 | 8.01 ± 3.59 | 10.40 ± 6.42 | 8.31 ± 2.88 | 6.92 ± 3.20 | 7.05 ± 3.79 | 8.22 ± 2.87 |
| 100-MAPE*100 | 61.8% ± 10.3% | 66.8% ± 14.2% | 55.6% ± 19.7% | 52.7% ± 12.1% | 61.1% ± 3.7% | 85.8% ± 3.8% | 53.6% ± 11.4% |
| 100%-SMAPE | 84.0% ± 2.3% | 85.2% ± 3.6% | 81.6% ± 3.0% | 79.4% ± 3.2% | 81.5% ± 0.6% | 90.4% ± 3.2% | 79.8% ± 2.9% |
| R2 | 1.1% ± 2.2% | neg | neg | neg | neg | neg | neg |
| **Hb Drop** | Random forest | **Boosting** | SVM | NN | Mean | Median | Ridge  regression |
| MAE | 1.03 ± 0.10 | 0.99 ± 0.09 | 1.09 ± 0.11 | 1.10 ± 0.08 | 1.08 ± 0.10 | 1.08 ± 0.10 | 1.11 ± 0.09 |
| RMSE | 1.31 ± 0.12 | 1.24 ± 0.17 | 1.38 ± 0.17 | 1.35 ± 0.14 | 1.32 ± 0.13 | 1.32 ± 0.13 | 1.36 ± 0.15 |
| 100-MAPE*100 | 71.2% ± 6.0% | 72.9% ± 4.8% | 69.4% ± 7.3% | 70.3% ± 1.6% | 69.2% ± 6.7% | 68.9% ± 6.9% | 70.2% ± 1.5% |
| 100%-SMAPE | 87.7% ± 1.4% | 88.1% ± 1.1% | 87.1% ± 1.5% | 87.0% ± 0.6% | 87.0% ± 1.4% | 87.0% ± 1.4% | 86.9% ± 0.7% |
| R2 | 4.1% ± 6.0% | 13.4% ± 10.4% | 1.3% ± 2.1% | 7.3% ± 14.6% | neg | neg | 7.3% ± 14.6% |
| **Prostate Weight** | Random forest | Boosting | SVM | **NN** | Mean | Median | **Ridge**  **regression** |
| MAE | 19.51 ± 5.60 | 20.58 ± 6.13 | 18.13 ± 4.01 | 18.64 ± 2.08 | 18.79 ± 7.05 | 18.02 ± 6.85 | 18.41 ± 2.18 |
| RMSE | 27.38 ± 11.70 | 27.86 ± 12.75 | 25.46 ± 8.86 | 24.82 ± 5.23 | 26.77 ± 13.70 | 27.04 ± 13.84 | 24.54 ± 5.47 |
| 100-MAPE*100 | 64.1% ± 10.0% | 61.4% ± 9.2% | 63.8% ± 10.7% | 64.3% ± 5.7% | 62.3% ± 12.5% | 67.0% ± 11.4% | 64.7% ± 6.0% |
| R2 | 10.2% ± 9.5% | 10.2% ± 10.8% | 11.6% ± 15.2% | 13.1% ± 23.3% | neg | neg | 13.6% ± 22.9% |
| **Lymph Node Count** | Random forest | Boosting | SVM | NN | Mean | Median | Ridge  regression |
| MAE | 8.11 ± 1.04 | 7.60 ± 0.98 | 7.99 ± 1.22 | 9.32 ± 0.91 | 7.84 ± 1.03 | 7.71 ± 1.15 | 9.16 ± 0.91 |
| RMSE | 10.82 ± 1.34 | 10.25 ± 1.52 | 10.53 ± 1.53 | 11.80 ± 1.05 | 10.36 ± 1.47 | 10.34 ± 1.53 | 11.66 ± 1.05 |
| 100%-SMAPE | 80.6% ± 3.7% | 81.4% ± 2.5% | 80.6% ± 2.5% | 77.7% ± 2.3% | 80.9% ± 2.7% | 81.1% ± 2.5% | 78.0% ± 2.4% |
| R2 | neg | neg | neg | neg | neg | neg | neg |
| **Blood Loss** | Random forest | Boosting | SVM | NN | Mean | Median | Ridge  regression |
| MAE | 623 ± 123 | 540 ± 141 | 499 ± 150 | 701 ± 217 | 510 ± 129 | 488 ± 141 | 678 ± 205 |
| RMSE | 817 ± 193 | 743 ± 252 | 701 ± 271 | 879 ± 276 | 703 ± 235 | 688 ± 259 | 857 ± 266 |
| 100-MAPE*100 | 25.3% ± 23.5% | 43.1% ± 12.9% | 45.9% ± 15.3% | 26.7% ± 27.2% | 40.2% ± 17.1% | 46.5% ± 15.8% | 28.5% ± 25.5% |
| R2 | neg | neg | neg | neg | neg | neg | neg |
| **Intraop. Fluid Admin.** | Random forest | Boosting | SVM | NN | Mean | Median | Ridge  regression |
| MAE | 1281 ± 397 | 1159 ± 503 | 1154 ± 478 | 1474 ± 399 | 1157 ± 492 | 1094 ± 523 | 1441 ± 407 |
| RMSE | 1601 ± 607 | 1462 ± 678 | 1474 ± 647 | 1911 ± 641 | 1461 ± 661 | 1413 ± 689 | 1870 ± 645 |
| 100-MAPE*100 | 60.6% ± 5.1% | 64.1% ± 9.8% | 64.0% ± 8.2% | 55.3% ± 12.3% | 62.5% ± 10.6% | 66.0% ± 9.9% | 56.2% ± 11.5% |
| R2 | neg | neg | neg | neg | neg | neg | neg |
|  |  |  |  |  |  |  |  |
| **Postop.Hct.** | Random  forest | Boosting | SVM | **NN** | Mean | Median | **Ridge**  **regression** |
| MAE | 3.10 ± 0.18 | 3.25 ± 0.48 | 3.12 ± 0.34 | 3.05 ± 0.36 | 3.47 ± 0.49 | 3.51 ± 0.55 | 3.04 ± 0.36 |
| RMSE | 3.86 ± 0.34 | 4.05 ± 0.60 | 3.94 ± 0.49 | 3.81 ± 0.56 | 4.10 ± 0.49 | 4.16 ± 0.57 | 3.81 ± 0.56 |
| 100-MAPE*100 | 89.6% ± 0.8% | 89.1% ± 1.5% | 89.5% ± 1.4% | 89.7% ± 1.7% | 88.3% ± 1.3% | 88.4% ± 1.5% | 89.7% ± 1.7% |
| R2 | 13.3% ± 14.9% | 7.6% ± 11.2% | 16.6% ± 15.3% | 21.6% ± 18.3% | neg | neg | 21.7% ± 18.3% |
| **Postop.Hb.** | **Random**  **forest** | Boosting | SVM | NN | Mean | Median | Ridge  regression |
| MAE | 1.04 ± 0.13 | 1.13 ± 0.12 | 1.08 ± 0.18 | 1.10 ± 0.09 | 1.27 ± 0.11 | 1.28 ± 0.13 | 1.11 ± 0.09 |
| RMSE | 1.29 ± 0.21 | 1.34 ± 0.18 | 1.33 ± 0.27 | 1.36 ± 0.15 | 1.47 ± 0.17 | 1.50 ± 0.20 | 1.37 ± 0.15 |
| 100-MAPE*100 | 89.9% ± 1.3% | 89.1% ± 1.0% | 89.5% ± 1.8% | 89.3% ± 0.8% | 87.7% ± 0.8% | 87.8% ± 0.9% | 89.2% ± 0.8% |
| R2 | 16.0% ± 13.1% | 11.0% ± 8.1% | 13.3% ± 13.6% | 11.6% ± 12.8% | neg | neg | 11.0% ± 12.6% |

**(A) Regression Tasks**

| **Frozen Section Result** | Random forest | Boosting | SVM | **NN** | Mode | **Logistic**  **regression** |
| --- | --- | --- | --- | --- | --- | --- |
| Accuracy | 55.4% ± 9.3% | 49.9% ± 10.4% | 55.4% ± 12.1% | 56.4% ± 8.1% | 59.6% ± 10.2% | 54.2% ± 12.8% |
| AUC | 55.4% ± 8.6% | 53.0% ± 8.0% | 57.0% ± 15.1% | 65.0% ± 6.1% | N/A | 65.6% ± 8.1% |
| Sensitivity | 33.9% ± 6.0% | 31.2% ± 5.9% | 34.2% ± 3.8% | 33.7% ± 7.2% | 36.7% ± 6.7% | 34.5% ± 10.7% |
| Specificity | 67.6% ± 4.6% | 67.0% ± 4.7% | 68.5% ± 3.8% | 68.1% ± 4.8% | 63.3% ± 6.7% | 68.7% ± 8.6% |
| PPV | 28.5% ± 10.1% | 29.7% ± 7.0% | 28.0% ± 9.7% | 33.8% ± 10.6% | 22.0% ± 5.9% | 36.1% ± 11.5% |
| NPV | 64.8% ± 12.0% | 66.7% ± 5.4% | 63.2% ± 12.2% | 73.5% ± 10.2% | 48.6% ± 9.2% | 71.1% ± 11.8% |
| **FS Decision (yes/no)** | **Random forest** | Boosting | SVM | **NN** | Mode | **Logistic**  **regression** |
| Accuracy | 59.7% ± 11.0% | 57.6% ± 11.6% | 64.9% ± 10.4% | 60.6% ± 7.8% | 59.6% ± 10.2% | 59.6% ± 9.1% |
| AUC | 55.6% ± 11.7% | 55.9% ± 15.4% | 38.8% ± 13.4% | 60.3% ± 9.7% | N/A | 62.0% ± 10.5% |
| Sensitivity | 78.1% ± 16.0% | 67.7% ± 14.7% | 77.9% ± 20.0% | 81.2% ± 12.3% | 100.0% ± 0.0% | 74.8% ± 13.8% |
| Specificity | 31.2% ± 8.0% | 43.1% ± 12.5% | 41.4% ± 16.6% | 31.3% ± 9.3% | 0.0% ± 0.0% | 35.7% ± 12.9% |
| PPV | 62.2% ± 12.3% | 63.6% ± 8.5% | 66.2% ± 6.2% | 63.2% ± 9.5% | 59.6% ± 10.2% | 62.9% ± 10.9% |
| NPV | 57.5% ± 26.9% | 50.1% ± 23.2% | 67.8% ± 22.3% | 54.7% ± 19.9% | 0.0% ± 0.0% | 50.9% ± 18.9% |

**(B) Classification Tasks**

**Supplementary Table S8:** **Performance metrics — ORP subgroup, without nerve-sparing parameters as input features** The table summarizes the predictive performance of four machine learning models (Random Forest, Gradient Boosting, Support Vector Machine [SVM], and Neural Network [NN]) compared to baseline models (Mean/Median for regression, Mode for classification, and multivariable Ridge regression). Performance was evaluated using a 5-fold cross-validation scheme. **(A) Regression Tasks:** Model performance was assessed using MAE, RMSE, SMAPE-based Accuracy (100% − SMAPE) for outcomes with zero values where MAPE is undefined, and R². Lower MAE and RMSE values indicate better performance; higher SMAPE-based Accuracy and R² values indicate better model fit. An R² value of 'neg.' indicates performance worse than the mean predictor. **(B) Classification Tasks:** Model performance was assessed using Accuracy, AUC, Sensitivity, Specificity, PPV, and NPV. For the Frozen Section Result outcome, macro-averaged one-vs-rest metrics are reported. **Abbreviations:** AUC, Area Under the ROC Curve; FS, Frozen Section; MAE, Mean Absolute Error; NN, Neural Network; NPV, Negative Predictive Value; PPV, Positive Predictive Value; R², Coefficient of Determination; RMSE, Root Mean Squared Error; SMAPE, Symmetric Mean Absolute Percentage Error; SVM, Support Vector Machine.

**Supplementary Figures**

**Supplementary Figure S1a:** SHAP feature importance — RARP subgroup, including nerve-sparing parameters, primary outcomes (FS Decision, Postoperative Hemoglobin, Hb Drop, Length of Hospital Stay).

**Supplementary Figure S1b:** SHAP feature importance — RARP subgroup, including nerve-sparing parameters, secondary outcomes (Postoperative ISUP Grade, Blood Loss, Prostate Weight).

Each panel displays the normalized mean absolute SHAP attribution value (%) for each preoperative feature, derived from the best-performing model per outcome. Red bars indicate nerve-sparing-related features; blue bars indicate all other preoperative features. Panels: (A) FS Decision (Random Forest), (B) Postoperative Hemoglobin (SVM), (C) Hb Drop (Random Forest), (D) Length of Hospital Stay (Gradient Boosting), (E) Postoperative ISUP Grade (Neural Network), (F) Blood Loss (Neural Network), (G) Prostate Weight (Random Forest). Abbreviations: FS, Frozen Section; Hb, Hemoglobin; ISUP, International Society of Urological Pathology; SHAP, SHapley Additive exPlanations; SVM, Support Vector Machine.

**Supplementary Figure S2a:** SHAP feature importance for the prediction of postoperative outcomes — RARP subgroup, excluding nerve-sparing parameters as input features, primary outcomes. Each panel displays the normalized mean absolute SHAP attribution value (%) for each preoperative feature, derived from the best-performing model per outcome. Panels: (A) FS Decision (Random Forest), (B) Postoperative Hemoglobin (SVM), (C) Hb Drop (Gradient Boosting), (D) Length of Hospital Stay (Gradient Boosting). Abbreviations: FS, Frozen Section; Hb, Hemoglobin; SHAP, SHapley Additive exPlanations; SVM, Support Vector Machine.

**Supplementary Figure S2b:** SHAP feature importance for the prediction of postoperative outcomes — RARP subgroup, excluding nerve-sparing parameters as input features, secondary outcomes. Panels: (E) Postoperative ISUP Grade (Neural Network), (F) Blood Loss (Neural Network), (G) Prostate Weight (Random Forest). Abbreviations: ISUP, International Society of Urological Pathology; SHAP, SHapley Additive exPlanations.

**Supplementary Figure S3a:** SHAP feature importance for the prediction of postoperative outcomes — ORP subgroup, including nerve-sparing parameters as input features, primary outcomes. Each panel displays the normalized mean absolute SHAP attribution value (%) for each preoperative feature, derived from the best-performing model per outcome. Red bars indicate nerve-sparing-related features; blue bars indicate all other preoperative features. Panels: (A) FS Decision (Neural Network), (B) Postoperative Hemoglobin (Random Forest), (C) Hb Drop (Gradient Boosting), (D) Length of Hospital Stay (Gradient Boosting). Abbreviations: FS, Frozen Section; Hb, Hemoglobin; SHAP, SHapley Additive exPlanations.

**Supplementary Figure S3b:** SHAP feature importance for the prediction of postoperative outcomes — ORP subgroup, including nerve-sparing parameters as input features, secondary outcomes. Red bars indicate nerve-sparing-related features; blue bars indicate all other preoperative features. Panels: (E) Postoperative ISUP Grade (Random Forest), (F) Blood Loss (SVM), (G) Prostate Weight (SVM). Abbreviations: ISUP, International Society of Urological Pathology; SHAP, SHapley Additive exPlanations; SVM, Support Vector Machine.

**Supplementary Figure S4a:** SHAP feature importance for the prediction of postoperative outcomes — ORP subgroup, excluding nerve-sparing parameters as input features, primary outcomes. Each panel displays the normalized mean absolute SHAP attribution value (%) for each preoperative feature, derived from the best-performing model per outcome. Panels: (A) FS Decision (Neural Network), (B) Postoperative Hemoglobin (Random Forest), (C) Hb Drop (Gradient Boosting), (D) Length of Hospital Stay (Gradient Boosting). Abbreviations: FS, Frozen Section; Hb, Hemoglobin; SHAP, SHapley Additive exPlanations.

**Supplementary Figure S4b:** SHAP feature importance for the prediction of postoperative outcomes — ORP subgroup, excluding nerve-sparing parameters as input features, secondary outcomes. Panels: (E) Postoperative ISUP Grade (Random Forest), (F) Blood Loss (SVM), (G) Prostate Weight (Neural Network). Abbreviations: ISUP, International Society of Urological Pathology; SHAP, SHapley Additive exPlanations; SVM, Support Vector Machine.
